# Optimized Hemoglobin Depletion Improves Clinical Proteomics from Dried Blood Spots

**DOI:** 10.1021/acs.jproteome.6c00534

**Published:** 2026-10-16

**Authors:** Hannah Ging, Rosemary E. Maher, Elin Davies, Philip J. Brownridge, Anirudh Rao, Alan D. Salama, Louise Oni, Claire E. Eyers, Andrew J. Chetwynd

**Affiliations:** † Centre for Proteome Research, Faculty of Health & Life Sciences, 4591 University of Liverpool, Liverpool L69 7ZB, United Kingdom; ‡ Department of Biochemistry, Cell and Systems Biology, Institute of Systems, Molecular and Integrative Biology, University of Liverpool, Liverpool L69 7BE, United Kingdom; § U.K.’s Experimental Arthritis Treatment Centre for Children, University of Liverpool, Liverpool L14 5AB, United Kingdom; ∥ Department of Nephrology, 4595 Liverpool University Hospitals NHS Foundation Trust, Liverpool L7 8YE United Kingdom; ⊥ Department of Women’s and Children’s Health, Institute of Life Course and Medical Sciences, University of Liverpool, Liverpool L7 8TX, United Kingdom; # Department of Renal Medicine, University College London, UCL Medical School, Rowland Hill Street, London NW3 2PF, United Kingdom; ∇ Department of Paediatric Nephrology, Great Ormond Street Hospital for Children, London WC1N 3JH, United Kingdom

**Keywords:** microsampling, kidney disease, LC-MS/MS, hemoglobin, DBS, plasma, serum

## Abstract

Equitable access to large sample cohorts for robust, high-throughput proteomics for biomarker discovery is a major barrier to widescale clinical implementation. Dried blood spots (DBS) offer a minimally invasive alternative to venous blood draws, enabling at-home microsampling (<50 μL) for centralized analysis, thus enhancing research participation. This approach is particularly relevant for under-represented groups, including children, the elderly, minority backgrounds and those with long-term health conditions such as chronic kidney disease (CKD), where disease fluctuations may occur outside the clinic, and vein preservation is critical. Proteomic analysis has demonstrated great utility in monitoring disease progression, and for biomarker/therapeutic target discovery. However, liquid chromatography–tandem mass spectrometry (LC–MS/MS) of whole blood is hindered by the wide dynamic range and the relatively high-abundance of proteins such as hemoglobin, compromising biomarker discovery. Here, we establish an optimized workflow for protein extraction and hemoglobin depletion from microsamples obtained using DBS, enabling sensitive and high-throughput proteomic analysis. We demonstrate that hemoglobin depletion increases protein identifications by ∼50%, mitigating ion suppression and dynamic range effects, enabling the identification of putative biomarkers from patients with stage 5 CKD on dialysis. We also evaluated a commercial cell-free DBS device which yielded a sample more representative of plasma compared to traditional DBS and enabled greater depletion of hemoglobin compared to traditional DBS with hemoglobin depletion methods. Our findings offer a scalable approach for biomarker discovery, facilitating remote, longitudinal clinical studies.

## Graphical abstract

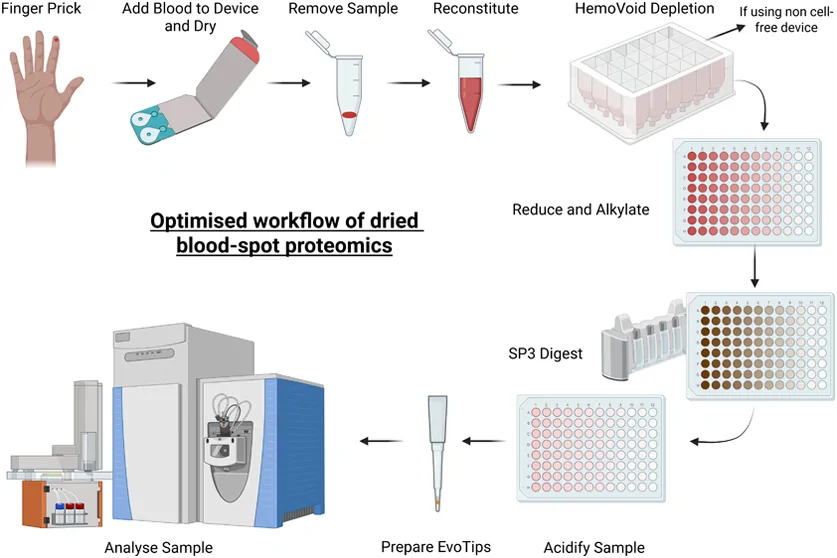

## Introduction

The rapid advances seen in the precise genomic characterization of genetic diseases has highlighted the need to advance the field of clinical proteomics to improve characterization of diseases that may be driven by, or caused by, protein-based abnormalities that do not necessarily have a genetic component.^1,2^ Clinical proteomics thus shows significant promise for both advancing the understanding of disease pathogenesis and potentially highlighting novel therapeutic targets.^3–7^ Typically, clinical proteomics utilizes fresh plasma or serum samples that are processed on site and stored for batch analysis, requiring specialized laboratory equipment and on-site phlebotomists, with potential for substantial variability depending on the local processing procedures.^3,8,9^

There is growing interest in the use of “microsampling”, where the volume collected is typically 50 μL or less,^10^ to overcome these limitations. An example of microsampling is the collection of dried blood spots (DBS), typically from a capillary finger-prick. DBS offer several advantages over traditional venipuncture given their compatibility with remote, microvolume, longitudinal sampling, low-cost, and the fact that they are significantly less invasive than venipuncture.^11,12^ Studies also show that DBS are preferred to venipuncture by patient populations as they reduce the likelihood of negative reactions such as dizziness and sampling fatigue.^13,14^ Additionally, such microsampling aligns with the “Save the Vein” initiative that is particularly important when monitoring long-term health conditions such as chronic kidney disease (CKD) where preservation of vein health is important for the formation of arterio-venous fistula for hemodialysis access.^15^ DBS have been used to understand viral infection and drug dosing, following interrogation of the proteome and/or the metabolome.^16–19^ However, widespread clinical application of DBS sampling is not yet routine, with a major challenge being the variability in interference from cellular components when blood cells hemolyse upon drying,^20,21^ along with a lack of standardization in sample processing methodologies such as DBS device and sample reconstitution.^11^

Currently the two DBS devices most commonly in clinical use are the Whatman903 and Ahlstrom226 filter papers. However, these are not volumetric and therefore pose a risk of quantitative inaccuracy.^22,23^ Volumetric sampling devices are now growing in popularity, allowing for more regulated collection of DBS, eliminating dependency on even and consistent spreading of blood across the device, and therefore the impact of the hematocrit effect on sample volume as is seen with traditional filter papers, with demonstrable application across a number of fields, particularly pharmacology,^24–28^ and ‘omics.^29–34^

A notable challenge in clinical proteomics is the broad dynamic range of protein concentration in blood products, with hemoglobin accounting for 95% of the protein load in whole blood,^35^ while albumin, globulins and fibrinogens are together reported to make up 99% of the protein concentration in plasma.^36,37^ These high-abundance proteins can compromise the ability to identify candidate biomarkers due to ion suppression of lower abundance analytes during electrospray.^36,38,39^ To overcome this barrier in standard serum/plasma clinical proteomics workflows, depletion of hemoglobin and other highly abundant proteins, such as albumin, has traditionally been achieved using affinity binding mechanisms.^40–42^ Depletion of hemoglobin from reconstituted DBS is thus perceived to be a crucial step in establishing a sensitive DBS proteomics workflow for clinical application, which has to date hampered their use as convenient biomarker discovery vehicles.^16,43,44^ An alternative approach is the use of “cell-free” DBS devices, from companies such as Biodesix,^45^ Cobas,^46^ and more recently Capitainer, all of which remove blood cells and thus hemoglobin. These devices primarily operate via microfluidic separation^47–49^ although passive filtration is also used.^50,51^ Their ability to remove hemoglobin-containing cells at the point of sampling could provide broader use of DBS for clinical investigation.

Here we have evaluated and optimized four different commercial DBS devices for sensitive, robust label-free quantitative proteomics, considering reconstitution buffer, microsampling device, differences between plasma and blood, and the impact of hemoglobin depletion, comparing with more routine, liquid based sample processing. As proof-of-principle, we applied our optimized workflow to explore the changes in the proteome profiles of DBS from a cohort of patients with stage five CKD (CKD5).

## Materials and Methods

Unless otherwise specified, all reagents were sourced from ThermoFisher Scientific, Waltham, MA, United States.

### Ethical and Regulatory Approvals

For method development, samples were collected from healthy participants at the University of Liverpool (IRAS project identification: 327208). A total of 10 patients with stage 5 CKD and 10 healthy control adults were recruited in the proof-of-principle clinical validation aspect of this project using the ethically approved GlomOmics study (REC 23/PR/0490).

### Participants and Biosample Collection

Healthy participants were consented and recruited to support the initial method development using fresh heparinized whole blood taken from two healthy adult controls—one male and one female, ages 34 and 38. Participants with CKD5 managed on hemodialysis at the Royal Liverpool University Hospital, (Liverpool, UK) were recruited for clinical proof-of-principle immediately prior to starting a hemodialysis session. Demographic data were collected on all participants and all patients provided informed written consent, with samples handled under the principles of the Declaration of Helsinki. In all steps of the method development, 5 replicates were analyzed.

### Dried Blood-Spot Devices and Sample Collection

The DBS devices used in this study were: 10 μL volumetric b10 and Sep10 (Capitainer Ab, Solna, Sweden), Whatman903 (Sigma-Aldrich, St Louis, MO, United States), and 10 μL VAMS Mitra devices (Neoteryx, Torrance, CA, United States). For healthy control DBS, venous blood was collected in lithium heparin vacutainers and 10 μL was pipetted directly onto the B10, and Whatman903 devices. Sampling for the 10 μL VAMS Mitra devices was achieved by holding the device to the surface of the blood sample until saturated. For Sep10 devices, 70 μL of blood was pipetted directly onto the device, which was then tilted to 90° for 2 s. After in-device cell filtration, 10 μL of plasma-like solution was deposited on a collection disk. Samples were then allowed to dry for 3 h in a fume hood, or overnight for the Sep10 devices as per manufacturer’s instruction.

For all optimization, freshly drawn blood samples were used for each experiment, accounting for interexperiment variation, as each experiment used different blood samples. Proof-of-principle clinical samples were obtained using capillary sampling using a finger prick device (BD Microtainer, Fisher Scientific, Loughborough, UK)

### Sample Reconstitution

DBS samples were reconstituted using four different solutions: (i) 200 μL of 50 mM Ammonium Bicarbonate (AmBic),^16^ (ii) 10 mM Phosphate Buffered Saline (PBS),^52^ (iii) 0.1% (v/v) Trifluoroacetic Acid (TFA)^53^ in HPLC grade water, or (iv) 67% 100 mM AmBic and 33% 2,2,2-Trifluoroethanol (TFE)^29^ and incubated for 2 h in a ThermoMixer (ThermoFisher Scientific, Waltham, MA, United States) at 800 rpm at room temperature (RT). The reconstituted sample was centrifuged at 700*g*, 4 °C for 15 min. The supernatant was then removed and stored in a fresh 1.5 mL tube. Alternatively, 133 μL of 50 mM AmBic and 67 μL TFE was added to the DBS in a microfuge tube and sonicated (15 min, RT), before incubation for 30 min in a shaking ThermoMixer (1000 rpm, RT). Samples were centrifuged at 700*g*, 4 °C for 15 min, the supernatant collected and stored in a fresh low-bind 1.5 mL tube.^29^

A lysis bead reconstitution was also evaluated whereby HPLC-grade water (500 μL) was added to the DBS and homogenized with CKMix beads (Bertin Technologies SAS, Montigny-le-Bretonneux, France) in a Minilys Personal Homogenizer (Bertin Technologies SAS, Montigny-le-Bretonneux, France) for 5 min at RT.^54^ To the lysed sample, 500 μL Tris HCl (pH 8.5) and 0.5% (w/v) sodium dodecyl sulfate (SDS) were added. The sample was sonicated (100 W Cavitek Sonicator, Cavitek, Allendale Ultrasonics, Hertfordshire, UK) on full power for 5 min at RT. The sample was centrifuged at 700*g*, 4 °C for 15 min, the supernatant collected and stored in a fresh low-bind 1.5 mL tube.

In all cases, reconstitution was performed directly prior to further sample processing and analysis.

### High-Abundance Protein Depletion

To deplete hemoglobin, HemogloBind Depletion and NuGel-HemogloBind Depletion (both 20 μL reconstituted blood) were performed as per manufacturer’s instructions (see Supporting Information).

HemoVoid depletion was performed as per manufacturer’s directions, with all measurements halved to account for the reduced blood volume of 150 μL of reconstituted sample (see Supporting Information).

### Protein Digestion

Reconstituted samples (25 μL) were digested using a modified SP3 protocol, and samples were washed with ethanol.^55,56^ Samples were reduced and alkylated at 95 °C for 5 min with Tris­(2-carboxyethyl)­phosphine (TCEP)/chloracetamide (CAA) (120 mM/480 mM) (ThermoFisher Scientific, Waltham, MA, United States), then digested on E3 and E7 Sera-Mag SP3 beads (Cytiva, Marlborough, MA, United States) using LysC (Promega, Madison, WI, United States) for 2 h, followed by Trypsin (Promega, Madison, WI, United States) for 16 h at a 50:1 Protein: Protease ratio for each protease, at 1400 rpm and 37 °C (see Supporting Information). Following digest, samples were acidified with formic acid to a final concentration of 1% (v/v) and centrifuged for 5 min at 2300*g*. The supernatant was taken and dried to dryness using a SpeedVac (Centrifuge: UNIVAPO—150 ECH, Cooling unit: UNICRYO MC2L −60 °C, Vacuum pump: UNIVAC DQ4) prior to freezing at −80 °C until LC-MS/MS analysis. In all cases, 5 experimental replicates were processed.

### LC-MS/MS

The dried peptides were resuspended in 100 μL 0.1% (v/v) FA, before diluting to a final peptide concentration of 5 ng/μL, determined using a NanoDrop 2000c (ThermoFisher Scientific, Waltham, MA, United States). EvoTips (EvoSep, Odense, Denmark) were prepared as per the manufacturer’s instructions (see Supporting Information).

Peptides were separated using an EvoSep One LC system, applying a 30 samples per day method (see Supporting Methods) on a C18 EASY-Spray HPLC column (2 μm particle size, 100 Å pore size, 150 μm × 150 mm). Solvent A was water with 0.1% (v/v) FA and Solvent B was acetonitrile with 0.1% (v/v) FA, freshly prepared for each experiment. Separated peptides were analyzed using a QEx HF Orbitrap in positive electrospray ionization (ESI) mode. The MS1 scan range of 350–2000 *m*/*z*, with an automatic gain control (AGC) of 3E6, a maximum fill time of 100 ms, and a mass resolution of 60,000 fwhm at *m*/*z* 200. MS2 was acquired using a top 18 data-dependent acquisition (DDA) approach, between 200 to 2000 *m*/*z*, with a normalized collision energy (NCE) of 29% at a resolution of 30,000, an AGC of 1E5, and a maximum injection time of 45 ms.

### Data Analysis

Data was processed using Proteome Discoverer v2.4.0.305 (ThermoFisher Scientific, Waltham, MA, United States), searching against the UniProt human proteome database [accessed November 2025], using the MASCOT search engine. Precursor ion error tolerance was set at 10 ppm, and fragment ion error tolerance at 0.01 Da. Fixed modifications were set for carbamidomethylation of cysteine, and variable modifications for oxidation of methionine. The false discovery rate (FDR) was set at 1% at the level of the peptide spectral match. Data was then processed, log2 transformed and proteins absent in ≥25% of samples across all conditions excluded, before median normalization and knn = 3 imputation. Data analysis, statistical analyses and data visualization were performed in R (RStudio v4.2.0). Differences in the total number of proteins identified between groups was calculated using the Kruskal–Wallis test. Where significant effects were observed, post hoc pairwise comparisons were performed using Dunn’s test, with p-values adjusted using the Holm-Bonferroni correction method. Percentage coefficients of variation (CV) were calculated from normalized, transformed values. Differential protein abundance analysis was performed using the Limma package. A linear model without an intercept term was fitted to the log2-transformed protein abundance values. Pairwise contrasts were evaluated using empirical Bayes moderation, and resulting p-values adjusted for multiple testing using Benjamini-Hochberg. Gene Ontology (GO) enrichment analysis was performed using ClusterProfiler.

## Results and Discussion

### Optimization of Sample Reconstitution Buffer

To compare the depth of the DBS proteome across the three DBS devices five reconstitution methods were evaluated, employing either AmBic, PBS, Lysis Beads, TFA, or TFE (Figure 1). Proteome coverage was evaluated by considering the number and reproducibility of proteins identified, as well as the consistency of quantification across all replicates.

**Figure 1:**
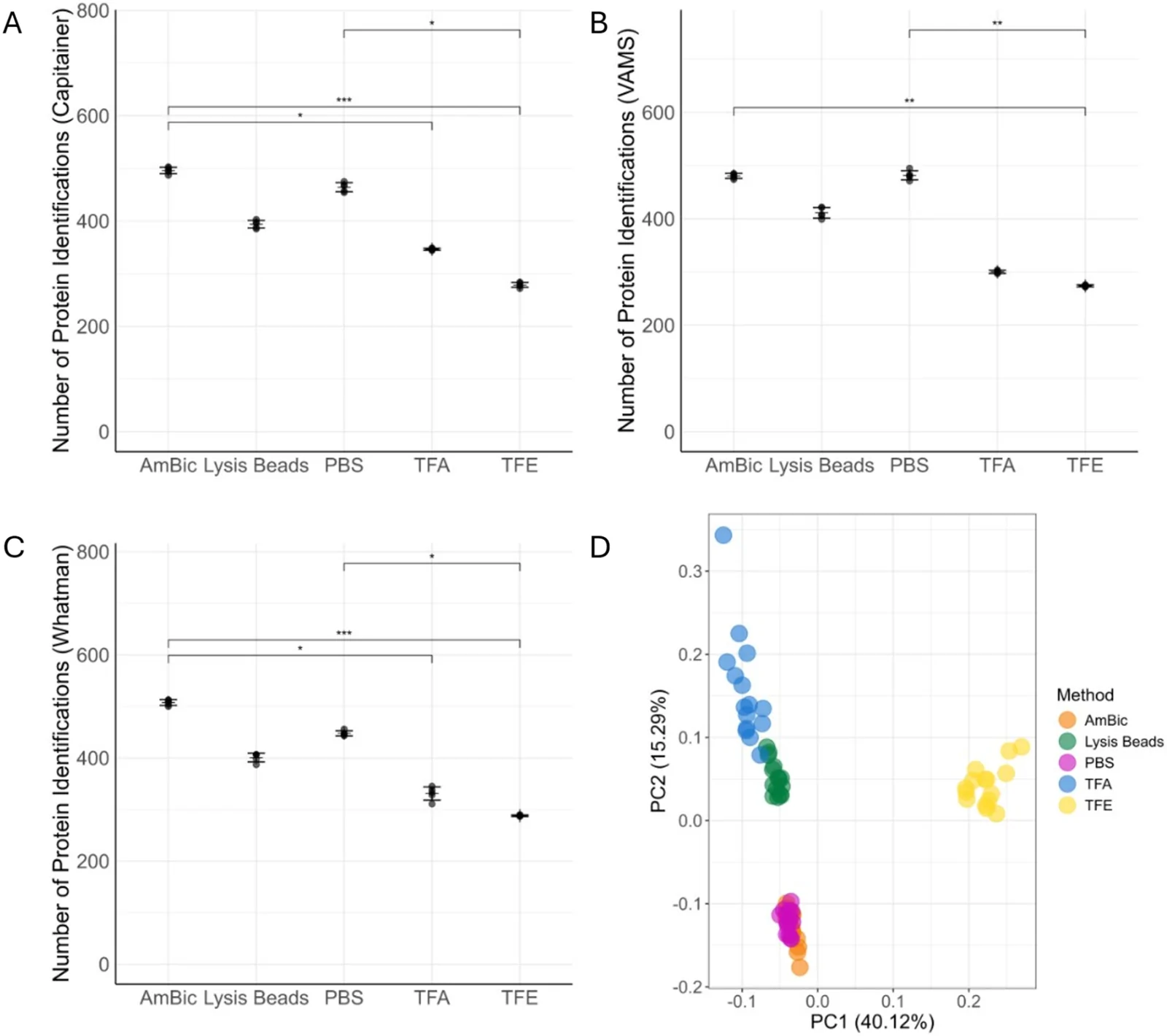
Comparison of reconstitution methods and DBS devices. (A) The average number of proteins identified in Capitainer b10 DBS with each reconstitution method (*n* = 5). (B) The average number of proteins identified in the Neoteryx VAMS Mitra DBS based on the reconstitution method (*n* = 5). (C) The average number of proteins identified in Whatman903 DBS with each reconstitution method (*n* = 5). (D) The data set (*n* = 5, total samples= 75) was subjected to PCA using the dried blood spot proteome, and color coded by reconstitution buffer. *= *p* < 0.05, **= *p* < 0.01, ***= *p* < 0.001, Error Bars= Standard Deviation. AmBic= Ammonium Bicarbonate, PBS= Phosphate Buffered Saline, TFA= Trifluoroacetic Acid, TFE= Tetrafluoroethylene.

No significant difference was observed in the total numbers of proteins identified using either AmBic or PBS as reconstitution buffers, and across all devices 495 ± 13 and 465 ± 16 proteins respectively were identified (Figure 1A–C). Slightly lower numbers of proteins were identified with the other reconstitution methods across all devices (Figure 1A–1C) at 402 ± 11, 326 ± 21 and 280 ± 7 for the lysis beads, TFA and TFE respectively. TFA yielded significantly fewer protein identifications than AmBic when using the Capitainer and Whatman devices (both *p* = 1.1 × 10^–2^). However, only the TFE reconstitution returned a statistically significantly lower number of protein identifications compared to both AmBic and PBS across all three tested devices. The number of proteins identified was reproducible for AmBic and PBS, with Coefficients of Variation (CVs) of 1.3% and 1.8%, respectively across all devices (Figure 1A–C). When examining this at a peptide level, the same findings remained, with AmBic reconstitution yielding 4176 ± 46 peptide identifications across all devices, compared to 4005 ± 78, 4009 ± 74, 3197 ± 75, and 3084 ± 35 when using lysis beads, PBS, TFA, and TFE respectively (Supporting Figure S1). Across all three devices, protein identities were highly reproducible using both AmBic and PBS (Supporting Figure S2), with over 90% of proteins being seen in all 5 replicates, for all three devices.

Reproducibility of protein quantification was assessed across all replicates per condition (Supporting Figure S3). AmBic and PBS returned the lowest median CVs of 0.66 ± 0.92% (Capitainer), 0.50 ± 0.64% (VAMs), and 0.51 ± 0.74% (Whatman903) for AmBic, and 0.57 ± 0.83% (Capitainer), 0.57 ± 1.00% (VAMs), and 0.55 ± 0.78% (Whatman903), for PBS. This was also seen at a peptide level (Supporting Figure S4) with AmBic resulting in median CVs of 1.32 ± 2.10% (Capitainer), 1.13 ± 1.69% (VAMs), and 1.11 ± 1.90% (Whatman903), and PBS at 1.26 ± 1.96% (Capitainer), 1.22 ± 2.02% (VAMs), and 1.11 ± 1.88% (Whatman903), for PBS. This consistency was further highlighted through PCA visualization, with replicates for AmBic and PBS extraction being tightly clustered (based on both PC1 and PC2) indicating the similarity of their identified proteomes (Figure 1D). Evaluation of peptide- and protein-level missingness indicated comparable reproducibility of coverage across all devices and reconstitution methods, ranging from 2.2 to 9.7% at the peptide level and 0–7.5% at the protein level. Only the TFA method using Whatman filter papers showed elevated values, ranging from 4.9% to 21.1% missing values at peptide-level, compared to 2.4–8.3% for all other method-device combinations, a pattern that was mirrored at the protein level (Supporting Figures S5 and S6).

Due to the numbers of proteins identified, the reproducibility of quantification, and compatibility with downstream processing pipelines, 50 mM AmBic was selected as the optimal reconstitution buffer.

### Comparison of Micro-Sampling Devices

Of the three DBS devices assessed: b10, VAMS Mitra, and Whatman903, the Whatman903 device yielded marginally higher protein identification numbers at 508 ± 6, compared to 496 ± 6 and 481 ± 5 for b10 and VAMS, respectively. All devices had CV < 1.5% showing remarkable reproducibility, and there were no significant differences in the number of proteins identified between the devices when replicated (*n* = 5) (*p* > 0.999 for all) (Figure 1A–1C). Unlike the Whatman903 devices, VAMS and b10 devices are volumetric, accurately collecting a defined volume of blood, which supports reliability across the sample cohorts.^22,23^ Furthermore, for shipping remote samples, the b10 and the VAMs devices provide a self-contained sample for convenience which is protected from damage and contamination.

Previous studies comparing the clinical performance of VAMS and b10 devices concluded that the b10 devices were the most consistent in terms of sampling success, with 92–96% of b10 samples being usable to monitor rejection drug concentration in transplant recipients.^57^ The study reported that comparatively usability of VAMS devices peaked at 72–88% when the sample was taken by a healthcare professional, and dropped 52–72% when the patient performed the sampling using the VAMs Mitra devices.^57^ Importantly, a patient engagement and acceptability study identified that the environmental impact of the microsampling device is an important consideration, and thus the primarily cardboard b10 devices provide a more environmentally sustainable option.^14^ As such, the b10 devices were selected for further optimization.

As a negative control, blank DBS devices were also analyzed to evaluate potential contamination. Following exclusion of those proteins that were present in <25% of samples across all devices, only 44 proteins were identified consistently across the three devices. Keratins made up 13 of these 44 proteins, accounting for ≈85% of the relative protein abundance, calculated using the intensities of each signal, with other proteins including albumin, dermcidin, actin, and two immunoglobulin proteins (For full list of identified proteins, see Supporting Data Sheet 2).

### Comparison of Plasma, Dried Plasma and Dried Blood

To enable routine clinical sampling for proteomics, it is important to determine how closely the protein composition of DBSs represent the current gold standard of plasma. Comparing dried plasma spots with DBS (both processed in the same manner) and traditional plasma samples, we identified 392 proteins unique to the DBS (Wet plasma= 181 ± 3, Dried plasma spot = 209 ± 3, Dried blood spot= 573 ± 2, *p* = 1.2 × 10^–3^) (Figure 2A,B), primarily due to the presence of cellular proteins in whole blood DBS.^58^ The 1.7-fold and 2.2-fold increase in DBS protein identifications compared with either dried or wet plasma (respectively), was greater than that observed at the peptide level (Wet plasma= 2504 ± 61, Dried plasma spot= 2653 ± 42, Dried blood spot = 4192 ± 60, equating to 0.7- and 0.6-fold increase respectively), indicating that more proteins were being identified with fewer unique peptides, suggesting lower coverage of lower abundance proteins (Supporting Figure S7). Gene ontology (GO) analysis confirmed that most of the proteins unique to DBS are cellular compartment proteins, particularly cytosolic proteins (Figure 2C) (Supporting Data Sheet 3). These data mirror the findings of Molloy et al, 2022, who identified similar differences in protein number and composition between dried plasma and blood samples using VAMS devices.^43^

**Figure 2:**
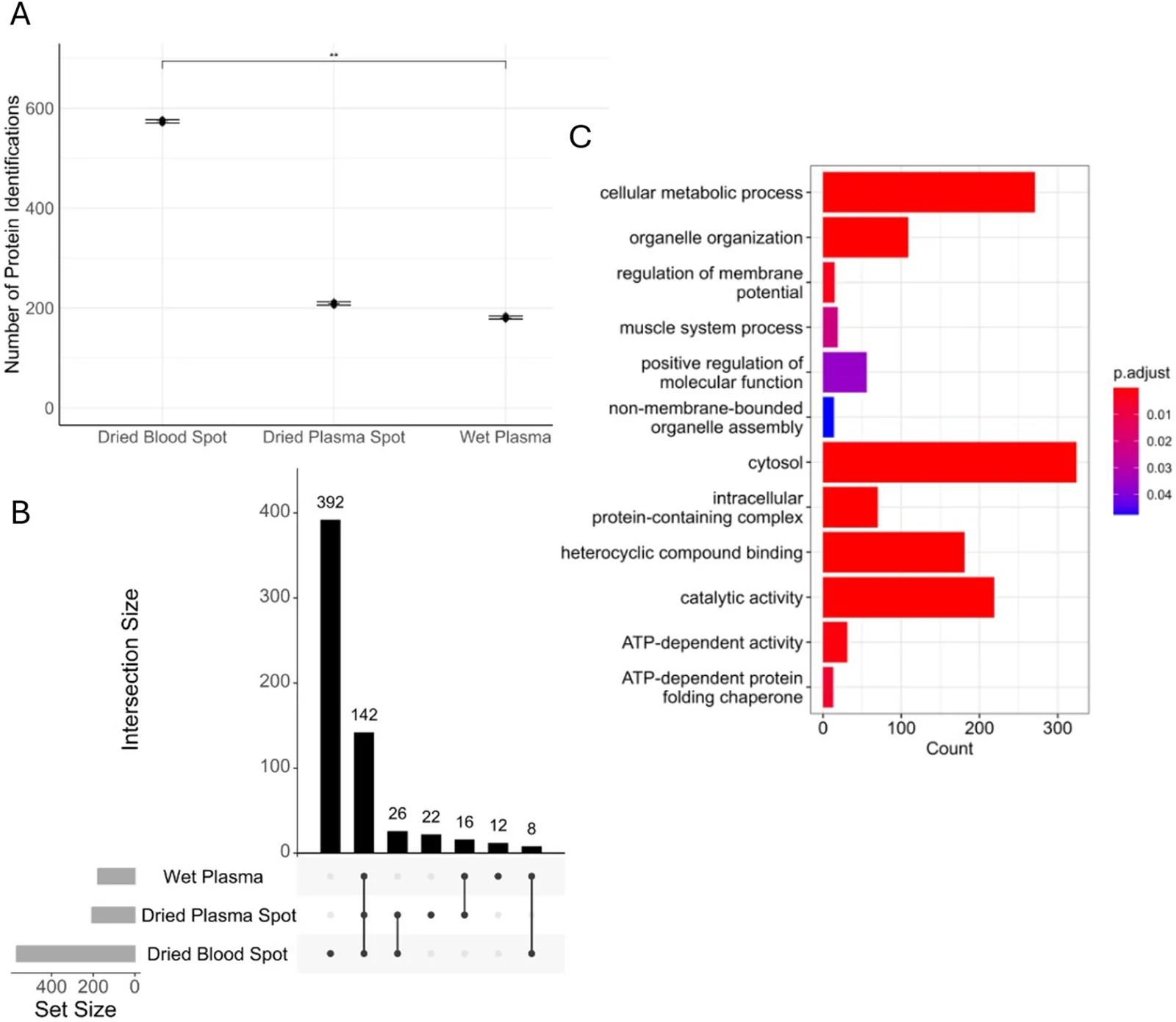
Comparison of DBS with dried plasma and standard plasma collection (A) The average number of proteins identified in dried blood spots, dried plasma spots, and wet plasma. (*n* = 5). (B) Upset plot exploring the similarities and differences between the proteins identified across sample types. (C) Gene ontology analysis of proteins only found to be present in the dried blood spot proteome, when compared to both the dried plasma spot and wet plasma proteomes. **= *p* < 0.01, Error Bars = Standard Deviation.

When comparing peptide intensity distributions across sample types to assess dynamic range (Supporting Figure S8), DBS showed the highest median protein intensity, reflecting the additional high-abundance cellular protein content captured in whole blood. Wet plasma, however, displayed the broadest dynamic range, followed by DBS and dried plasma spot (Supporting Figure S8). This indicates that while DBS captures a greater proportion of high-abundance material, wet plasma quantification spans a broader range of protein abundance.

There was <15% difference in the number of proteins identified using wet versus dry plasma, showing no statistical significance (*p* = 0.15) (Figure 2A, Supporting Data Sheet 4). Twelve proteins were exclusively identified in the wet plasma samples, of which five were immunoglobulins or apolipoproteins which would typically be removed via high-abundance protein depletion methods. Of the remaining seven proteins, two were of particular clinical interest: peptidase inhibitor 16 (PI16), and cystatin-C (CST3). PI16 and CST3 have both been implicated as markers for different disease states; loss of CST3 is used as a measure of kidney function in patients with CKD, although its expression can also be altered by other factors such as obesity,^59^ and PI16 has been implicated in diseases such as diabetes.^60^

A study by Chambers et al, 2013, also explored the effects of the “drying phase” on blood-derived biofluids, using Whatman903 filter papers for dried serum, plasma and blood samples, compared to “wet” controls. Chambers’ study did not identify any significant differences in the number of proteins identified between the dried and control conditions for any of the three biofluids, supporting the findings presented here.^61^

### Evaluation of High-Abundance Protein Depletion Techniques

Historically, exploration of the whole blood proteome has been hindered due to hemoglobin suppressing the signal from proteins of lower abundance.^35^ This has resulted in the identification of only limited portions of the whole blood proteome, with DDA-MS of DBS identifying protein numbers ranging from 120 to 350, and the high-abundance of hemoglobin being cited as the leading cause for low proteome coverage.^16,43,44^ Therefore, methods to remove hemoglobin from DBS are key to the adoption of DBS sampling. We therefore evaluated three affinity-based methods (NuGel-HemogloBind, HemogloBind, and HemoVoid) to deplete hemoglobin for improved DBS proteome coverage. Processing time for all three bead-based methods was similar, following very similar processes. However, while NuGel-HemogloBind and HemogloBind deplete hemoglobin directly by affinity binding, capturing the depleted proteome, the HemoVoid beads have very low affinity for hemoglobin, which instead act to capture and enrich lower abundant proteins. As shown in Figure 3A, this results in a statistically significant 49.7% increase in the number of proteins, and 65.4% increase in the number of peptides, identified using HemoVoid compared with undepleted blood (Supporting Data Sheet 5). The increasing numbers of proteins identified from the NuGel-HemogloBind (382 ± 10), HemogloBind (423 ± 5), and HemoVoid (545 ± 5) compared with undepleted sample (364 ± 5), inversely correlates with the amount of residual hemoglobin remaining after depletion (Figure 3B). This likely reflects a reduction in dynamic range and reduced ion suppression during peptide ionization following removal of highly abundant proteins from the matrix. Consistent with this observation, HemoVoid-treated samples exhibited the narrowest dynamic range of the four conditions, primarily due to a reduction in maximum detected intensity rather than a shift in the bulk intensity distribution, indicating more effective removal of high-abundance hemoglobin (Supporting Figure S9).

**Figure 3:**
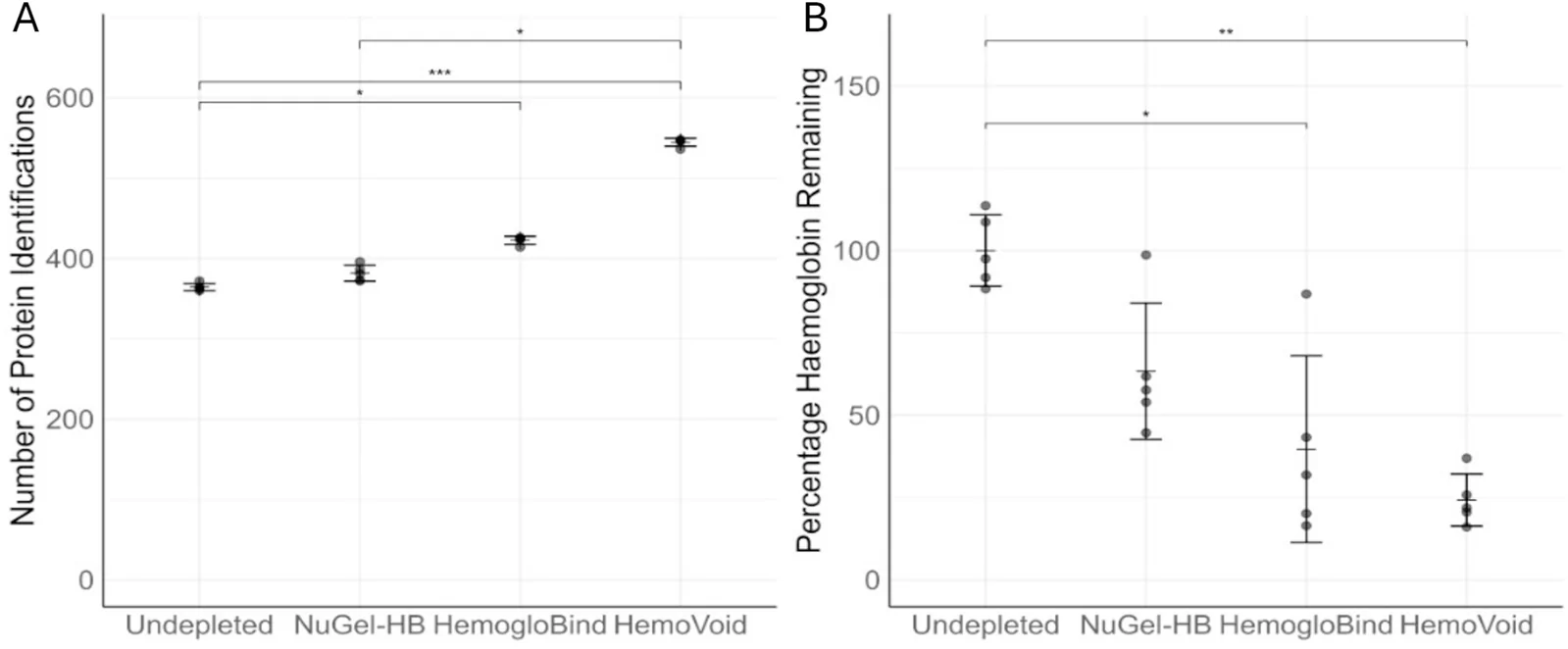
Comparison of hemoglobin and high-abundance protein depletion methods. (A) The average number of proteins identified in the dried blood spot proteome for each depletion method (*n* = 5). (B) The average percentage of hemoglobin depleted from DBS calculated based on the undepleted condition (*n* = 5). *= *p* < 0.05, **= *p* < 0.01, ***= *p* < 0.001, Error Bars= Standard Deviation.

Hemoglobin depletion efficiency differed substantially between methods. NuGel-HemogloBind was the least effective, retaining 63 ± 20.7% of the total signal relative to undepleted samples, whereas HemogloBind and HemoVoid retained 40 ± 28.4% and 24 ± 7.9% respectively (*p* = 5 × 10^–3^ compared to undepleted samples; Figure 3B). These values were calculated from the summed intensities of all hemoglobin proteins for each condition, expressed as a percentage of the corresponding undepleted samples. The same trend was observed at the peptide level, where the number of peptides and peptide spectral matches (PSMs) detected across all hemoglobin proteins and subunits decreased with increasing depletion efficiency; undepleted samples yielded 96 peptides (4463 PSMs), compared with only 42 peptides (1434 PSMs) following HemoVoid treatment. Complete peptide and PSM counts for all hemoglobin proteins and subunits are provided in Supporting Table S1, base peak chromatograms for three representative hemoglobin subunit peptides in Supporting Figures S10–S12.

As well as increasing protein identifications by 50% compared with undepleted material, HemoVoid also delivered the highest reproducibility, with 94.7% of proteins consistently identified across all five replicates (Supporting Figure S13). Quantitative reproducibility was similarly improved, as HemoVoid showed the lowest median abundance %CV at both the peptide (0.91%) and protein (0.58%) levels, outperforming NuGel-HemogloBind (peptide = 2.24%; protein = 1.47%), HemogloBind (peptide = 2.17%; protein = 1.13%), and undepleted samples (peptide = 1.38%; protein = 0.62%) (Supporting Figure S14). Although HemoVoid-treated samples showed marginally higher missingness than undepleted samples (4.23% vs 3.38%), this was considerably lower than NuGel-HemogloBind (5.67%) or HemogloBind (6.44%) (Supporting Figure S15).

Despite the overall improvement in proteome coverage, 25 proteins identified in the undepleted samples were not detected in the HemoVoid-treated samples (Supporting Figure S11 and Data Sheet 6). Gene ontology analysis of these proteins did not identify any significantly enriched terms. However, several were erythrocyte-associated intracellular proteins (HBE1, EPB42, PKM, SLC2A1), and cytoskeletal components (TUBB4A, TPM1, PFN1, LASP1), suggesting potential nonspecific codepletion of these protein classes during HemoVoid processing. Conversely, 115 proteins were uniquely identified in HemoVoid-treated samples. Although these proteins similarly failed to yield significantly enriched GO terms, they included multiple neutrophil granule-associated proteins, alongside complement and immunoglobin proteins, consistent with enhanced detection of lower-abundance blood proteins following hemoglobin depletion (Supporting Figure S16).

From a practical perspective, hemoglobin depletion increases per-sample processing costs, with consumable costs of approximately $26.15 per sample for HemoVoid, $36.90 for NuGel-HemogloBind, and $14.27 for HemogloBind. Automation was not evaluated in this study; however, NuGel-HemogloBind has been validated by the manufacturer for use with liquid-handling systems, which may support deployment in higher-throughput workflows.

### Cell-Free Dried Blood Spot Analysis

An alternative method to collect hemoglobin-free DBS is a cell-free collection device.^47^ These devices produce a “plasma like” sample by filtration, which is then dried in situ. In this study, Sep10 devices were used, which filter out both the red and white blood cells. Removal of cellular components during filtration has the added advantage of also removing red blood cell-containing hemoglobin. Performance of the Sep10 devices was compared with conventional DBS collected using b10 devices, and wet plasma samples. The DBS samples underwent HemoVoid depletion prior to analysis.

As expected, the presence of cellular material resulted in substantially higher numbers of proteins being identified in the DBS samples (386 ± 2, CV = 0.44%) compared with wet plasma (156 ± 1, CV = 0.45%, *p* = 1.1 × 10^–3^) (Figure 4A). In contrast, the number of proteins identified using the Sep10 devices (164 ± 4, CV = 2.18%) was not significantly different from those obtained with wet plasma samples (*p* = 0.15), although the Sep10 data exhibited greater variability, albeit within acceptable limits (<5%). Agreement between wet plasma and Sep10 samples was further supported by Bland-Altman analysis, which demonstrated minimal bias (−0.08 log_2_) and limits of agreement of – 1.86 to 1.71 log_2_ (Figure 4B). Notably, 95% of proteins fell within these limits, with only six proteins lying outside the expected range, indicating strong concordance between the two sample types (Figure 4B). Collectively, these results indicate that cell-free DBS collection using Sep10 offers a reliable alternative to conventional plasma sampling, while avoiding the need for additional hemoglobin depletion.

**Figure 4:**
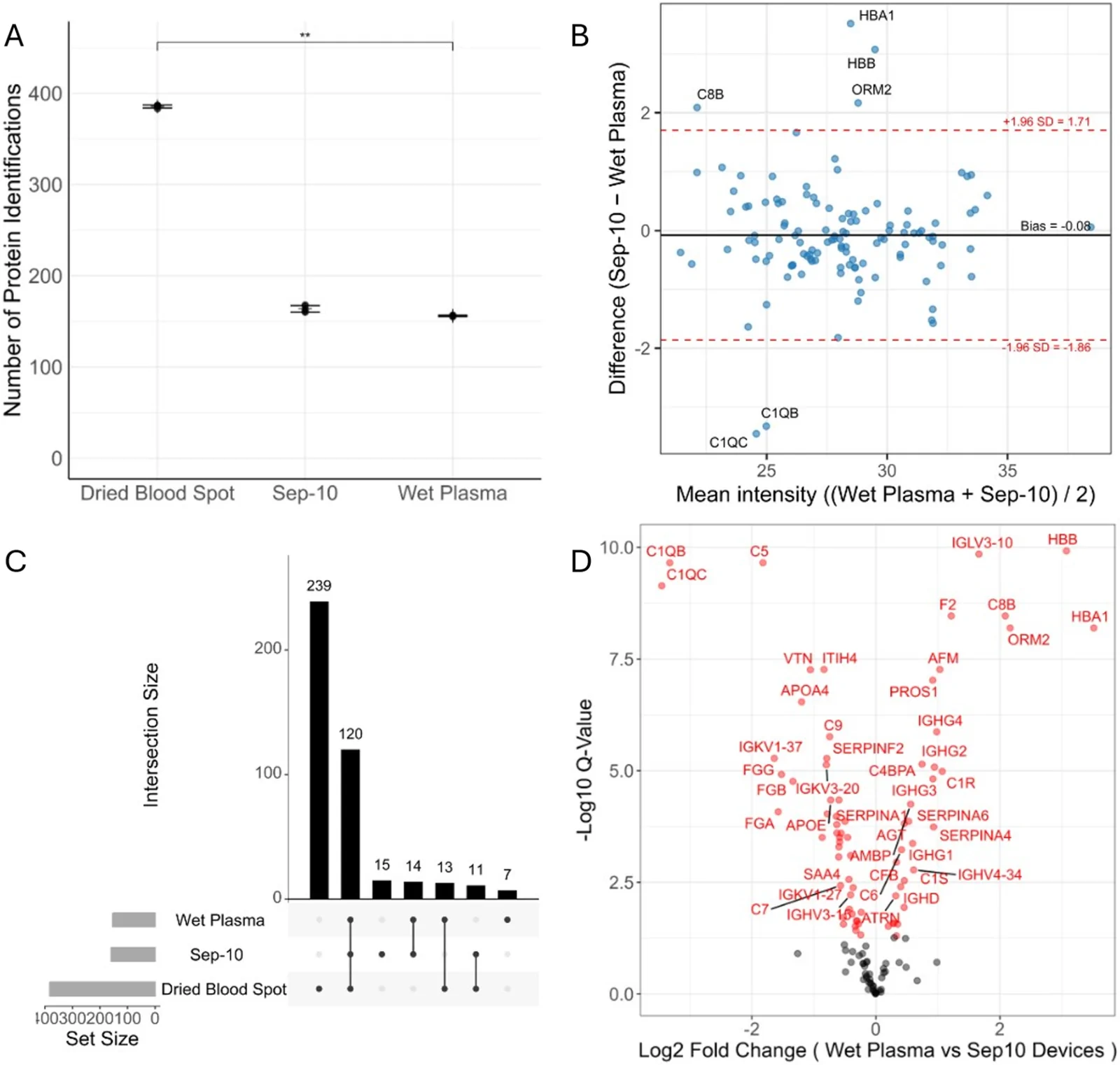
Analysis of cell-free DBS compared to traditional DBS and plasma samples. (A) The average number of proteins identified in each sample (*n* = 5). (B) Bland-Altman analysis comparing protein log2 protein intensity measurements between wet plasma and Sep10 samples. (C) Upset plot showing the cross over of proteins identified in each sample type (*n* = 5) (C) Volcano plot displaying log2mean fold change in protein abundance against −log 10, Q-values derived from 2-Sample significance tests (limma) comparing the identified proteomes from wet plasma (left) to Sep10 devices (right). **= *p* < 0.01, Error Bars = Standard Deviation. Protein names correlating to the Gene Name identifications can be found in the Supporting Information (Supporting Data Sheets 7 and 8).

Analysis of protein and peptide intensity distributions revealed trends consistent with those observed for wet plasma, and DBS samples described above. Wet plasma displayed the broadest dynamic range, followed by DBS and Sep-10. Interquartile range was near-identical across all three conditions, indicating that the majority of detected peptides occupied a comparable abundance window regardless of sample preparation method. The narrower dynamic range for Sep-10 samples was primarily driven by a reduction in low-abundance peptide detection, reflected by a higher minimum observed intensity (Supporting Figure S17).

Of the 383 proteins consistently identified using the b10 devices with HemoVoid depletion in this experiment, 120 were not identified using the Sep10 devices (Figure 4A,4C). This difference can be attributed to the acellular nature of the Sep10-derived matrix and resulting absence of cellular proteins from both the red and white blood cells, as previously discussed. A complete list of proteins unique to DBS samples is provided (Supporting Data Sheet 7).

No significant difference was observed in the overall number of proteins or peptides identified using the Sep10 devices compared with plasma (Supporting Figure S18). However, there was a statistically significant difference in the relative abundance of 73 proteins (of the 122 proteins identified in 75% of all samples) between these two methods (Figure 4D, and Supporting Data Sheet 8). Of these, 43/73 (59%) were higher in the plasma samples, and 30/73 (41%) in the Sep10 devices, with specific complement proteins and immunoglobulins being differentially abundant between the two sample preparation approaches. Notably, hemoglobin subunits HBA1 and HBB were both at significantly higher relative abundances is samples prepared using the Sep10 devices than in plasma, suggesting reduced efficiency of hemoglobin and red blood cell removal than traditional centrifugation methods. This may be due to partial erythrocyte lysis, shear-induced hemolysis, or incomplete removal of red blood cells during the Sep10 filtering process. Of the 73 proteins identified as significantly different in abundance between sample types, only 6 were outside the Bland-Altman limits of agreement discussed previously, indicating that the overall concordance between Sep10 and plasma samples remained high despite these differences. Additionally, protein-level missingness was marginally higher in Sep10 samples than wet plasma (5.86% vs 4.88%), although the opposite trend was observed at peptide level (2.52% vs 3.18%) (Supporting Figure S19). Together, these findings suggest that, while Sep10 processing introduces modest differences in the abundance of a subset of proteins, its overall analytical performance remains comparable to that of conventionally prepared plasma.

### Proof-of-Principle Clinical Validation in Chronic Kidney Disease

To demonstrate proof-of-principle for application of the developed HemoVoid method for proteome interrogation in a complex clinical disease state, we analyzed samples from 10 patients with CKD5 undergoing hemodialysis alongside 10 healthy controls (Table 1), compared with standard plasma. CKD patients were chosen due to the chronic nature of the disease, growing economic and health burden, and the need to preserve vein health, highlighting the value of alternative approaches such as capillary sampling.^15^ For this clinical proof-of-principle study, HemoVoid was selected as the method of choice given the familiarity of whole blood DBS use in other biobanks, such as the Danish Newborn Screening biobank.^62^

**Table 1:** Subject Demographic and Clinical Characteristics^a^

Demographics			
	Chronic Kidney Disease (Stage 5)	Healthy Control	Significance (*p* value)
Number of patients	10	10	
Age (years, median and SD)	73.5 ± 11.1	43 ± 7.9	4.5 × 10^–6^
Sex (% Male)	50%	60%	
Dialysis vintage (months and SD)	24.9 ± 28.6		
Primary renal disease (*n*)	IgA Nephropathy 2		
	Granulomatosis with Polyangiitis 1		
	Diabetic Nephropathy 3		
	Renovascular disease 1		
	Obstructive Uropathy 1		
	Unknown 2		
Laboratory values			
eGFRmL/min/1.73 m^2^ (median and SD)	5.5 ± 2	90 ± 6	4.2 × 10^–19^
C-reactive proteinmg/L (median and SD)	8.5 ± 7	1 ± 6	4.6 × 10^–2^

a Dialysis vintage refers to the months since dialysis treatment commenced. All patients were diagnosed with Stage 5 Chronic Kidney Disease (end stage kidney failure).

Using this approach, we identified 702 ± 16 proteins in the CKD5 cohort, compared with 685 ± 13 in the healthy control DBS samples. Of these, 31 were present at significantly different levels between the two groups (Figure 5A, and Supporting Data Sheet 9), with 23 (74%) elevated in the CKD5 group and 8 (26%) higher in the healthy control cohort. This included CRP, which is a routinely used clinical marker of inflammation.^63^ CRP was significantly elevated in the CKD group (*Q*-value = 3.9 × 10^–2^, log2FC = 3.45), consistent with expectations for the chronic disease state.

**Figure 5:**
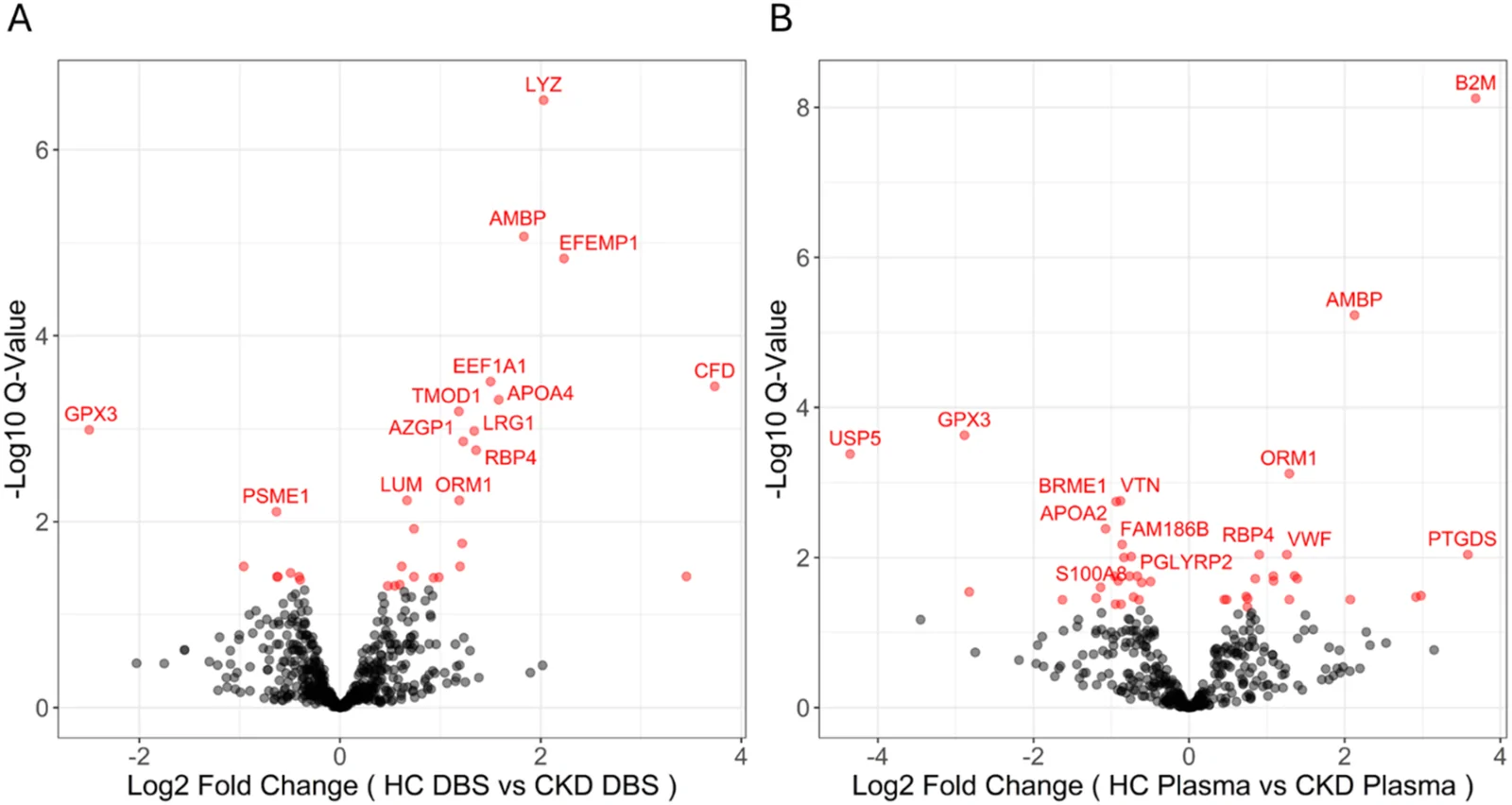
Quantitative profiling of dried blood spot proteomes. (A) Volcano plot displaying log2mean fold change in protein abundance against Q-values derived from 2-Sample significance tests (limma) comparing the identified proteomes from dried-blood spot (DBS) samples from healthy controls (HC) and CKD patients. Symbols corresponding to proteins with the greatest statistical confidence and highest fold change are labeled. (B) Volcano plot displaying log2mean fold change in protein abundance against *Q*-values derived from 2-Sample significance tests (limma) comparing the identified proteomes from plasma samples from healthy controls (HC) and CKD patients. Symbols corresponding to proteins with the greatest statistical confidence and highest fold change are labeled. (Protein names correlating to the Gene Name identifications can be found in the Supporting Information (Supporting Data Sheets 9 and 10)).

As expected, fewer proteins were identified from the CKD5 and healthy control plasma samples (474 ± 16 and 477 ± 14 respectively), compared with the DBS. Of these, 42 were significantly different between the two clinical cohorts (Figure 5B, Supporting Data Sheet 10), with 20 (48%) being elevated in CKD5, although this did not include CRP, which was not significantly different between the two cohorts (*Q*-value = 4.2 × 10^–1^). Interestingly, both hemoglobin subunits HBG1 and HBG2 were increased in CKD5 patients (HBG1 log2FC = 2.98, *Q*-value = 3.2 × 10^–2^; HBG2 log2FC = 2.92, *Q*-value = 3.4 × 10^–2^), presumably due to the complex biological changes seen in relation to CKD associated anemia, or as a consequence of the hemodialysis intervention on the blood.

Of the 73 proteins identified in total as significantly different between disease and control, 8 were identified in both the DBS and plasma samples (6 upregulated in CKD). Many of these have well-established roles in renal physiology and kidney disease. The upregulated proteins include: α-1-microglobulin (AMBP) (DBS log2FC = 1.83, *Q*-value = 8.6 × 10^–6^; Plasma log2FC = 2.13, *Q*-value = 5.9 × 10^–6^), Retinol Binding Protein 4 (RBP4) (DBS log2FC = 1.36, *Q*-value = 1.7 × 10^–3^; Plasma log2FC = 0.90, *Q*-value = 9.1 × 10^–3^), α-1-acid-glycoprotein (ORM1) (DBS log2FC = 1.19, *Q*-value = 5.9 × 10^–3^; Plasma log2FC = 1.29, *Q*-value = 7.6 × 10^–4^), Complement Factor D (CFD) (DBS log2FC = 3.74, *Q*-value = 3.5 × 10^–4^; Plasma log2FC= 2.07, *Q*-value = 3.6 × 10^–2^), Lysozyme C (LYZ) (DBS log2FC = 2.03, *Q*-value = 2.9 × 10^–7^; Plasma log2FC = 0.75, *Q*-value = 3.5 × 10^–2^), and Tropomodulin 1 (TMOD1) (DBS log2FC = 1.18, *Q*-value = 6.5 × 10^–4^; Plasma log2FC = 0.85, *Q*-value = 1.92 × 10^–2^).

AMBP is produced in the liver and adipose tissue and is known to degrade reactive oxygen species protecting tissues from damage. It is filtered in the glomeruli and degrades in the proximal tubular cells, where it can also be reabsorbed.^64^ Although the link between increased AMBP expression and CKD is not fully understood, it is theorized that increased oxidative stress in CKD upregulates AMBP, due to its antioxidative nature.^65^ It serves as a sensitive marker of tubular injury and oxidative stress in kidney disease.^66^ A similar hypothesis is used to explain ORM1 upregulation, which is an acute-phase protein, shown to increase in cases of inflammation, and known to protect against oxidative stress.^67^ It has been linked to renal injury, and is thought to correlate with disease severity.^68^ The precise role of RBP4, and how it may contribute to the pathophysiology of CKD are not clearly elucidated, however it is circulated by the kidneys and catabolised in the renal tubules, therefore reduction in kidney function is thought to lead to RBP4 accumulation and build up within the blood.^69^ Elevated levels of circulated or urinary RBP4 are therefore proposed to correlate with reduced glomerular filtration rate, tubular disfunction, and CKD progression.^70^ TMOD1 is an actin capping protein and assists in maintaining the structure of the distal tubules of the kidney, therefore helping to regulate water homeostasis. Disruption to healthy TMOD1 levels could therefore indicate issues with kidney function, due to its integral role.^71^ Additionally, LYZ and CFD are both involved in the innate immune response, activation of which is heavily associated with CKD pathogenesis and progression.^72–74^

Alternatively, the two proteins shown to be downregulated in both the DBS and plasma samples are both known to protect against oxidative stress. ATP-citrate lyase (ACLY) (DBS log2FC = −0.40, *Q*-value= 4.22 × 10^–2^; Plasma log2FC = −0.91, *Q*-value = 2.0 × 10^–2^), and Glutathione peroxidase 3 (GPX3) (DBS log2FC = −2.50, *Q*-value = 1.0 × 10^–3^; Plasma log2FC = −2.89, *Q*-value = 2.3 × 10^–4^). ACLY is often seen to be downregulated in chronic diseases such as CKD due to metabolic alterations, such as the Warburg effect, leading to dyslipidaemia, and as a result lower levels of ACLY.^75^ The reduction in GPX3 levels, however, is likely due to imbalance between its production and usage rate. GPX3 is produced in the kidneys, a process which slows with kidney dysfunction (as is seen in CKD), leading to comparatively decreased levels. Additionally, GPX3 catalyzes the reduction of reactive oxygen species, a process often referred to as scavenging. As a result, GPX3 can be transiently oxidized, meaning that at any given time point there may be variable levels of GPX3 identifiable.^76^

Complement proteins were also observed to be elevated in CKD, irrespective of sampling strategy, however the specific complement proteins showing significance varied dependent on sampling type. The plasma samples showed significant elevation of C2 (*Q*-value = 3.6 × 10^–2^), C4BPB (*Q*-value = 1.8 × 10^–2^), C5 (*Q*-value = 3.6 × 10^–2^), CFD (*Q*-value = 3.6 × 10^–2^), and CFI (*Q*-value = 3.3 × 10^–2^), while DBS showed significant elevation of CFD (*Q*-value = 3.5 × 10^–4^), and CFHR2 (*Q*-value = 4.0 × 10^–2^). The complement system has established roles in immune mediated kidney diseases, such as lupus nephritis, atypical hemolytic uremic syndrome and C3 glomerulopathy, which can all lead to the development of CKD, however the role of complement in CKD5 is not clearly elucidated.^77,78^

Additionally, there were 23 proteins only identified as significantly different between the healthy control and CKD cohorts using the DBS samples, six of which have been shown to directly link to CKD and renal function (including CRP, which has been previously discussed): Fibulin 3 (EFEMP1) (log2FC= 2.23, *Q*-value = 1.5 × 10^–5^), Leucine-rich α-2-glycoprotein 1 (LRG1) (log2FC = 1.34, *Q*-value = 1.0 × 10^–3^), Lumican (LUM) (log2FC = 0.67, *Q*-value = 5.9 × 10^–3^), Defensin α 1 (DEFA1) (log2FC = 0.60, *Q*-value = 4.8 × 10^–2^), and Apolipoprotein A-IV (APOA4) (log2FC = 1.58, *Q*-value = 4.9 × 10^–4^).

EFEMP1 is an extracellular matrix glycoprotein involved in tissue structural organization and remodeling and is associated with declining kidney function. It is hypothesized to contribute to the progression of renal impairment.^79^ Similarly, LRG1 is a multifunctional signaling protein implicated in fibrosis and angiogenesis. It is consistently upregulated across various kidney diseases, where it contributes to pathological TGF-β signaling and progression of renal fibrosis.^80^ LUM represents another extracellular matrix-associated protein, which is known to be upregulated in diabetic nephropathy and to correlate inversely with estimated glomerular filtration rate (eGFR).^81^ Additionally, DEFA1, is an antimicrobial protein involved in innate immune defense within the kidney. It contributes to host protection against infection, while also modulating inflammatory responses.^82^ Finally, APOA4 is an apolipoprotein involved in lipid metabolism, that has been identified as a circulating biomarker predictive of diabetic kidney disease progression. Its levels are associated with metabolic dysfunction and reported to reflect early signs of kidney disease progression.^83^ Annotated fragmentation spectra for a representative peptide for each protein discussed can be found in the Supporting Data (Supporting Figures S20–S44).

Overall, the proof-of-principle study demonstrated strong concordance between DBS- and plasma-derived proteomes. Importantly, all proteins identified as significantly differentially abundant in both sample types exhibited consistent direction of change, supporting their potential utility as CKD-associated biomarkers. Although differences were observed in the magnitude of some abundance changes between DBS and plasma, these did not alter the overall biological interpretation of the findings. A limitation of this pilot cohort was the inability to adequately age-match participants across study groups (Table 1). Age-adjusted analyses were not feasible as aging is intrinsically linked to CKD progression, making it difficult to disentangle the individual contributions of these factors within a small cohort. However, as the primary aim of this analysis was to establish proof-of-principle concordance between plasma and DBS proteomes using matched samples from the same patients, this potential confound affected both sample types equally and therefore is unlikely to have a substantive effect of the validity of the comparative analysis. Future studies would benefit from larger, prospectively age-matched cohorts to increase statistical power and improve confidence in the identification of CKD-associated protein signatures. In addition, the application of data-independent acquisition (DIA) workflows could also serve to enhance biomarker discovery by providing more comprehensive and reproducible protein quantification than data-dependent acquisition (DDA), increasing sensitivity for detecting differentially abundant proteins in larger-scale validation studies.

## Conclusions

In this study, we establish and validate an optimized workflow for LC–MS/MS-based proteomic analysis of dried blood spots (DBS), with a particular focus on improving proteome depth through hemoglobin depletion and evaluation of a novel cell-free DBS device. Systematic evaluation of reconstitution buffers and DBS devices demonstrated that 50 mM ammonium bicarbonate provided optimal and reproducible protein recovery. Across the evaluated workflows, depletion using HemoVoid resulted in the greatest performance, increasing protein identifications from 364 ± 5 in undepleted samples to 545 ± 5, alongside improved reproducibility, with 94.7% of proteins observed across all replicates. This improvement is consistent with the reduction in hemoglobin content to 24 ± 7.9% of the undepleted level, supporting the importance of dynamic range reduction for enhanced proteome coverage.

Comparison of DBS with matched plasma and dried plasma samples confirmed that DBS provides substantially greater proteome coverage, with 573 ± 2 proteins identified compared to 181 ± 3 and 209 ± 3 for wet and dried plasma, respectively. This increase reflects the contribution of cellular proteins, supported by gene ontology analysis indicating enrichment of cytosolic components. Despite these compositional differences, drying itself introduced minimal bias, with no significant difference between wet and dried plasma proteomes.

Evaluation of cell-free DBS devices demonstrated that plasma-like samples can be obtained without postcollection depletion, being comparable in terms of proteome coverage to wet plasma, but lower with respect to conventional DBS given the absence of cellular material. These findings highlight a trade-off between analytical depth and workflow simplicity. Cell-free devices reduce processing time and cost, with an approximately 4-fold cost saving compared to depletion-based approaches and are therefore well suited to large-scale studies where throughput and cost efficiency are prioritized. In contrast, conventional DBS combined with hemoglobin depletion provides substantially greater proteome coverage and is more appropriate for discovery-driven applications where detection of lower-abundance or intracellular proteins is required.

Application of the optimized workflow to a cohort of patients with stage 5 CKD identified 31 differentially abundant proteins compared with matched controls. Clinically relevant markers such as C-reactive protein were significantly elevated in DBS from patients, demonstrating that DBS sampling captures known disease-associated biology. Plasma analysis yielded fewer protein identifications overall (∼30%) than DBS, with a partially overlapping set of significant proteins being identified, demonstrating the same trends in differential abundance. These results demonstrate that DBS and plasma provide complementary views of disease biology, with DBS offering additional sensitivity to whole-blood derived changes. While we have demonstrated proof-of-principle here with DDA, proteome coverage will undoubtedly be improved in future investigations using DIA workflows.

From a practical perspective, DBS sampling offers patients clear benefits as an adjust to traditionally collected samples for biobanking, including reduced invasiveness, preservation of venous access, and the ability to perform remote and longitudinal sampling. These advantages are particularly important in chronic conditions such as chronic kidney disease, where repeated sampling is required, or rare diseases which may require global participation. The choice of workflow should therefore be guided by both analytical and practical considerations. Depletion-based DBS workflows are advantageous when maximal proteome depth is required, for example in biomarker discovery or mechanistic studies. In contrast, cell-free DBS approaches are advantageous in population-scale or longitudinal studies where reduced cost, simplified processing, and patient convenience are prioritized. Moreover, the methods developed in this study are species independent and could therefore be used for a range of preclinical or veterinary research applications.

Collectively, this work demonstrates that DBS sampling, when combined with optimized processing, provides a robust and flexible platform for routine clinical proteomics. The ability to tailor workflows according to analytical depth, cost, and patient burden supports broader application across diverse study designs and clinical contexts and facilitates expanded access to large and geographically dispersed cohorts.

## Supplementary Material





## Data Availability

PRIDE All MS data has been deposited at the ProteomeXchange Consortium (http://proteomecentral.proteomexchange.org) via the PRIDE partner repository with the data set identifier PXD079508.

## Notes

Figure S1: Optimization of sample reconstitution buffer. The average number of peptides identified with each reconstitution buffer for either the (A) Capitainer b10 DBS, (B) Neoteryx VAMS Mitra DBS, or (C) Whatman903 DBS devices (*n* = 5). *= *p* < 0.05, **= *p* < 0.01, ***= *p* < 0.001, error bars = standard deviation. AmBic= Ammonium bicarbonate, PBS = Phosphate buffered saline, TFA = Trifluoroacetic acid, TFE = Tetrafluoroethylene; Figure S2: Optimization of sample reconstitution buffer. Bar chart showing the reproducibility of protein identifications across *n* = 5 replicates with each reconstitution buffer for either the (A) Capitainer b10, (B) Neoteryx VAMs, or (C) Whatman903 devices; Figure S3: Optimization of sample reconstitution buffer. (A) Box plot showing the percentage coefficient of variance of protein relative abundance using each reconstitution buffer with Capitainer b10 devices (*n* = 5). (B) Box plot showing the percentage coefficient of variance of protein relative abundance using each reconstitution buffer with Neoteryx VAMs devices (*n* = 5). (C) Box plot showing the percentage coefficient of variance of protein relative abundance using each reconstitution buffer with Whatman903 devices (*n* = 5). Figure S4: Optimization of sample reconstitution buffer. (A) Box plot showing the percentage coefficient of variance of peptide relative abundance using each reconstitution buffer with Capitainer b10 devices (*n* = 5). (B) Box plot showing the percentage coefficient of variance of peptide relative abundance using each reconstitution buffer with Neoteryx VAMs devices (*n* = 5). (C) Box plot showing the percentage coefficient of variance of peptide relative abundance using each reconstitution buffer with Whatman903 devices (*n* = 5). Figure S5: Optimization of sample reconstitution buffer. (A) Bar chart showing the percentage of missing peptide values within each condition using each reconstitution buffer with Capitainer b10 devices (*n* = 5). (B) Bar chart showing the percentage of missing peptide values within each condition using each reconstitution buffer with Neoteryx VAMs devices (*n* = 5). (C) Bar chart showing the percentage of missing peptide values within each condition using each reconstitution buffer with Whatman903 devices (*n* = 5). Figure S6: Optimization of sample reconstitution buffer. (A) Bar chart showing the percentage of missing protein values within each condition using each reconstitution buffer with Capitainer b10 devices (*n* = 5). (B) Bar chart showing the percentage of missing protein values within each condition using each reconstitution buffer with Neoteryx VAMs devices (*n* = 5). (C) Bar chart showing the percentage of missing protein values within each condition using each reconstitution buffer with Whatman903 devices (*n* = 5). Figure S7: Comparison of plasma, dried plasma and dried blood. (A) The average number of peptides identified in dried blood spot, dried plasma spot, and wet plasma samples (*n* = 5). (B) The number of peptides identified oer protein for each condition **= *p* < 0.01. Figure S8: Peptide- and protein-level intensity distributions across sample types. Violin plots showing the distribution of log2-transformed (A) peptide and (B) protein intensity values for dried blood spot, dried plasma spot, and wet plasma samples. White boxplots indicate the median and interquartile range (IQR) within each violin. Figure S9: Peptide- and protein-level intensity distributions across sample types. Violin plots showing the distribution of log2-transformed (A) peptide and (B) protein intensity values for each depletion method. White boxplots indicate the median and interquartile range (IQR) within each violin. Table S1: Evaluation of high abundance protein depletion techniques. Table showing the number of Hemoglobin Subunit proteins, peptides, peptide spectral matches (PSMs) and unique peptides identified using each depletion method (*n* = 5). Figure S10: Base peak chromatogram demonstrating reduction of hemoglobin-derived peptide signal following HemoVoid depletion. Overlaid LC-MS/MS chromatographic traces show relative abundance for an undepleted plasma sample (orange) and a HemoVoid depleted sample (purple). The peak highlighted corresponds to the peptide MFLSFPTTK, derived from hemoglobin subunit α 1 (HBA1), with a monoisotopic mass of 536.281 Da. Peak area values for this hemoglobin peptide were reduced following HemoVoid treatment (HV = 1.55 × 10^9^) compared to the undepleted sample (UD = 5.89 × 10^9^). UD= Undepleted. HV= HemoVoid. Figure S11: Base peak chromatogram demonstrating reduction of hemoglobin-derived peptide signal following HemoVoid depletion. Overlaid LC-MS/MS chromatographic traces show relative abundance for an undepleted plasma sample (orange) and a HemoVoid depleted sample (purple). The peak highlighted corresponds to the peptide SAVTALWGK, derived from hemoglobin subunit β (HBB), with a monoisotopic mass of 466.764 Da. Peak area values for this hemoglobin peptide were reduced following HemoVoid treatment (HV = 1.10 × 10^9^) compared to the undepleted sample (UD = 4.69 × 10^9^). UD= Undepleted. HV= HemoVoid. Figure S12: Base peak chromatogram demonstrating reduction of hemoglobin-derived peptide signal following HemoVoid depletion. Overlaid LC-MS/MS chromatographic traces show relative abundance for an undepleted plasma sample (orange) and a HemoVoid depleted sample (purple). The peak highlighted corresponds to the peptide LLVYPWTQR, derived from hemoglobin subunit delta (HBD), with a monoisotopic mass of 637.866 Da. Peak area values for this hemoglobin peptide were reduced following HemoVoid treatment (HV = 1.64 × 10^9^) compared to the undepleted sample (UD = 5.54 × 10^9^). UD = Undepleted. HV = HemoVoid. Figure S13: Evaluation of High Abundance Protein Depletion Techniques. Bar chart showing the reproducibility of protein identifications using each depletion method (*n* = 5). Figure S14: Evaluation of High Abundance Protein Depletion Techniques. Box plot showing the percentage coefficient of variance of (A) peptide and (B) protein relative abundance using each depletion method (*n* = 5). Figure S15: Evaluation of High Abundance Protein Depletion Techniques. (A) Bar chart showing the percentage of missing protein values within each condition using each depletion method (*n* = 5). (B) Bar chart showing the percentage of missing protein values within each condition using each depletion method (*n* = 5). Figure S16: Evaluation of High Abundance Protein Depletion Techniques. Upset plot showing the cross over of proteins identified using each depletion method (*n* = 5). Figure S17: Peptide- and protein-level intensity distributions across sample types. Violin plots showing the distribution of log2-transformed (A) peptide and (B) protein intensity values for each depletion method. White boxplots indicate the median and interquartile range (IQR) within each violin. Figure S18: Analysis of cell-free DBS compared to traditional DBS and plasma samples. The average number of peptides identified in dried blood spot, dried plasma spot, and wet plasma samples (*n* = 5). **= *p* < 0.01. Figure S19: Analysis of cell-free DBS compared to traditional DBS and plasma samples. (A) Bar chart showing the percentage of missing protein values within each condition using each sample type (*n* = 5). (B) Bar chart showing the percentage of missing protein values within each condition using each sample type (*n* = 5). Figure S20: Annotated MS/MS fragmentation spectrum for an identified peptide from C-Reactive Protein in dried blood spots. Figure S21: Annotated MS/MS fragmentation spectrum for an identified peptide from Protein AMBP in dried blood spots. Figure S22: Annotated MS/MS fragmentation spectrum for an identified peptide from Retinol Binding Protein 4 in dried blood spots. Figure S23: Annotated MS/MS fragmentation spectrum for an identified peptide from α-1-acid glycoprotein 1in dried blood spots. Figure S24: Annotated MS/MS fragmentation spectrum for an identified peptide from complement factor D in dried blood spots. Figure S25: Annotated MS/MS fragmentation spectrum for an identified peptide from lysozyme C in dried blood spots. Figure S26: Annotated MS/MS fragmentation spectrum for an identified peptide from tropomodulin 1 in dried blood spots. Figure S27: Annotated MS/MS fragmentation spectrum for an identified peptide from ATP citrate lyase in dried blood spots. Figure S28- Annotated MS/MS fragmentation spectrum for an identified peptide from glutathione peroxidase 3 in dried blood spots. Figure S29: Annotated MS/MS fragmentation spectrum for an identified peptide from EGF-containing fibulin-like extracellular matrix protein 1 in dried blood spots. Figure S30: Annotated MS/MS fragmentation spectrum for an identified peptide from leucine-rich α-2-glycoprotein 1 in dried blood spots. Figure S31: Annotated MS/MS fragmentation spectrum for an identified peptide from lumican in dried blood spots. Figure S32: Annotated MS/MS fragmentation spectrum for an identified peptide from defensin α 1 in dried blood spots. Figure S33: Annotated MS/MS fragmentation spectrum for an identified peptide from apolipoprotein A4 in dried blood spots. Figure S34: Annotated MS/MS fragmentation spectrum for an identified peptide from complement factor h related protein 2 in dried blood spots. Figure S35: Annotated MS/MS fragmentation spectrum for an identified peptide from protein AMBP in plasma samples. Figure S36: Annotated MS/MS fragmentation spectrum for an identified peptide from retinol binding protein 4 in plasma samples. Figure S37: Annotated MS/MS fragmentation spectrum for an identified peptide from α 1 acid glycoprotein 1 in plasma samples. Figure S38: Annotated MS/MS fragmentation spectrum for an identified peptide from complement factor D in plasma samples. Figure S39: Annotated MS/MS fragmentation spectrum for an identified peptide from lysozyme C in plasma samples. Figure S40: Annotated MS/MS fragmentation spectrum for an identified peptide from glutathione peroxidase 3 in plasma samples. Figure S41: Annotated MS/MS fragmentation spectrum for an identified peptide from complement 2 in plasma samples. Figure S42: Annotated MS/MS fragmentation spectrum for an identified peptide from complement 4 binding protein in plasma samples. Figure S43: Annotated MS/MS fragmentation spectrum for an identified peptide from complement 5 in plasma samples. Figure S44: Annotated MS/MS fragmentation spectrum for an identified peptide from complement factor I in plasma samples (PDF)
Sheet S1: Contents; Sheet S2: Blanks proteins-comparison of micro-sampling devices; Sheet S3: Dry blood unique proteins-comparison of plasma, dried plasma, and dried blood; Sheet S4: Wet plasma unique proteins-comparison of plasma, dried plasma, and dried blood; Sheet S5: HemoVoid unique proteins-evaluation of high abundance protein depletion techniques; Sheet S6: Undepleted unique proteins-evaluation of high abundance protein depletion techniques; Sheet S7: DBS unique proteins (CF)-cell free dried blood spot analysis; Sheet S8: Significant (PlasmaSep10)-cell free dried blood spot analysis; Sheet S9: Significant (CKD DBS)-proof-of-principle clinical validation in chronic kidney disease; Sheet S10: Significant (CKD plasma)-proof-of-principle clinical validation in chronic kidney disease (XLSX)

## References

[1] Kuzmanov U., Emili A. (2013). Protein-protein interaction networks: probing disease mechanisms
using model systems. Genome Med..

[2] Bergendahl L. T., Gerasimavicius L., Miles J., Macdonald L., Wells J. N., Welburn J. P. I., Marsh J. A. (2019). The role of protein
complexes in human genetic disease. Protein
Sci..

[3] Ignjatovic V., Geyer P. E., Palaniappan K. K., Chaaban J. E., Omenn G. S., Baker M. S. (2019). Mass Spectrometry-Based Plasma Proteomics:
Considerations from Sample Collection to Achieving Translational Data. J. Proteome Res..

[4] Demir S., Yet I., Sardan Ekiz M., Sag E., Bilginer Y., Celikbicak O. (2023). Plasma Proteomic Analysis
Reveals the Potential Role of Lectin and
Alternative Complement Pathways in IgA Vasculitis Pathogenesis. Diagnostics.

[5] Davies E., Chetwynd A., Mcdowell G., Rao A., Oni L. (2024). The current
use of proteomics and metabolomics in glomerulonephritis: a systematic
literature review. J. Nephrol..

[6] Palstrøm N. B., Matthiesen R., Rasmussen L. M., Beck H. C. (2022). Recent Developments
in Clinical Plasma Proteomics—Applied to Cardiovascular Research. Biomedicines.

[7] Hällqvist J., Bartl M., Dakna M., Schade S., Garagnani P., Bacalini M.-G. (2024). Plasma proteomics identify
biomarkers predicting
Parkinson’s disease up to 7 years before symptom onset. Nat. Commun..

[8] Geyer P. E., Voytik E., Treit P. V., Doll S., Kleinhempel A., Niu L. (2019). Plasma Proteome Profiling to detect and avoid sample-related
biases in biomarker studies. EMBO Mol. Med..

[9] Ashworth M., Small B., Oldfield L., Evans A., Greenhalf W., Halloran C., Costello E. (2021). The holding
temperature of blood
during a delay to processing can affect serum and plasma protein measurements. Sci. Rep..

[10] Chapman K., Chivers S., Gliddon D., Mitchell D., Robinson S., Sangster T. (2014). Overcoming the barriers to the uptake of nonclinical
microsampling in regulatory safety studies. Drug Discovery Today.

[11] Campbell A. J., Palstrøm N. B., Rasmussen L. M., Lindholt J. S., Beck H. C. (2025). From blood
drops to biomarkers: a scoping review of microsampling in mass spectrometry-based
proteomics. Clin. Proteomics.

[12] Lamond M. K., Chetwynd A. J., Salama A. D., Oni L. (2024). A Systematic
Literature
Review on the Use of Dried Biofluid Microsampling in Patients With
Kidney Disease. J. Clin. Lab. Anal..

[13] Howie S. R. (2011). Blood sample
volumes in child health research: review of safe limits. Bull. World Health Org..

[14] Chetwynd A. J., Marro J., Whitty L., Ainsworth S., Preston J., Salama A., Oni L. (2022). Perceptions
and acceptability
of microsampling in children and young people: a single-centre survey. BMJ Paediatrics Open.

[15] Marsh, A. M. ; Genova, R. ; Buicko-Lopez, J. L. Dialysis Fistula: StatPearls. 2023; Available from: https://www.ncbi.nlm.nih.gov/books/NBK559085/. Updated May 23, 2023. 32644511

[16] Rosting C., Yu J., Cooper H. J. (2018). High Field
Asymmetric Waveform Ion Mobility Spectrometry
in Nontargeted Bottom-up Proteomics of Dried Blood Spots. J. Proteome Res..

[17] Palmer E. A., Cooper H. J., Dunn W. B. (2019). Investigation
of the 12-Month Stability
of Dried Blood and Urine Spots Applying Untargeted UHPLC-MS Metabolomic
Assays. Anal. Chem..

[18] Tuaillon E., Kania D., Pisoni A., Bollore K., Taieb F., Ontsira Ngoyi E. N. (2020). Dried Blood Spot Tests for the Diagnosis
and Therapeutic Monitoring of HIV and Viral Hepatitis B and C. Front. Microbiol..

[19] Zailani N. N. B., Ho PC-L. (2023). Dried Blood Spots—A
Platform for Therapeutic
Drug Monitoring (TDM) and Drug/Disease Response Monitoring (DRM). Eur. J. Drug Metab. Pharmacokinet..

[20] Lehmann S., Delaby C., Vialaret J., Ducos J., Hirtz C. (2013). Current and
future use of “dried blood spot” analyses in clinical
chemistry. Clin. Chem. Lab. Med..

[21] Jacobson T. A., Rahbari K. J., Schwartz W. A., Bae Y., Zhang R., Nunes D. A. (2025). Dried Blood Spots to Assess Cardiovascular-Kidney-Metabolic
Health. J. Am. Heart Assoc..

[22] Velghe S., Stove C. P. (2018). Evaluation of the Capitainer-B Microfluidic Device
as a New Hematocrit-Independent Alternative for Dried Blood Spot Collection. Anal. Chem..

[23] Cvetko A., Tijardović M., Bilandžija-Kuš I., Gornik O. (2022). Comparison of self-sampling
blood collection for N-glycosylation
analysis. BMC Res. Notes.

[24] Patel A. J., Wesley R., Leitman S. F., Bryant B. J. (2013). Capillary
versus
venous haemoglobin determination in the assessment of healthy blood
donors. Vox Sang..

[25] Kocur A., Pawiński T. (2023). Volumetric
Absorptive Microsampling in Therapeutic
Drug Monitoring of Immunosuppressive Drugs—From Sampling and
Analytical Issues to Clinical Application. Int.
J. Mol. Sci..

[26] Kang M., Yoo S., Jung Y., Lim H., Lee M. H., Ryu J. K., Lee J. I. (2024). Factors affecting peripheral neuropathy induced by
nanoparticle albumin-bound paclitaxel in patients with pancreatic
cancer. Br. J. Clin. Pharmacol..

[27] Pigliasco F., Cafaro A., Barco S., Biondi M., Stella M., Mattioli F. (2024). A VAMS-based LC–MS/MS method for precise
cenobamate quantification in epilepsy (patients). Epilepsia Open.

[28] Leino A. D., Takyi-Williams J., Takyi-Williams J., Park J. M., Norman S. P., Sun D., Farris K. B. (2024). Clinical validation of two volumetric absorptive microsampling
devices to support home-based therapeutic drug monitoring of immunosuppression. Br. J. Clin. Pharmacol..

[29] Whelan S. A., Hendricks N., Dwight Z. L., Fu Q., Moradian A., Van Eyk J. E., Mockus S. M. (2023). Assessment of a 60-biomarker health
surveillance panel (HSP) on whole blood from remote sampling devices
by targeted LC/MRM-MS and discovery DIA-MS analysis. Anal. Chem..

[30] Fredolini C., Dodig-Crnković T., Bendes A., Dahl L., Dale M., Albrecht V. (2024). Proteome profiling of
home-sampled dried blood spots reveals proteins of SARS-CoV-2 infections. Commun. Med..

[31] Couacault P., Avella D., Londoño-Osorio S., Lorenzo A. S., Gradillas A., Kärkkäinen O. (2024). Targeted
and untargeted metabolomics and lipidomics in dried blood microsampling:
Recent applications and perspectives. Anal.
Sci. Adv..

[32] Thangavelu M. U., Wouters B., Kindt A., Reiss I. K. M., Hankemeier T. (2023). Blood microsampling
technologies: Innovations and applications in 2022. Anal. Sci. Adv..

[33] Volani C., Malfertheiner C., Caprioli G., Fjelstrup S., Pramstaller P. P., Rainer J., Paglia G. (2023). VAMS-Based Blood Capillary
Sampling for Mass Spectrometry-Based Human Metabolomics Studies. Metabolites.

[34] Shen X., Kellogg R., Panyard D. J., Bararpour N., Castillo K. E., Lee-Mcmullen B. (2024). Multi-omics microsampling
for the profiling of lifestyle-associated changes in health. Nat. Biomed. Eng..

[35] Boschetti E., Righetti P. G. (2023). Low-Abundance Protein
Enrichment for Medical Applications:
The Involvement of Combinatorial Peptide Library Technique. Int. J. Mol. Sci..

[36] Anderson N. L., Anderson N. G. (2002). The Human Plasma
Proteome. Mol.
Cell. Proteomics.

[37] Mathew, J. ; Sankar, P. ; Varacallo, M. Physiology, Blood Plasma: StatPearls. 2023; Available from: https://www.ncbi.nlm.nih.gov/books/NBK531504/. Updated April 24, 2024.

[38] Annesley T.
M. (2003). Ion Suppression
in Mass Spectrometry. Clin. Chem..

[39] Malsagova K., Kopylov A., Stepanov A., Butkova T., Izotov A., Kaysheva A. (2020). Dried Blood
Spot in Laboratory: Directions and Prospects. Diagnostics.

[40] Altıntas E. B., Türkmen D., Karakoç V., Denizli A. (2011). Hemoglobin binding
from human blood hemolysate with poly­(glycidyl methacrylate) beads. Colloids Surf., B.

[41] Williams L. M., Fu Z., Dulloor P., Yen T., Barron-Casella E., Savage W. (2010). Hemoglobin
depletion from plasma: Considerations for
proteomic discovery in Sickle Cell disease and other hemolytic processes. PROTEOMICS–Clin. Appl..

[42] Walpurgis K., Kohler M., Thomas A., Wenzel F., Geyer H., Schänzer W., Thevis M. (2012). Validated hemoglobin-depletion approach
for red blood cell lysate proteome analysis by means of 2D PAGE and
Orbitrap MS. Electrophoresis.

[43] Molloy M. P., Hill C., O’Rourke M. B., Chandra J., Steffen P., McKay M. J. (2022). Proteomic
Analysis of Whole Blood Using Volumetric
Absorptive Microsampling for Precision Medicine Biomarker Studies. J. Proteome Res..

[44] Martin N. J., Bunch J., Cooper H. J. (2013). Dried Blood
Spot Proteomics: Surface
Extraction of Endogenous Proteins Coupled with Automated Sample Preparation
and Mass Spectrometry Analysis. J. Am. Soc.
Mass Spectrom..

[45] Mcdowell C. T., Weaver A. L., Vargas-Cruz N., Kaiser N. K., Nichols C. M., Pestano G. A. (2024). Use of a Novel Whole
Blood Separation and Transport
Device for Targeted and Untargeted Proteomics. Biomedicines.

[46] Velásquez-Orozco F., Rando-Segura A., Martínez-Camprecios J., Salmeron P., Najarro-Centeno A., Esteban À. (2021). Utility of the Cobas
Plasma Separation Card as a Sample Collection Device for Serological
and Virological Diagnosis of Hepatitis C Virus Infection. Diagnostics.

[47] Yang X., Forouzan O., Brown T. P., Shevkoplyas S. S. (2012). Integrated
separation of blood plasma from whole blood for microfluidic paper-based
analytical devices. Lab Chip.

[48] Mielczarek W. S., Obaje E. A., Bachmann T. T., Kersaudy-Kerhoas M. (2016). Microfluidic
blood plasma separation for medical diagnostics: is it worth it?. Lab Chip.

[49] Hauser J., Lenk G., Ullah S., Beck O., Stemme G., Roxhed N. (2019). An Autonomous Microfluidic
Device for Generating Volume-Defined
Dried Plasma Spots. Anal. Chem..

[50] Baillargeon K. R., Murray L. P., Deraney R. N., Mace C. R. (2020). High-Yielding Separation
and Collection of Plasma from Whole Blood Using Passive Filtration. Anal. Chem..

[51] Baillargeon K. R., Morbioli G. G., Brooks J. C., Miljanic P. R., Mace C. R. (2022). Direct
Processing and Storage of Cell-Free Plasma Using Dried Plasma Spot
Cards. ACS Meas. Sci. Au.

[52] Grüner N., Stambouli O., Ross R. S. (2015). Dried Blood Spots - Preparing and
Processing for Use in Immunoassays and in Molecular Techniques. J. Visualized Exp..

[53] Samenuk G. M., Kelley A. R., Perry G., Bach S. B. H. (2019). Rapid method
towards proteomic analysis of dried blood spots by MALDI mass spectrometry. Clin. Mass Spectrom..

[54] Nakajima D., Kawashima Y., Shibata H., Yasumi T., Isa M., Izawa K. (2020). Simple and Sensitive Analysis for Dried Blood
Spot
Proteins by Sodium Carbonate Precipitation for Clinical Proteomics. J. Proteome Res..

[55] Hughes C. S., Moggridge S., Müller T., Sorensen P. H., Morin G. B., Krijgsveld J. (2019). Single-pot,
solid-phase-enhanced sample preparation
for proteomics experiments. Nat. Protoc..

[56] Daly L. A., Clarke C. J., Oswald S. O., Jankevics A., Brownridge P. J., Scheltema R. A., Eyers C. E. (2025). An Optimized SP3
Sample Processing Workflow for In-Depth and Reproducible Phosphoproteomics. J. Proteome Res..

[57] Vethe N. T., Åsberg A., Andersen A. M., Heier Skauby R., Bergan S., Midtvedt K. (2023). Clinical performance
of volumetric
finger-prick sampling for the monitoring of tacrolimus, creatinine
and haemoglobin in kidney transplant recipients. Br. J. Clin. Pharmacol..

[58] Sae-Lee W., Mccafferty C. L., Verbeke E. J., Havugimana P. C., Papoulas O., Mcwhite C. D. (2022). The protein organization
of a red blood cell. Cell Rep..

[59] Fernando S., Polkinghorne K. R. (2020). Cystatin
C: not just a marker of kidney function. Bra.
J. Nephrol..

[60] Hope C. M., Welch J., Mohandas A., Pederson S., Hill D., Gundsambuu B. (2019). Peptidase inhibitor 16 identifies a human regulatory
T-cell subset with reduced FOXP3 expression over the first year of
recent onset type 1 diabetes. Eur. J. Immunol..

[61] Chambers A. G., Percy A. J., Hardie D. B., Borchers C. H. (2013). Comparison of Proteins
in Whole Blood and Dried Blood Spot Samples by LC/MS/MS. J. Am. Soc. Mass Spectrom..

[62] Nordfalk F., Ekstrøm C. T. (2019). Newborn
dried blood spot samples in Denmark: the hidden
figures of secondary use and research participation. Eur. J. Hum. Genet..

[63] Li J., Chen J., Lan H.-Y., Tang Y. (2023). Role of C-Reactive
Protein in Kidney Diseases. Kidney Dis..

[64] Kristiansson A., Davidsson S., Johansson M. E., Piel S., Elmér E., Hansson M. J. (2020). α1-Microglobulin (A1M) Protects Human
Proximal Tubule Epithelial Cells from Heme-Induced Damage In Vitro. In. J. Mol. Sci..

[65] Gertow J., Ng C. Z., Mamede
Branca R. M., Werngren O., Du L., Kjellqvist S. (2017). Altered Protein Composition of Subcutaneous
Adipose Tissue in Chronic Kidney Disease. Kidney
Int. Re..

[66] Jasjit, Jeng, Saeed (2005). Oxalate-inducible AMBP gene and its
regulatory mechanism in renal tubular epithelial cells. Biochem. J..

[67] Luo Z., Lei H., Sun Y., Liu X., Su D.-F. (2015). Orosomucoid, an
acute response protein with multiple modulating activities. J. Physiol. Biochem..

[68] Higuchi H., Kamimura D., Jiang J.-J., Atsumi T., Iwami D., Hotta K. (2020). Orosomucoid
1 is involved in the development of chronic
allograft rejection after kidney transplantation. Int. Immunol..

[69] Chen T., Liu Y., Wu S., Long S., Feng L., Lu W. (2024). The association of RBP4 with chronic kidney diseases in southern
Chinese population. Front. Endocrinol..

[70] Ratajczyk K., Konieczny A., Czekaj A., Piotrów P., Fiutowski M., Krakowska K. (2022). The Clinical Significance
of Urinary Retinol-Binding Protein 4: A Review. Int. J. of Environ. Res. Public Health..

[71] Wang W., Xu D., Zhong L., Zhang W., Kang J., Zhou J. (2019). Quantitative
proteomics reveals TMOD1-related proteins associated
with water balance regulation. PLoS One.

[72] Meng X.-M., Wang L., Nikolic-Paterson D. J., Lan H.-Y. (2025). Innate immune cells
in acute and chronic kidney disease. Nat. Rev.
Nephrol..

[73] Dunkelberger J. R., Song W.-C. (2010). Complement and its role in innate
and adaptive immune
responses. Cell Res..

[74] Ferraboschi P., Ciceri S., Grisenti P. (2021). Applications
of Lysozyme, an Innate
Immune Defense Factor, as an Alternative Antibiotic. Antibiotics.

[75] Convertini P., Santarsiero A., Todisco S., Gilio M., Palazzo D., Pappalardo I. (2023). ACLY as a modulator
of liver cell functions
and its role in Metabolic Dysfunction-Associated Steatohepatitis. J. Transl. Med..

[76] Pei J., Zhang J., Yu C., Luo J., Wen S., Hua Y., Wei G. (2026). Selenoprotein GPX3 regulates NADPH oxidase expression
by inhibiting the MAPK signaling pathway and thereby attenuating the
inflammatory response in renal ischemia-reperfusion injury. Genes Dis..

[77] Fearn A., Sheerin N. (2015). Sheerin NS. Complement activation
in progressive renal
disease. World J. Nephrol..

[78] Willows J., Brown M., Sheerin N. S. (2020). The role
of complement in kidney
disease. Clin. Med..

[79] Dubin R. F., Deo R., Ren Y., Wang J., Zheng Z., Shou H. (2023). Proteomics
of CKD progression in the chronic renal insufficiency
cohort. Nat. Commun..

[80] Chen C., Zhang J., Yu T., Feng H., Liao J., Jia Y. (2024). LRG1 Contributes to
the Pathogenesis of Multiple Kidney Diseases:
A Comprehensive Review. Kidney Dis..

[81] Tao Y., Liu Y., Wang Z., Tang L., Zhang Y., Zheng S. (2025). Lumican
as a potential biomarker for diabetic nephropathy. Renal Failure.

[82] Canas J. J., Arregui S. W., Zhang S., Knox T., Calvert C., Saxena V. (2024). *DEFA1A3*DNA gene-dosage regulates the
kidney innate immune response during upper urinary tract infection. Life Sci. Alliance.

[83] Schwaiger J. P., Kollerits B., Steinbrenner I., Weissensteiner H., Schönherr S., Forer L. (2021). Apolipoprotein A-IV
concentrations and clinical outcomes in chronic kidney disease patients:
Results from the German Chronic Kidney Disease (GCKD) study. Atherosclerosis.

