# Supplementary material for: Optimized Hemoglobin Depletion Improves Clinical Proteomics from Dried Blood Spots
Source: J Proteome Res. 2026 Oct 16;25(11):6151–65. doi: 10.1021/acs.jproteome.6c00534 (PMC13750103; doi:10.1021/acs.jproteome.6c00534)
Supplement: Supplementary file 1 [file pr6c00534_si_001.pdf]

Optimised haemoglobin depletion improves clinical proteomics from dried blood spots

Hannah Ging[1,2,3], Rosemary E. Maher[1,2], Elin Davies[3,4,5], Philip J. Brownridge[1,2], Anirudh Rao[4], Alan D. Salama[6], Louise Oni[3,5,6,7], Claire E. Eyers[1,2], Andrew J. Chetwynd[1,2,3*]

[1] Centre for Proteome Research, Faculty of Health & Life Sciences, University of Liverpool, Liverpool, United Kingdom L69 7ZB

[2]Department of Biochemistry, Cell and Systems Biology, Institute of Systems, Molecular and Integrative Biology, University of Liverpool, Liverpool, United Kingdom L69 7BE

[3] UK's Experimental Arthritis Treatment Centre for Children, University of Liverpool, Liverpool, United Kingdom L14 5AB

[4] Department of Nephrology, Liverpool University Hospitals NHS Foundation Trust, Liverpool, United Kingdom L7 8YE

[5] Department of Women's and Children's Health, Institute of Life Course and Medical Sciences, University of Liverpool, Liverpool, United Kingdom L7 8TX

[6] Department of Renal Medicine, University College London, UCL Medical School, Rowland Hill Street, London, United Kingdom NW3 2PF

[7] Department of Paediatric Nephrology, Great Ormond Street Hospital for Children, London, United Kingdom WC1N 3JH

*To whom correspondence should be addressed

a.j.chetwynd@liverpool.ac.uk

Supplementary Information

Materials and Methods 3

Haemoglobin Depletion 3

Protein Digestion 3

LC-MS/MS 4

Results and Discussion 5

Optimisation of Sample Reconstitution Buffer 5

Comparison of Plasma, Dried Plasma and Dried Blood 10

Evaluation of High Abundance Protein Depletion Techniques 11

Cell Free Dried Blood Spot Analysis 15

Proof-of-Principle Clinical Validation in Chronic Kidney Disease 17

Materials and Methods

Unless otherwise specified, all reagents were sourced from Thermo Fisher Scientific, Waltham, MA, United States.

Haemoglobin Depletion

HemogloBind Depletion was performed as per manufacturer's instructions, with 20 µL of reconstituted blood and 200 µL 0.02 M Dipotassium Phosphate (pH 6.5) added to a spin-filter tube, and vortexed for 5 minutes. The HemogloBind suspension (Biotech Support Group, South Brunswick, NJ, United States) was vortexed, and 200 µL added, and vortexed at RT for 10 minutes. The sample was centrifuged for 4 minutes at 15000 *g*.

NuGel-HemogloBind Depletion was performed as per manufacturer's instructions, with 50 mg of NuGel-HemogloBind Beads (Biotech Support Group, South Brunswick, NJ, United States) added to a spin-filter tube and conditioned with 200 µL of haemoglobin binding buffer (HBB) (Biotech Support Group, South Brunswick, NJ, United States). The tube was vortexed for 2 minutes, followed by centrifugation at 15000 *g* for 4 minutes. The filtrate was discarded. To a separate tube, 20 µL of blood was diluted in 400 µL of HBB and vortexed for 3 minutes, before being added to the beads. The mixture was vortexed for 10 minutes, followed by centrifugation at 15000 *g* for 4 minutes.

HemoVoid depletion was performed as per manufacturer's directions, with all measurements halved to account for reduced blood volume. Therefore, 25 mg of HemoVoid matrix (Biotech Support Group, South Brunswick, NJ, United States) was added to a microfuge tube, with 125 µL of haemoglobin binding buffer (HBB) (Biotech Support Group, South Brunswick, NJ, United States). The tube was vortexed, followed by centrifugation at 15000 *g* for 2 minutes. The filtrate was discarded, and 125 µL of HBB was added. The vortex and centrifuge steps were then repeated. An additional 150 µL of HBB, and 150 µL of blood sample was added. The sample was vortexed for 10 minutes, followed by centrifugation at 15000 *g* for 4 minutes. The filtrate was removed, and 250 µL of wash buffer (Biotech Support Group, South Brunswick, NJ, United States) was added. The sample was vortexed for 5 minutes, followed by centrifugation at 15000 *g* for 4 minutes, and the filtrate was discarded as wash. The washing step was repeated twice more. To the beads, 150 µL of elution buffer (Biotech Support Group, South Brunswick, NJ, United States) was added, and the sample was vortexed for 10 minutes. Finally, the sample was centrifuged for 4 minutes at 15000 *g*. In all cases the depleted sample was the final filtrate.

Protein Digestion

SP3 digestion was performed in a semi-skirted 96-well plate, using 25 µL of depleted blood sample. Samples were reduced and alkylated with 10 µL of Tris(2-carboxyethyl)phosphine (TCEP)/Chloracetamide (CAA) (120 mM/480 mM). After incubation for 5 minutes at 95 °C, the plate was centrifuged at 300 *g* for 1 minute then 10 µL of washed and combined Sera-Mag SP3 beads (E3 and E7) (Cytiva, Danaher, Washington DC, USA) was added. Next, 130 µL 100% ethanol were added and mixed. The plate was incubated for 10 minutes, before a magnetic rack was used to pellet the beads, and the supernatant was removed. The pellets were resuspended in 300 µL of 80% (v/v) ethanol and water, and, using a magnetic rack to pellet the beads, the supernatant was removed. This

washing process was repeated for a total of six times. The pellets were dried using the SpeedVac (Centrifuge: UNIVAPO – 150 ECH, Cooling unit: UNICRYO MC2L -60 °C, Vacuum pump: UNIVAC DQ4) for 10 minutes. Samples were resuspended in 150 µL 100 mM Ammonium Bicarbonate with 300 ng LysC (Promega, Madison, WI, United States), and incubated in a ThermoMixer for 2 hours at 37 °C and 1450 rpm. Trypsin (Promega, Madison, WI, United States) was then added at 50:1 Protein:Trypsin, and the plate was incubated overnight at 37 °C and 1450 rpm. A magnetic rack was used to pellet the SP3 beads, and the supernatant was transferred to a new 96-well plate before being acidified with 5 µL 10% TFA and dried using the SpeedVac.

LC-MS/MS

EvoTips (EvoSep, Odense, Denmark) were prepared as per the manufacturer's instructions. They were washed with 20 µL 0.1% (v/v) FA (ACN) and centrifuged at 800 *g* for 1 minute. The tips were then conditioned using isopropyl alcohol, before washing with 20 µL 0.1% FA (v/v) (H_2O), and centrifugation at 800 *g* for 1 minute. Next 20 µL of sample (100 ng final peptide mass injected) was added, and the centrifugation step was repeated. The tips were then washed once more with 0.1% FA (H_2O), and centrifuged. To prevent the tips from drying out before analysis, 100 µL 0.1% FA (H_2O) was then added. Samples were ran with the following method https://www.evosep.com/wp-content/uploads/2021/05/AN-009A-30SPD.pdf

Results and Discussion

Optimisation of Sample Reconstitution Buffer

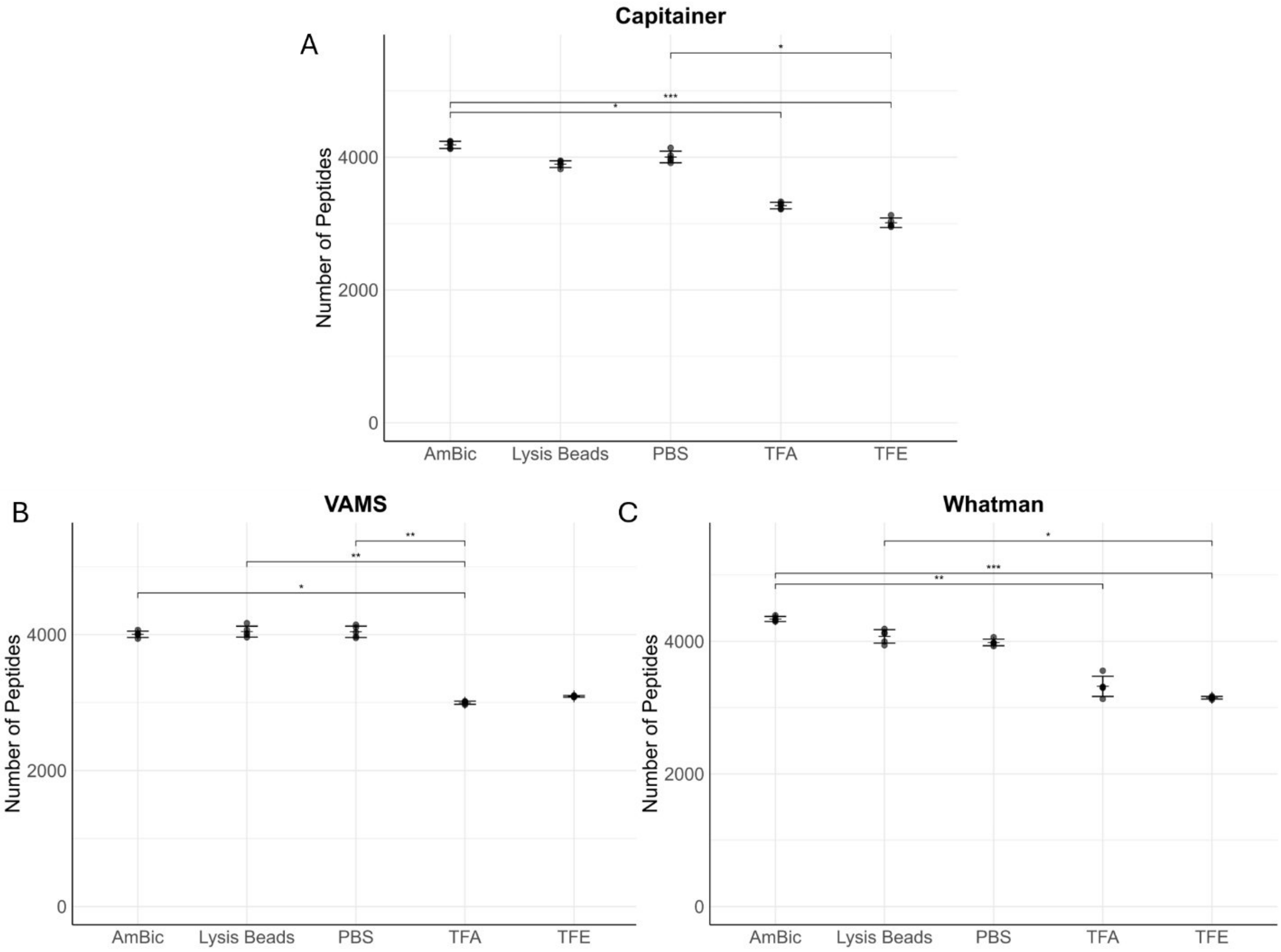

*Figure S1- Optimisation of Sample Reconstitution Buffer. (A) The average number of peptides identified in Capitainer b10 DBS with each reconstitution method (n= 5). (B) The average number of peptides identified in the Neoteryx VAMS Mitra DBS based on the reconstitution method (n= 5). (C) The average number of peptides identified in Whatman903 DBS with each reconstitution method (n= 5). *= p<0.05, **= p<0.01, ***= p<0.001, Error Bars= Standard Deviation. AmBic= Ammonium Bicarbonate, PBS= Phosphate Buffered Saline, TFA= Trifluoroacetic Acid, TFE= Tetrafluoroethylene.*

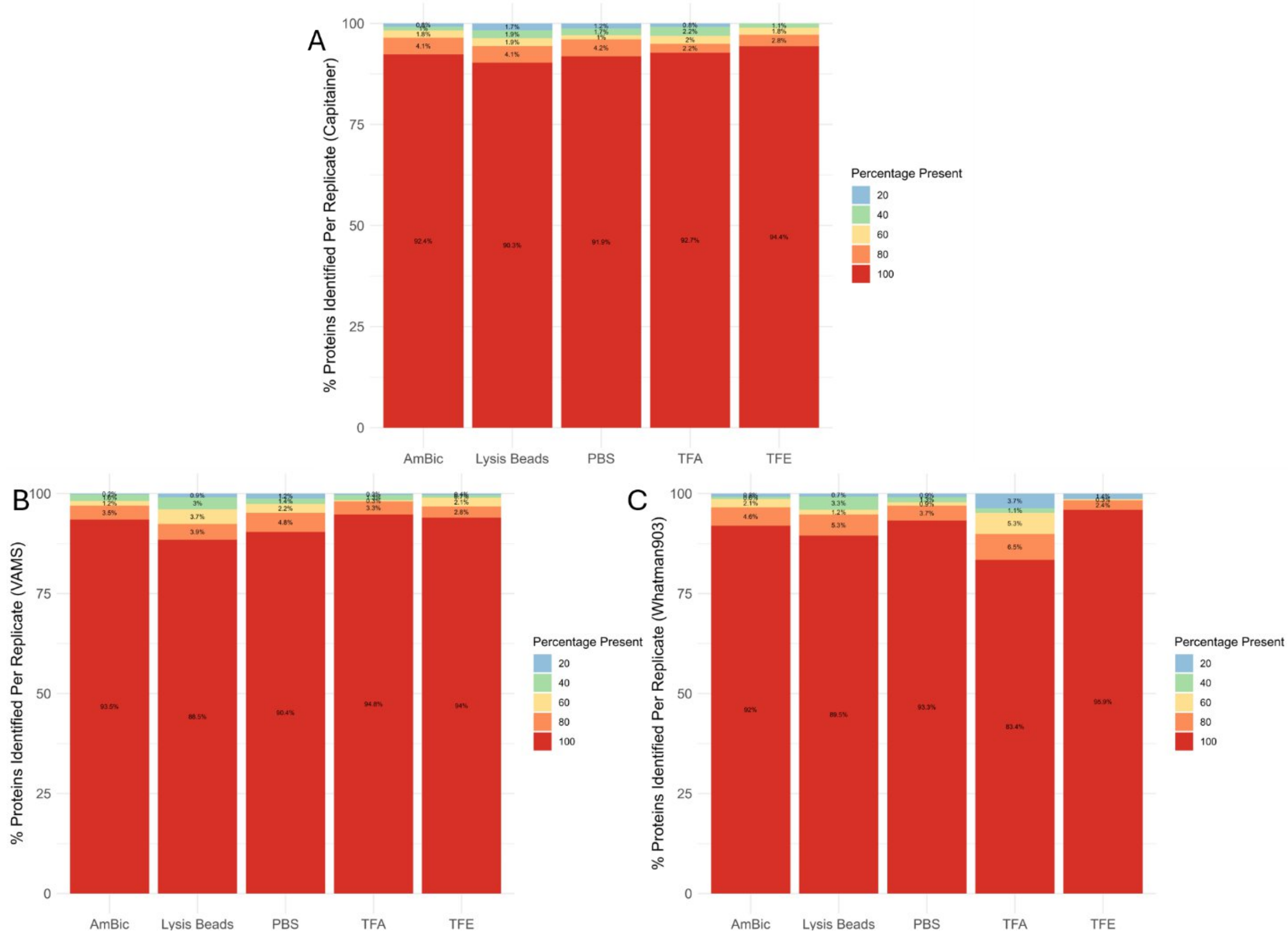

Figure S2- Optimisation of Sample Reconstitution Buffer for Capitainer b10 devices. (A) Bar chart showing the reproducibility of protein identifications using each reconstitution buffer (n= 5) with the Capitainer b10 devices. (B) Bar chart showing the reproducibility of protein identifications using each reconstitution buffer (n= 5) with the Neoteryx VAMs devices. (C) Bar chart showing the reproducibility of protein identifications using each reconstitution buffer (n= 5) with the Whatman903 devices.

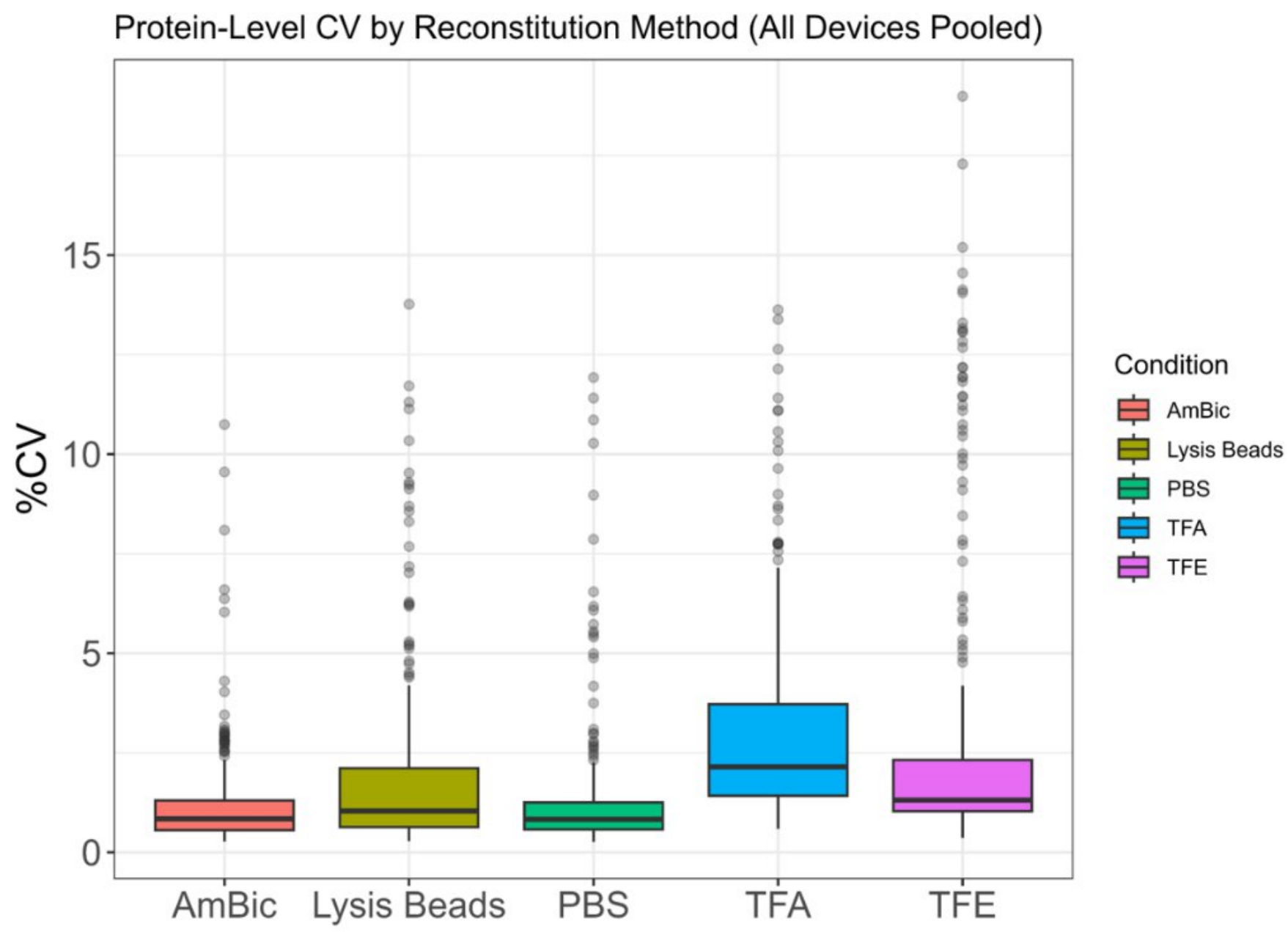

Figure S3- Optimisation of Sample Reconstitution Buffer. Box plot showing the percentage coefficient of variance of protein relative abundance using each reconstitution buffer across all 3 devices (n=15).

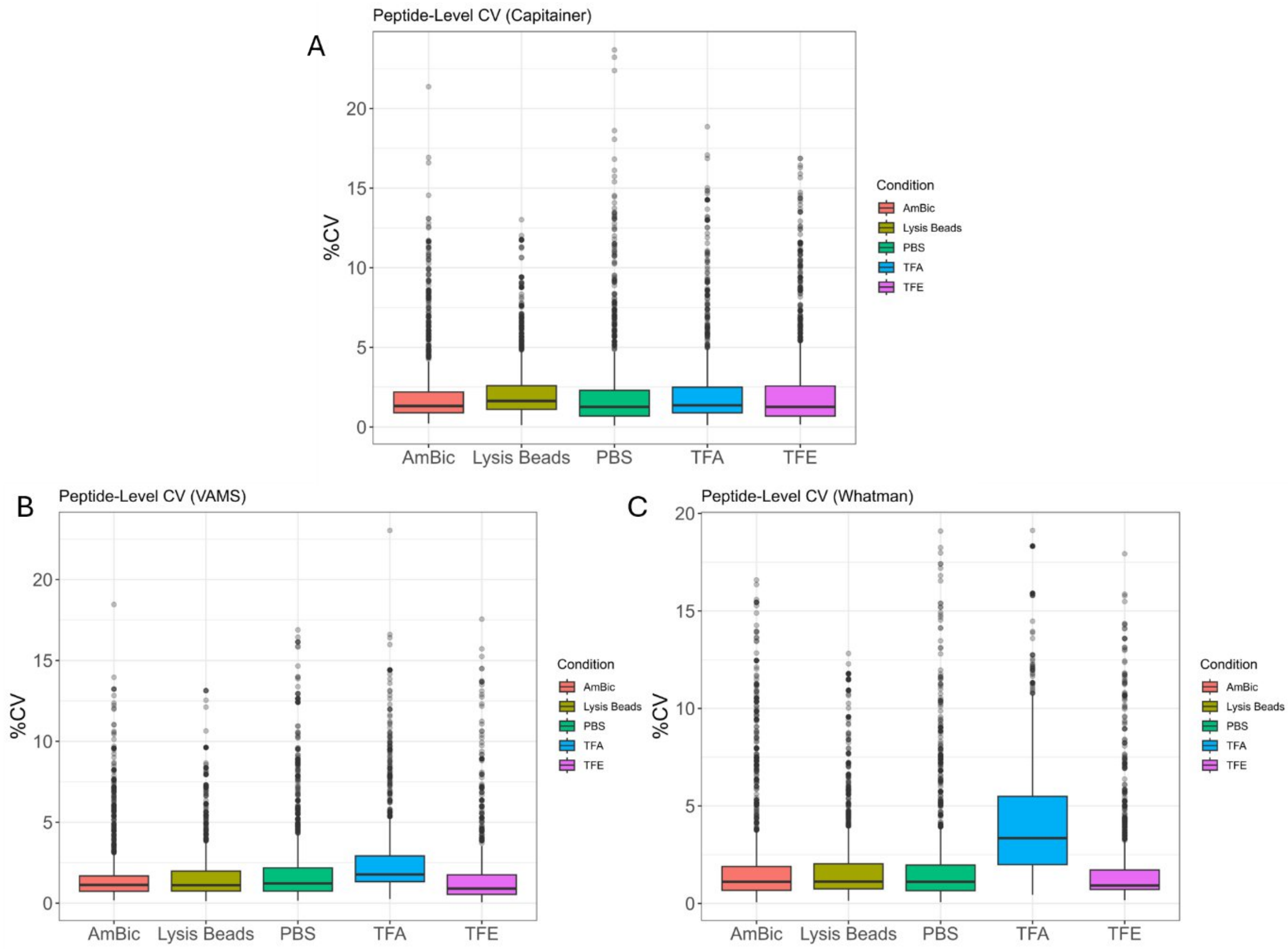

Figure S4- Optimisation of Sample Reconstitution Buffer. (A) Box plot showing the percentage coefficient of variance of protein relative abundance using each reconstitution buffer with Capitainer b10 devices (n=5). (B) Box plot showing the percentage coefficient of variance of protein relative abundance using each reconstitution buffer with Neoteryx VAMs devices (n=5). (C) Box plot showing the percentage coefficient of variance of protein relative abundance using each reconstitution buffer with Whatman903 devices (n=5).

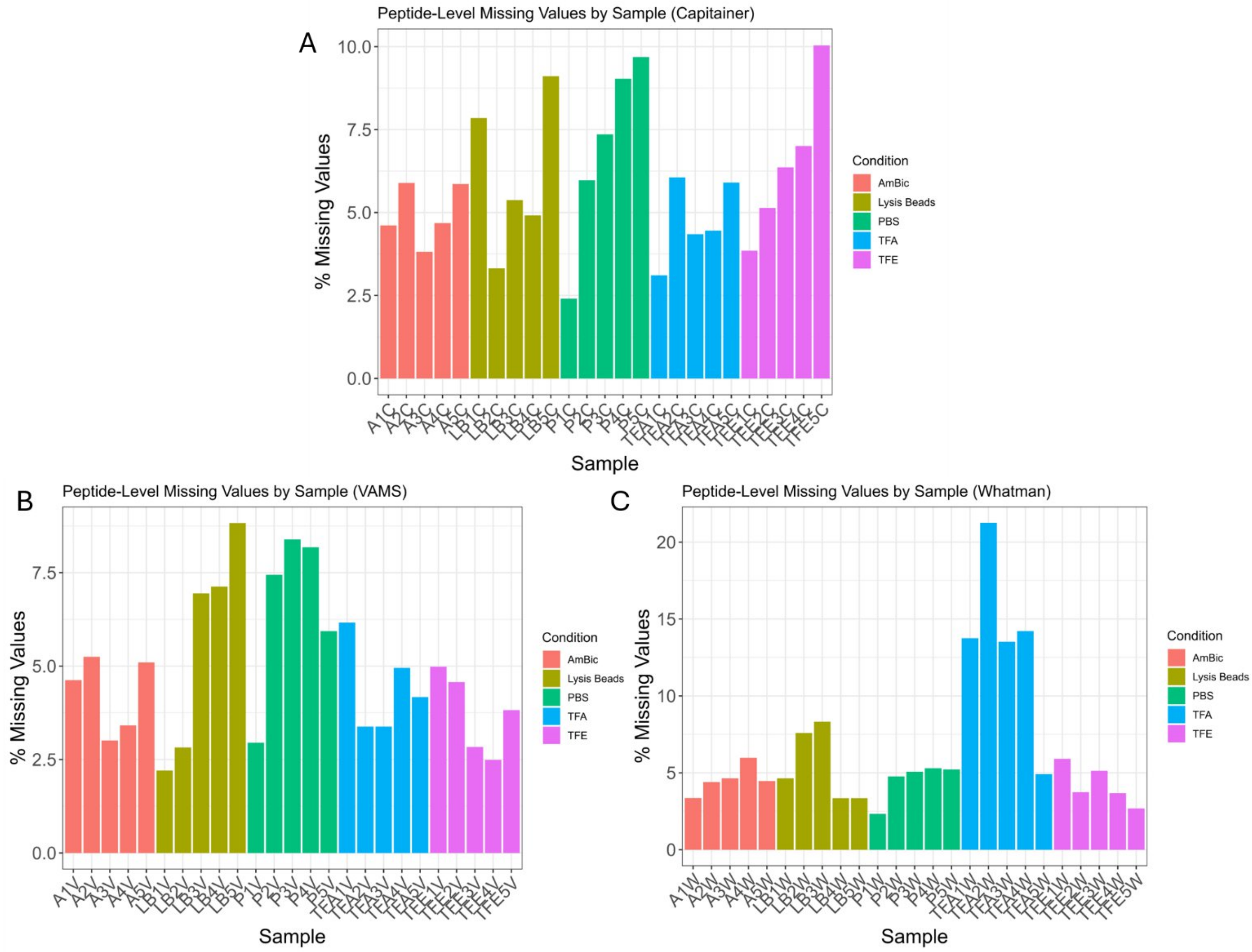

Figure S5- Optimisation of Sample Reconstitution Buffer. (A) Bar chart showing the percentage of missing peptide values within each condition using each reconstitution buffer with Capitainer b10 devices (n=5). (B) Bar chart showing the percentage of missing peptide values within each condition using each reconstitution buffer with Neoteryx VAMs devices (n=5). (C) Bar chart showing the percentage of missing peptide values within each condition using each reconstitution buffer with Whatman903 devices (n=5).

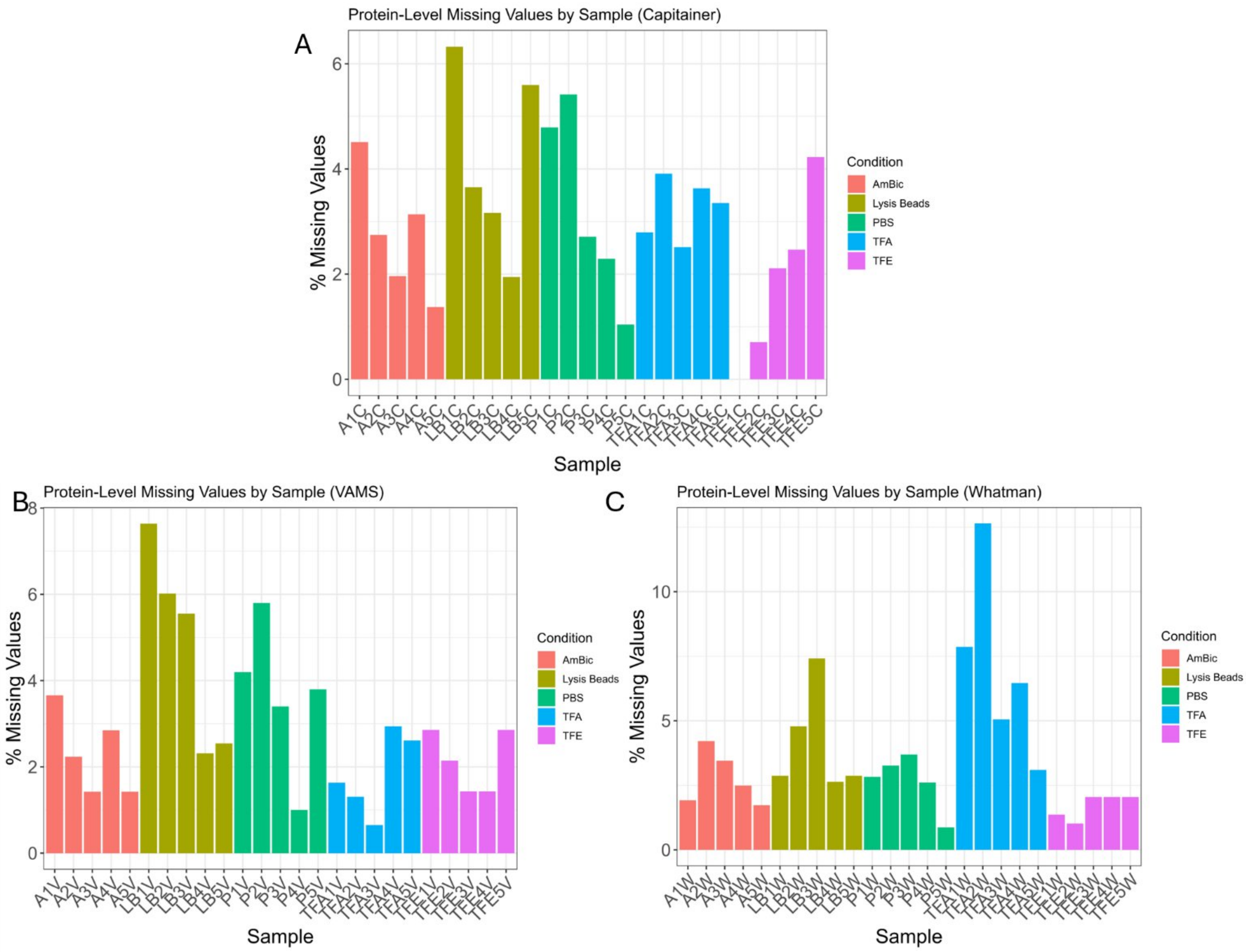

Figure S6- Optimisation of Sample Reconstitution Buffer. (A) Bar chart showing the percentage of missing protein values within each condition using each reconstitution buffer with Capitainer b10 devices (n=5). (B) Bar chart showing the percentage of missing protein values within each condition using each reconstitution buffer with Neoteryx VAMs devices (n=5). (C) Bar chart showing the percentage of missing protein values within each condition using each reconstitution buffer with Whatman903 devices (n=5).

Comparison of Plasma, Dried Plasma and Dried Blood

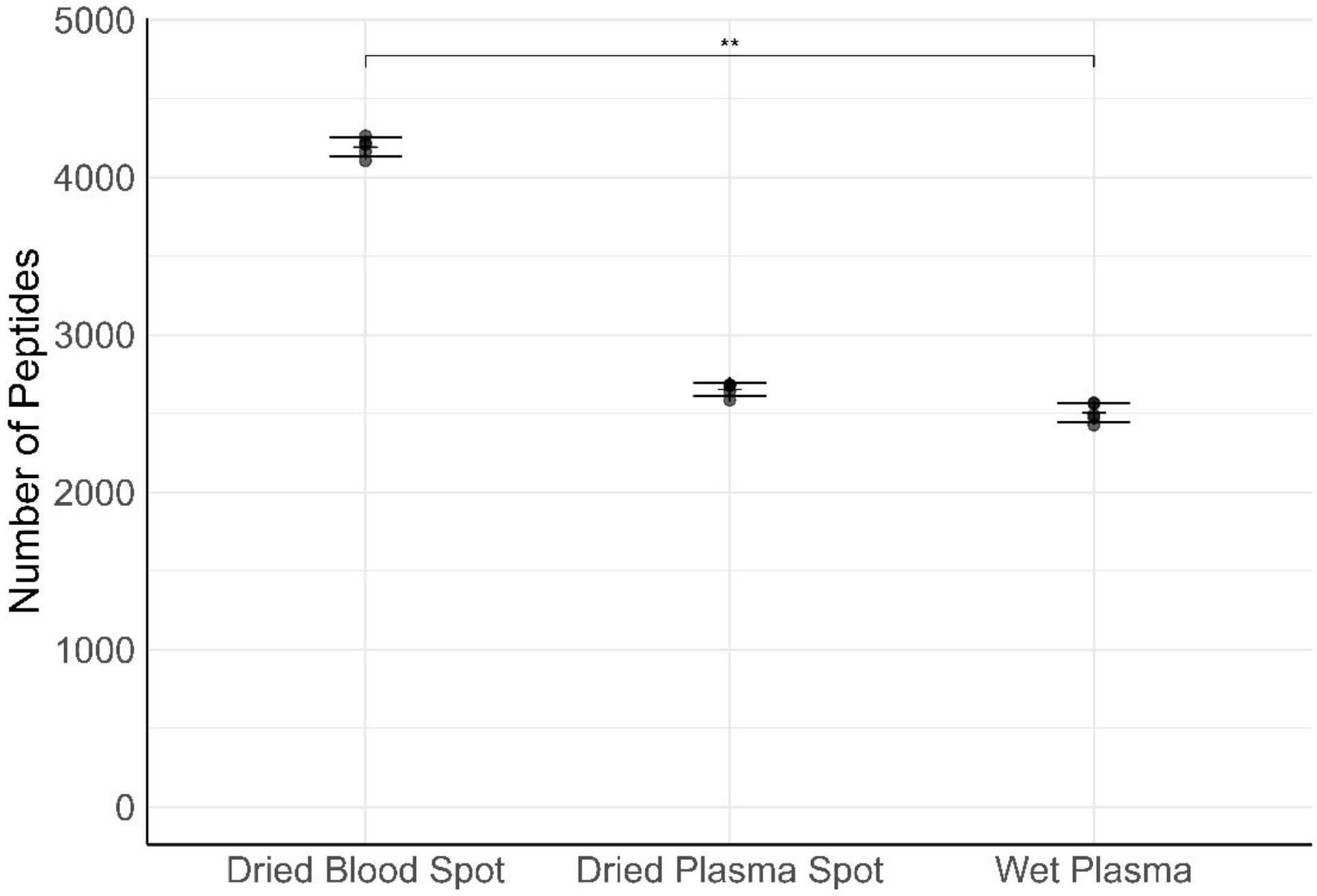

*Figure S7- Comparison of Plasma, Dried Plasma and Dried Blood. The average number of peptides identified in dried blood spot, dried plasma spot, and wet plasma samples (n= 5). **= p<0.01*

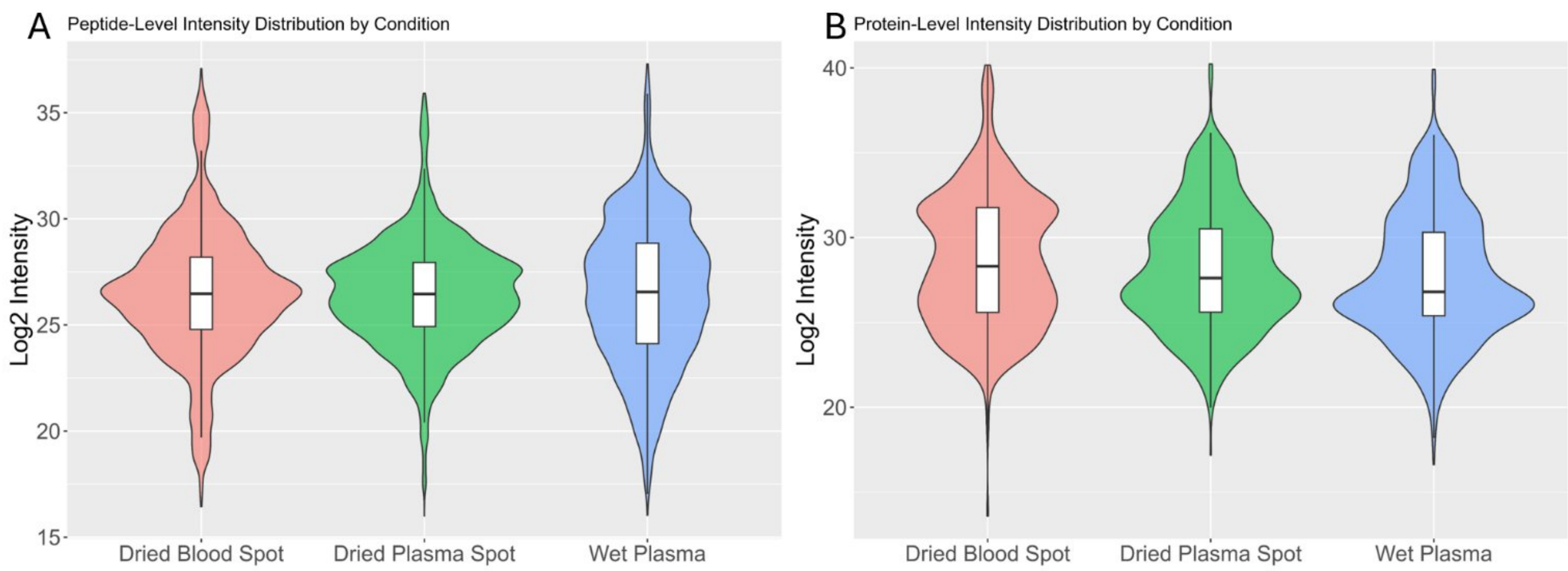

Figure S8- Peptide- and protein-level intensity distributions across sample types. Violin plots showing the distribution of log2-transformed (A) peptide and (B) protein intensity values for dried blood spot, dried plasma spot, and wet plasma samples. White boxplots indicate the median and interquartile range (IQR) within each violin.

Evaluation of High Abundance Protein Depletion Techniques

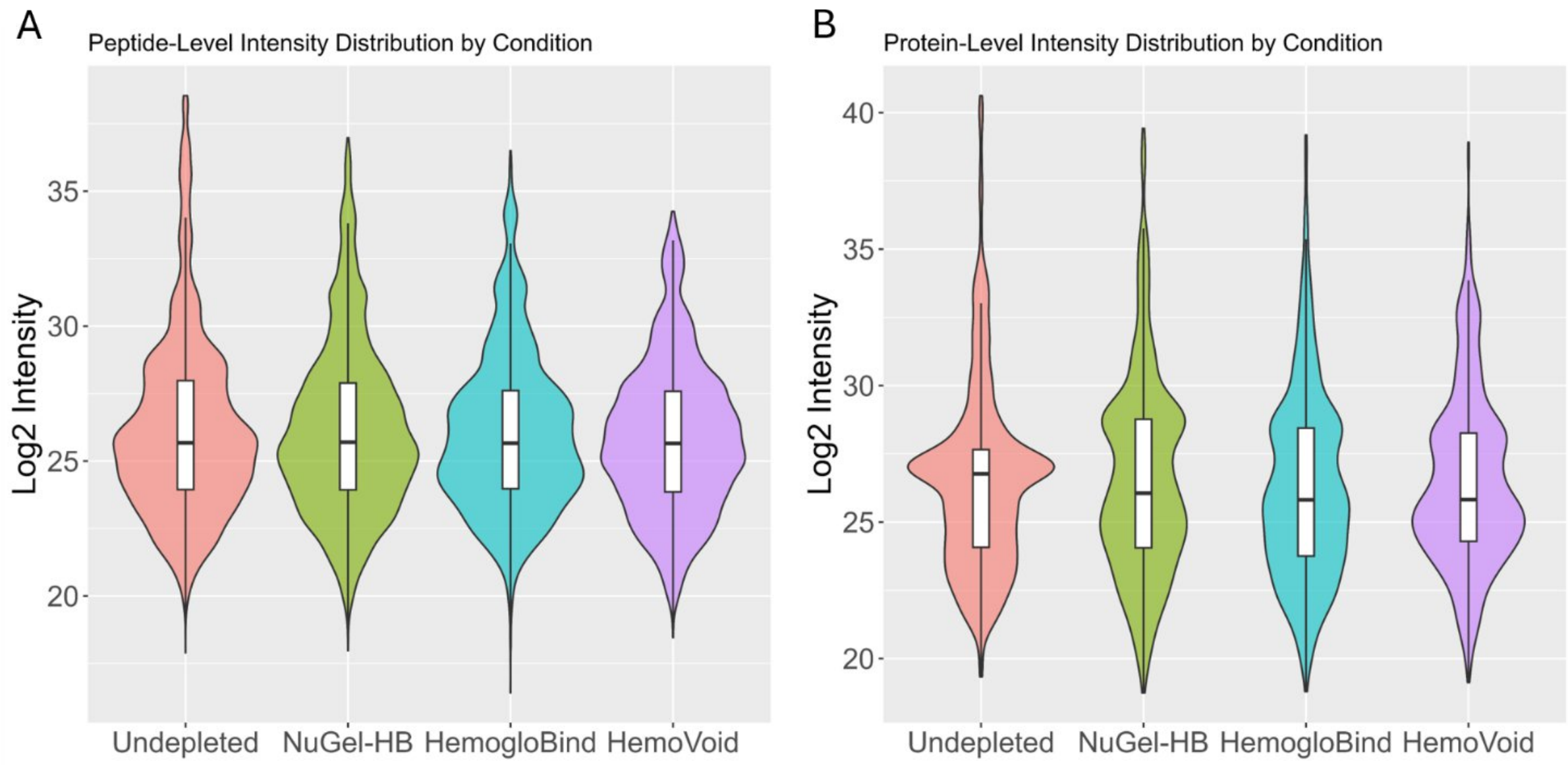

Figure S9- Peptide- and protein-level intensity distributions across sample types. Violin plots showing the distribution of log2-transformed (A) peptide and (B) protein intensity values for each depletion method. White boxplots indicate the median and interquartile range (IQR) within each violin.

Table S1- Evaluation of High Abundance Protein Depletion Techniques. Table showing the number of Haemoglobin Subunit proteins, peptides, peptide spectral matches (PSMs) and unique peptides identified using each depletion method (n= 5).

**Total (All Hg Subunits)**	**Undepleted**	**NuGel-HB**	**HemogloBind**	**HemoVoid**
**# Proteins**	9	7	7	8
**# Peptides**	96	87	78	42
**# PSMs**	4463	3274	2113	1434
**# Unique Peptides**	63	53	48	42

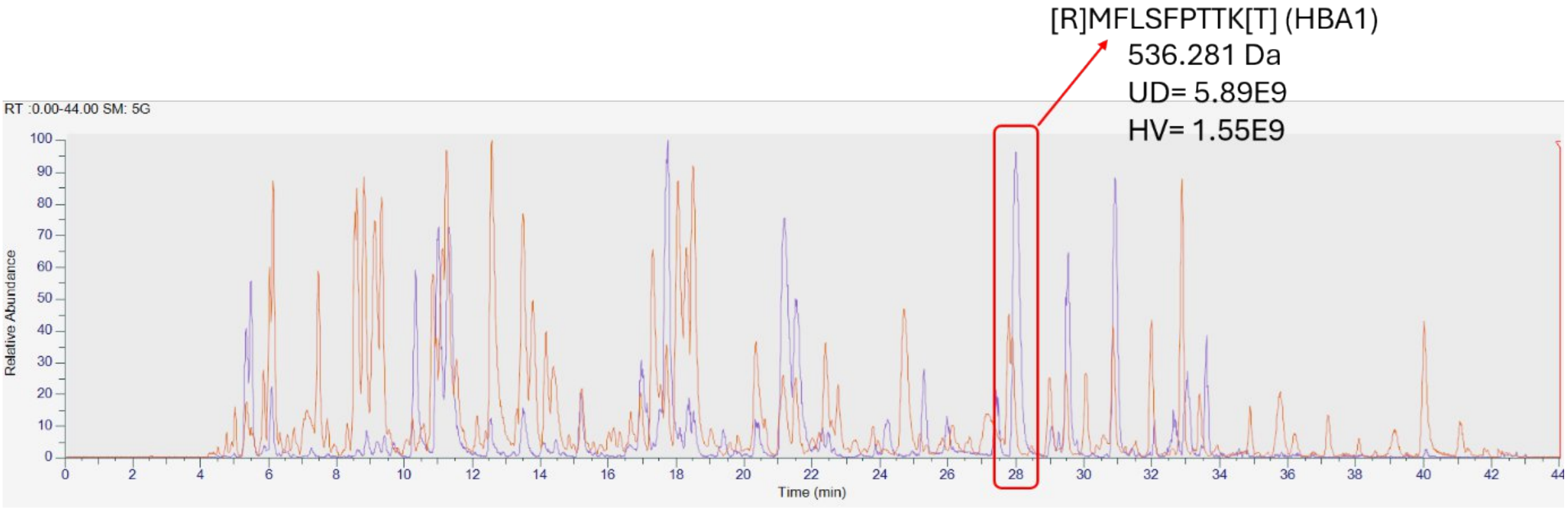

Figure S10- Base peak chromatogram demonstrating reduction of haemoglobin-derived peptide signal following HemoVoid depletion. Overlaid LC-MS/MS chromatographic traces show relative abundance for an undepleted plasma sample (orange) and a HemoVoid depleted sample (purple). The peak highlighted corresponds to the peptide MFLSFPTTK, derived from haemoglobin subunit alpha 1 (HBA1), with a monoisotopic mass of 536.281 Da. Peak area values for this haemoglobin peptide were reduced following HemoVoid treatment (HV = 1.55×10⁹) compared to the undepleted sample (UD = 5.89×10⁹). UD= Undepleted. HV= HemoVoid.

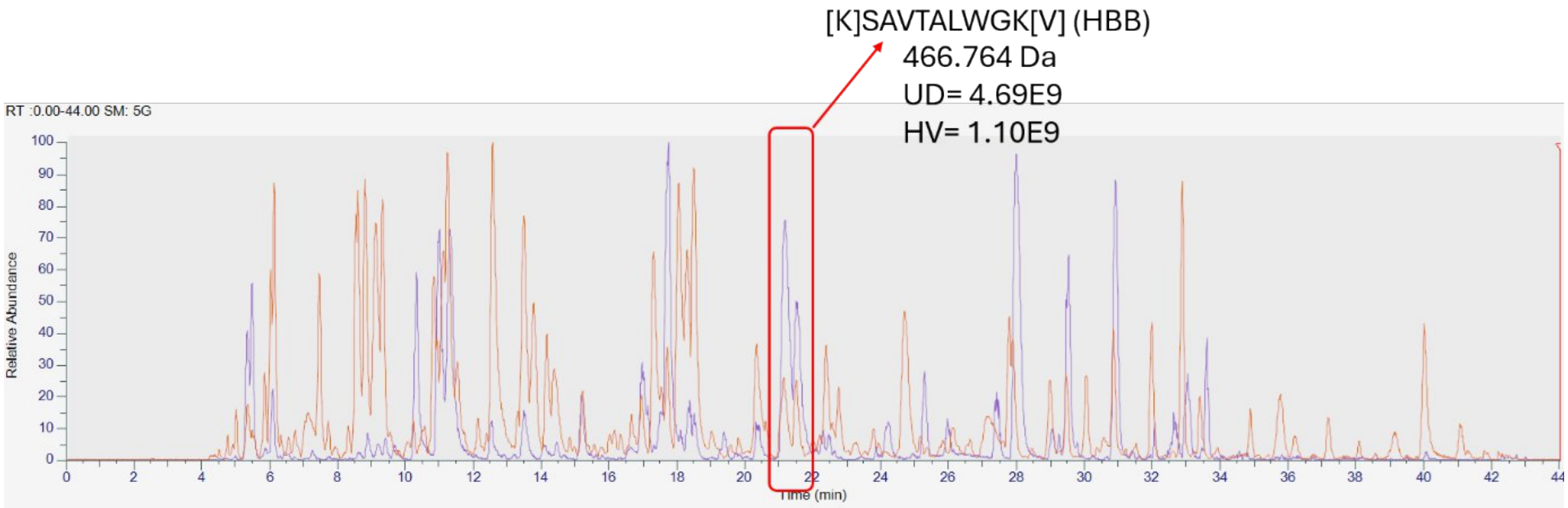

Figure S11- Base peak chromatogram demonstrating reduction of haemoglobin-derived peptide signal following HemoVoid depletion. Overlaid LC-MS/MS chromatographic traces show relative abundance for an undepleted plasma sample (orange) and a HemoVoid depleted sample (purple). The peak highlighted corresponds to the peptide SAVTALWGK, derived from haemoglobin subunit beta (HBB), with a monoisotopic mass of 466.764 Da. Peak area values for this haemoglobin peptide were reduced following HemoVoid treatment (HV = 1.10×10⁹) compared to the undepleted sample (UD = 4.69×10⁹). UD= Undepleted. HV= HemoVoid.

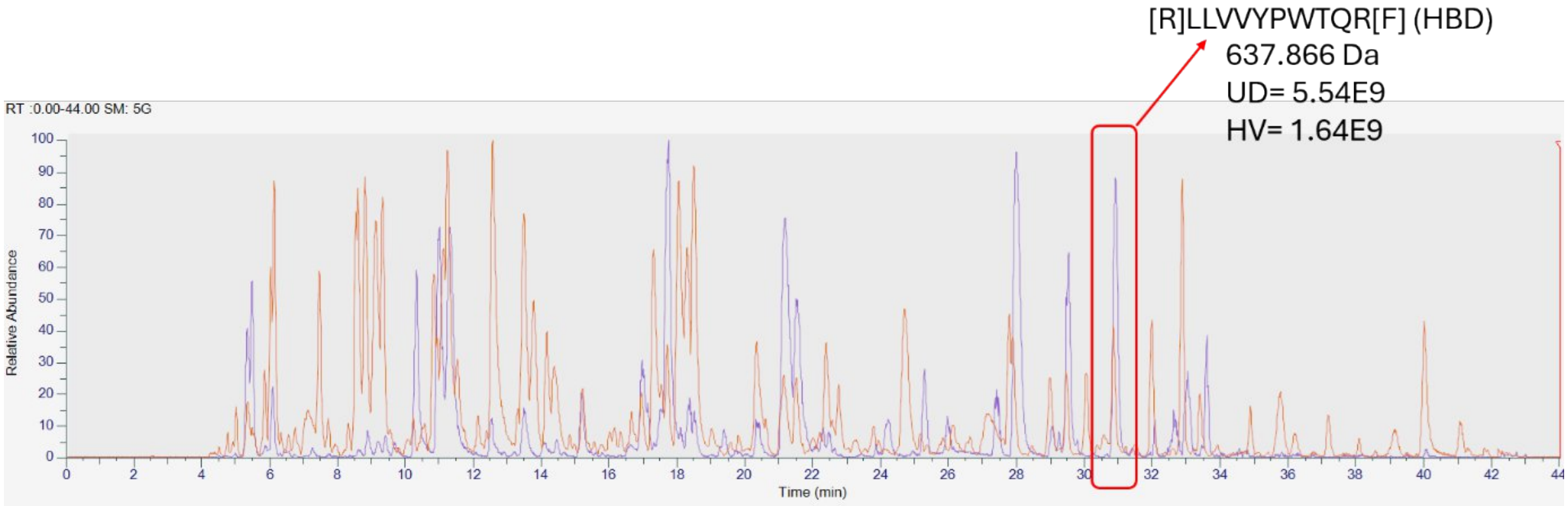

Figure S12- Base peak chromatogram demonstrating reduction of haemoglobin-derived peptide signal following HemoVoid depletion. Overlaid LC-MS/MS chromatographic traces show relative abundance for an undepleted plasma sample (orange) and a HemoVoid depleted sample (purple). The peak highlighted corresponds to the peptide LLVYPWTQR, derived from haemoglobin subunit delta (HBD), with a monoisotopic mass of 637.866 Da. Peak area values for this haemoglobin peptide were reduced following HemoVoid treatment (HV = 1.64×10⁹) compared to the undepleted sample (UD = 5.54×10⁹). UD= Undepleted. HV= HemoVoid.

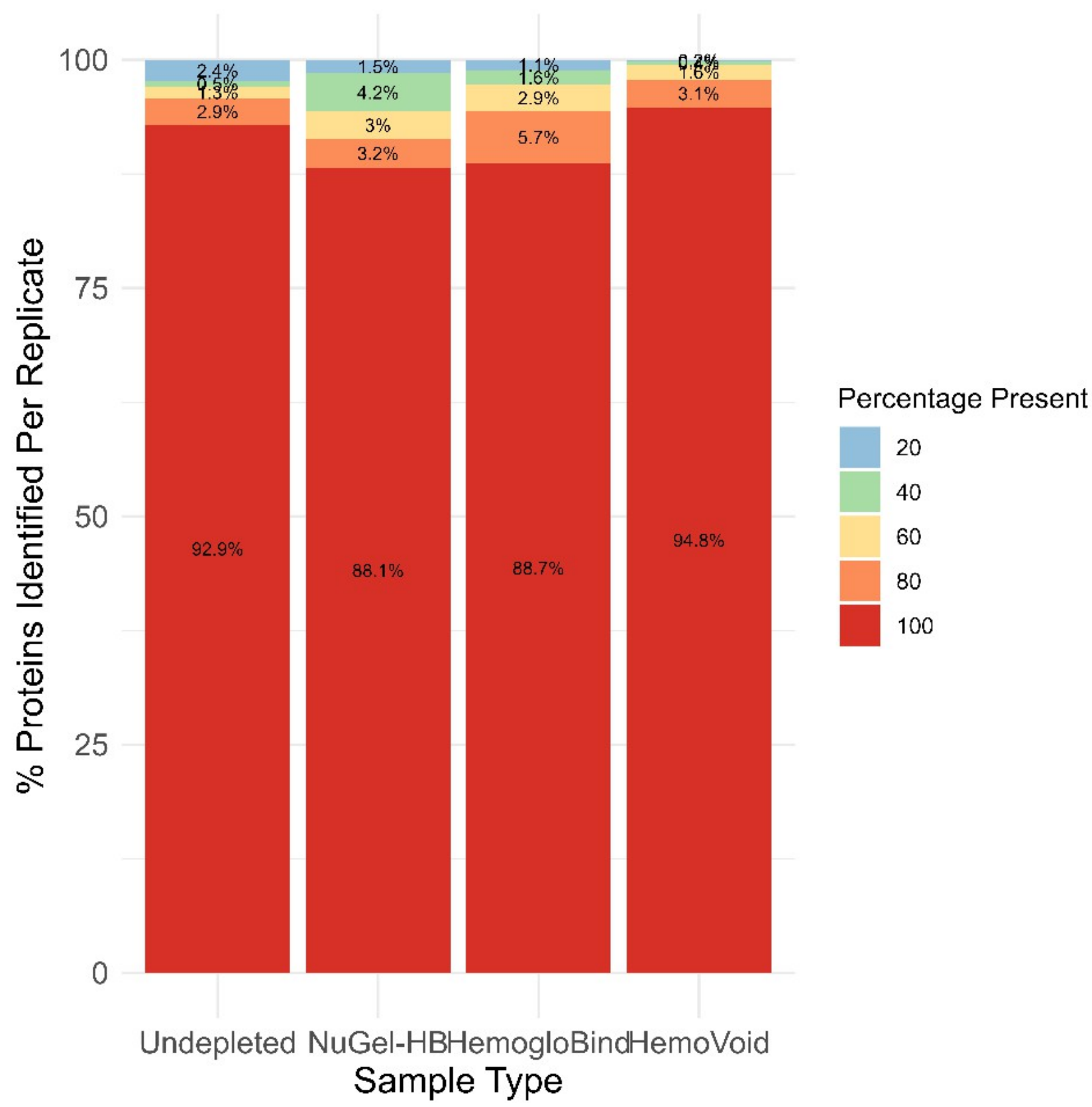

Figure S13- Evaluation of High Abundance Protein Depletion Techniques. Bar chart showing the reproducibility of protein identifications using each depletion method (n= 5).

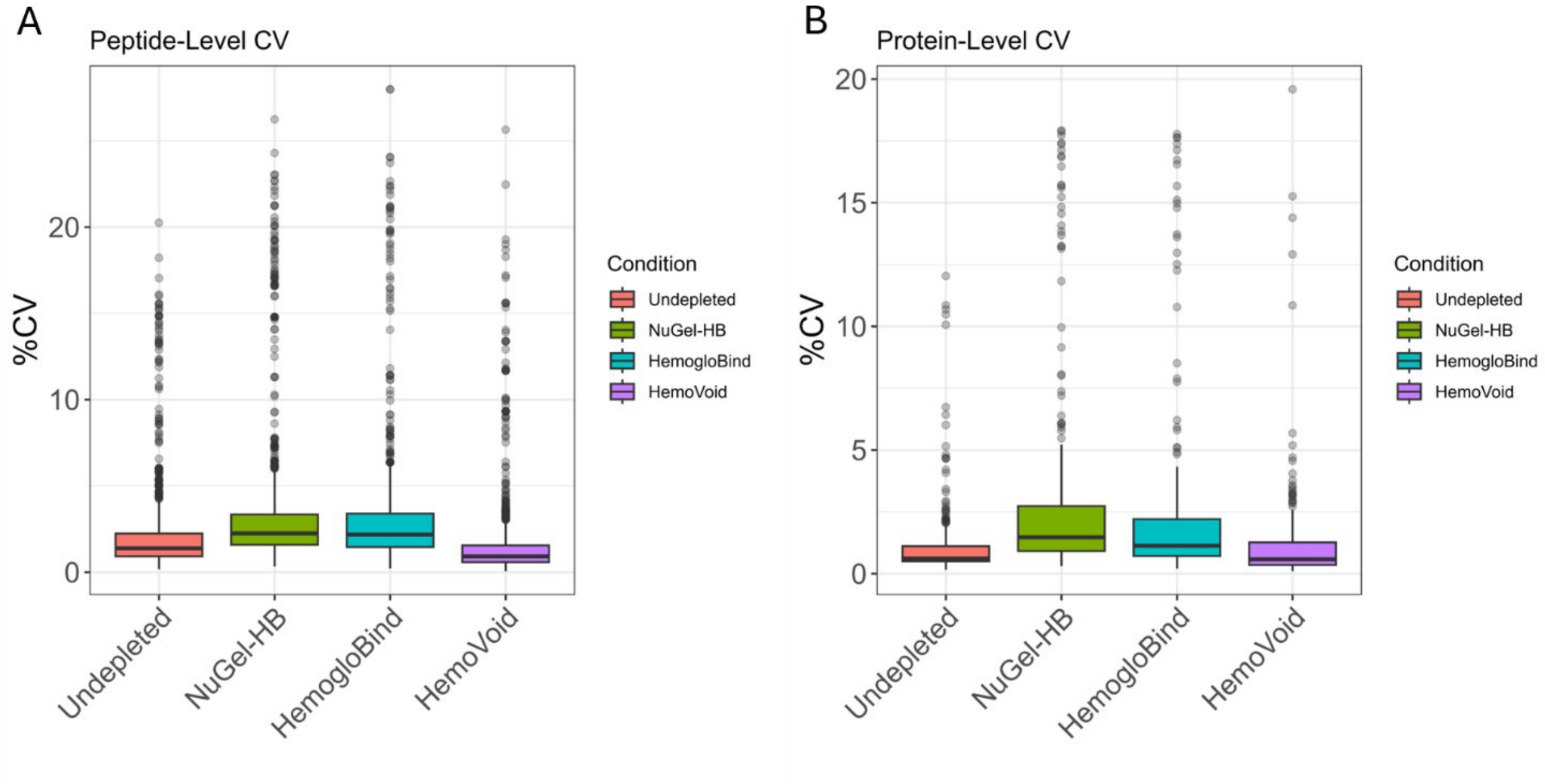

Figure S14- Evaluation of High Abundance Protein Depletion Techniques. Box plot showing the percentage coefficient of variance of (A) peptide and (B) protein relative abundance using each depletion method (n= 5).

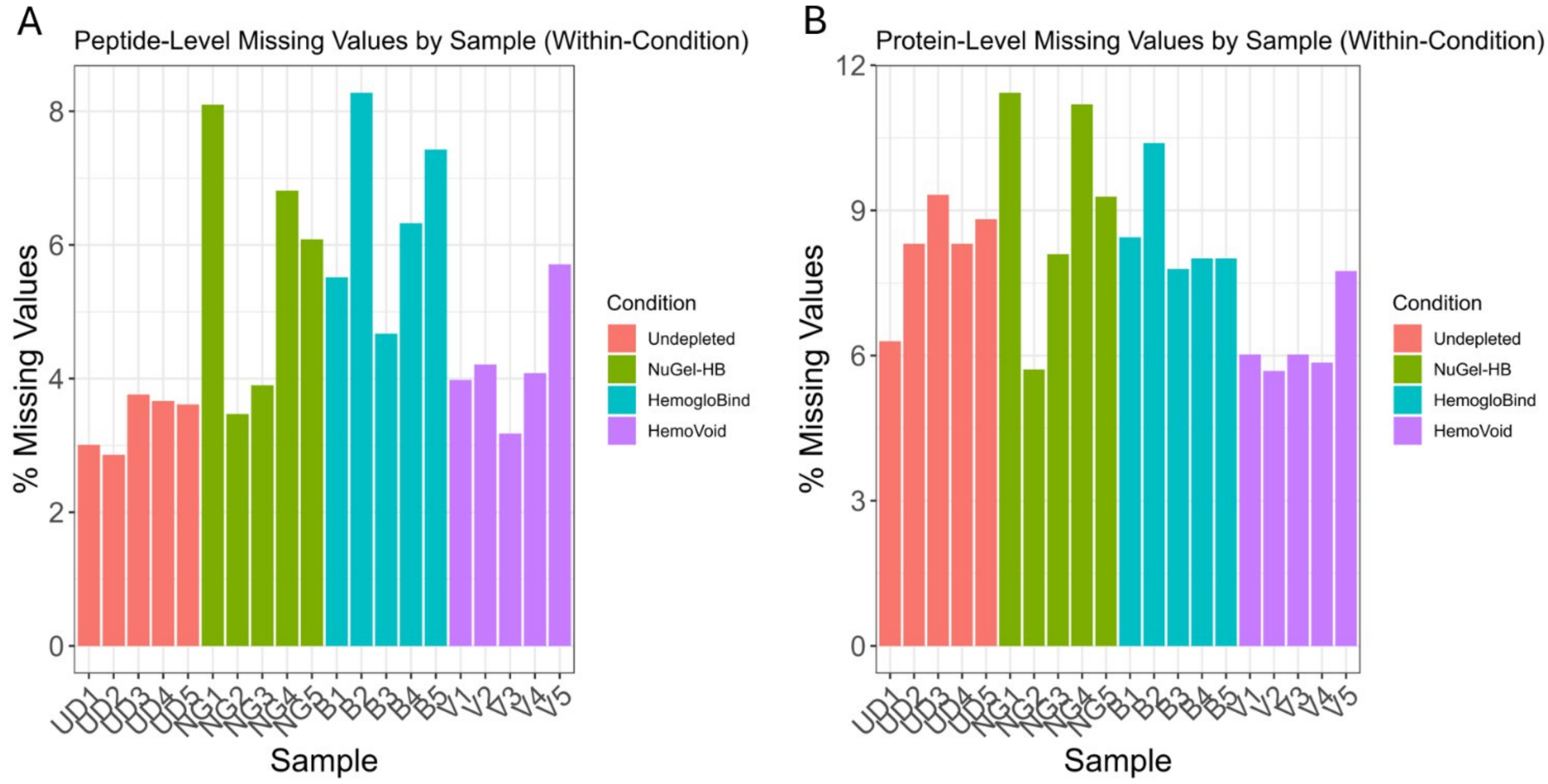

Figure S15- Evaluation of High Abundance Protein Depletion Techniques. (A) Bar chart showing the percentage of missing protein values within each condition using each depletion method (n=5). (B) Bar chart showing the percentage of missing protein values within each condition using each depletion method (n=5).

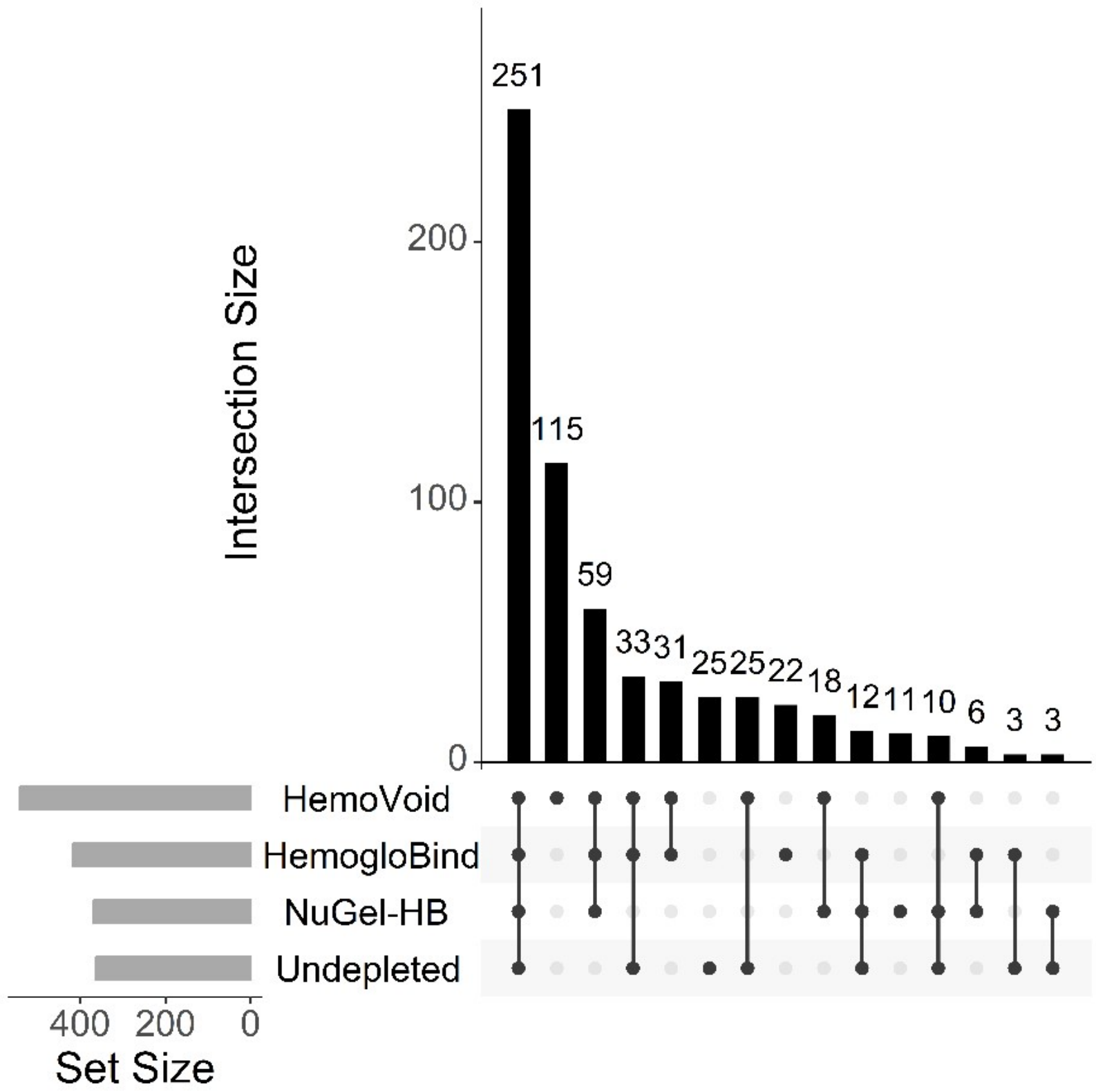

Figure S16- Evaluation of High Abundance Protein Depletion Techniques. Upset plot showing the cross over of proteins identified using each depletion method (n= 5).

Cell Free Dried Blood Spot Analysis

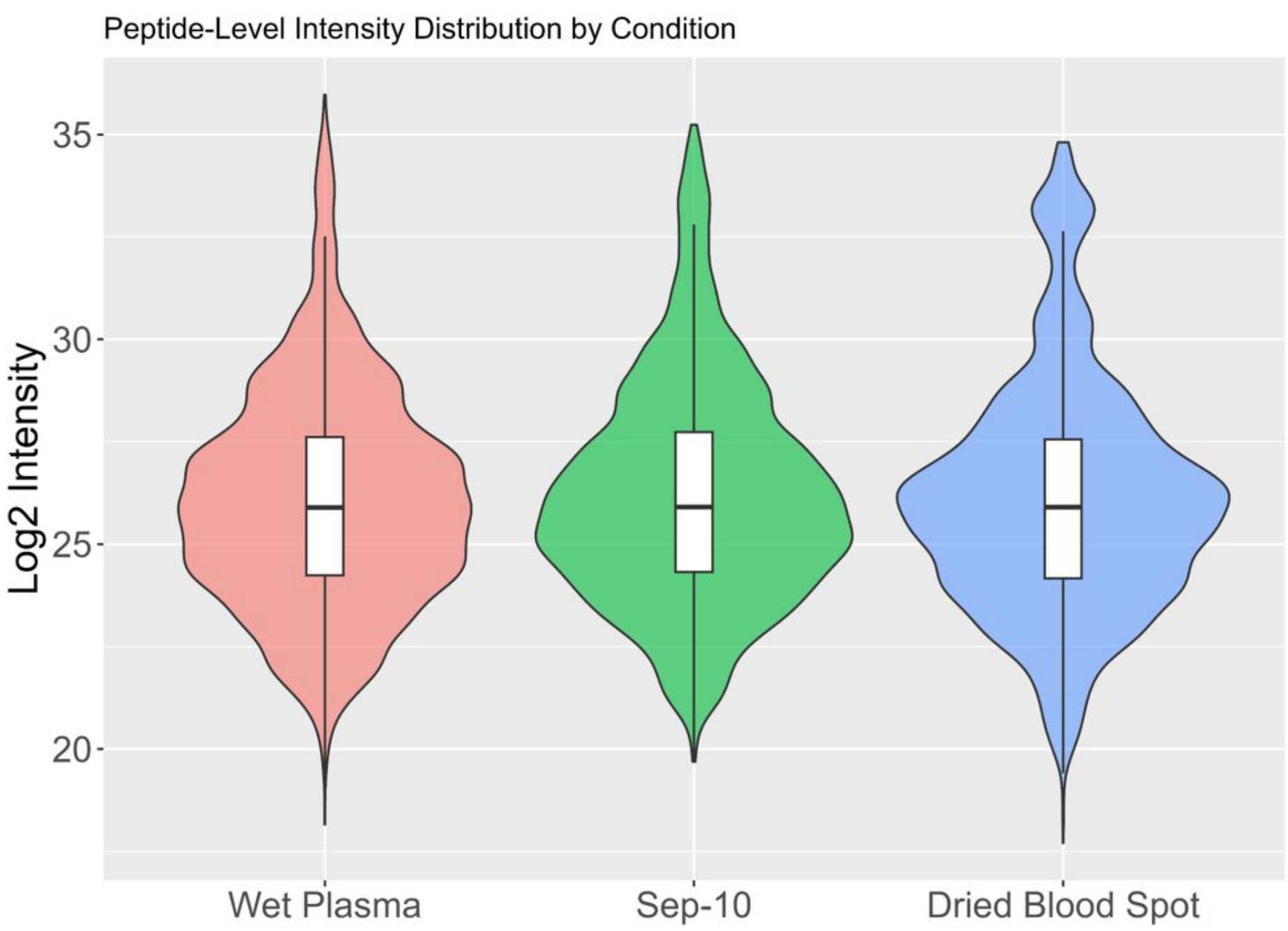

Figure S17- Peptide- and protein-level intensity distributions across sample types. Violin plots showing the distribution of log2-transformed (A) peptide and (B) protein intensity values for each depletion method. White boxplots indicate the median and interquartile range (IQR) within each violin.

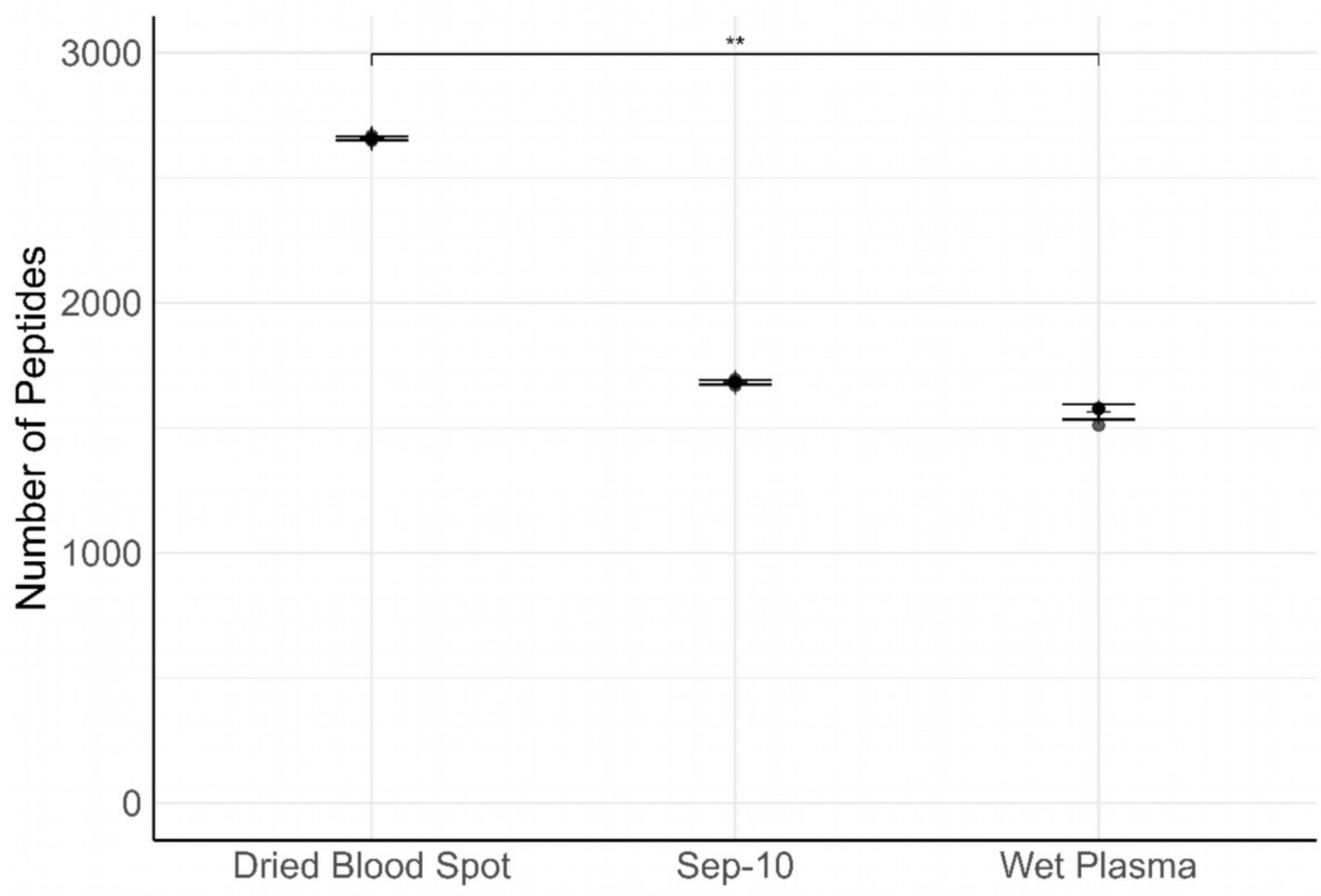

*Figure S18- Analysis of cell-free DBS compared to traditional DBS and plasma samples. The average number of peptides identified in dried blood spot, dried plasma spot, and wet plasma samples (n= 5). **= p<0.01*

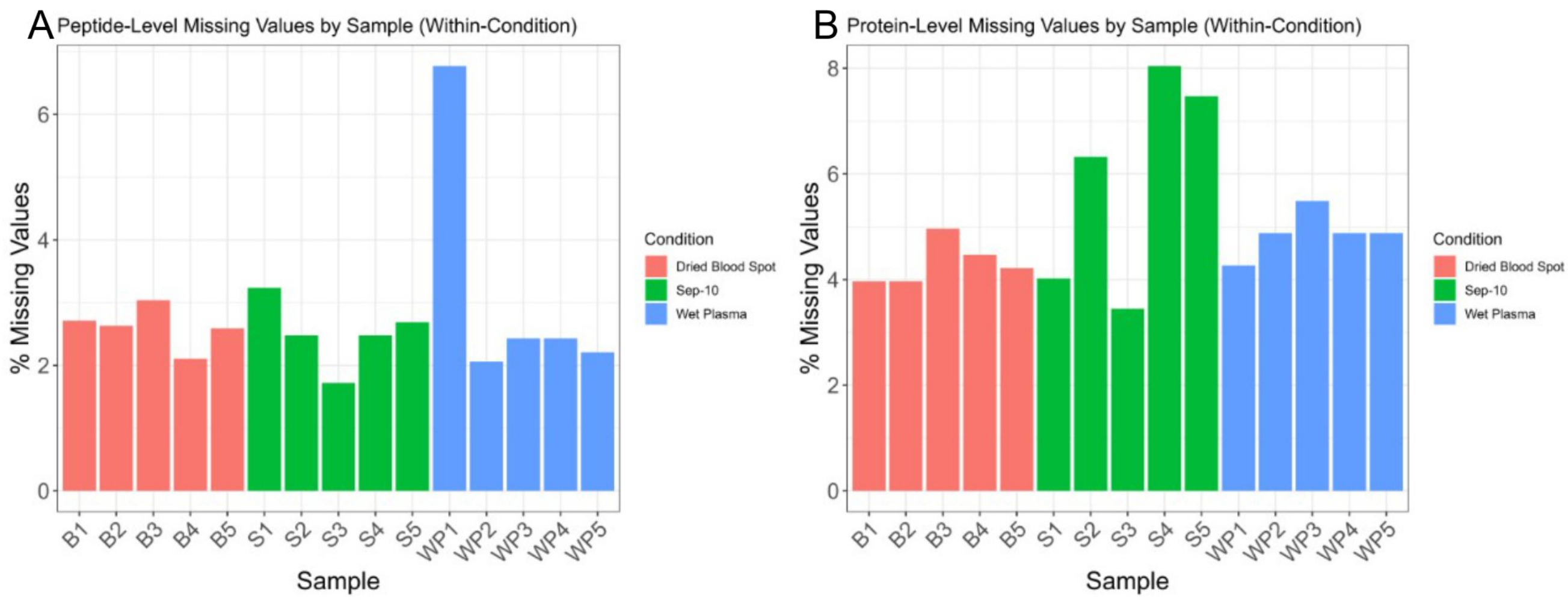

Figure S19- Analysis of cell-free DBS compared to traditional DBS and plasma samples. (A) Bar chart showing the percentage of missing protein values within each condition using each sample type (n=5). (B) Bar chart showing the percentage of missing protein values within each condition using each sample type (n=5).

Proof-of-Principle Clinical Validation in Chronic Kidney Disease

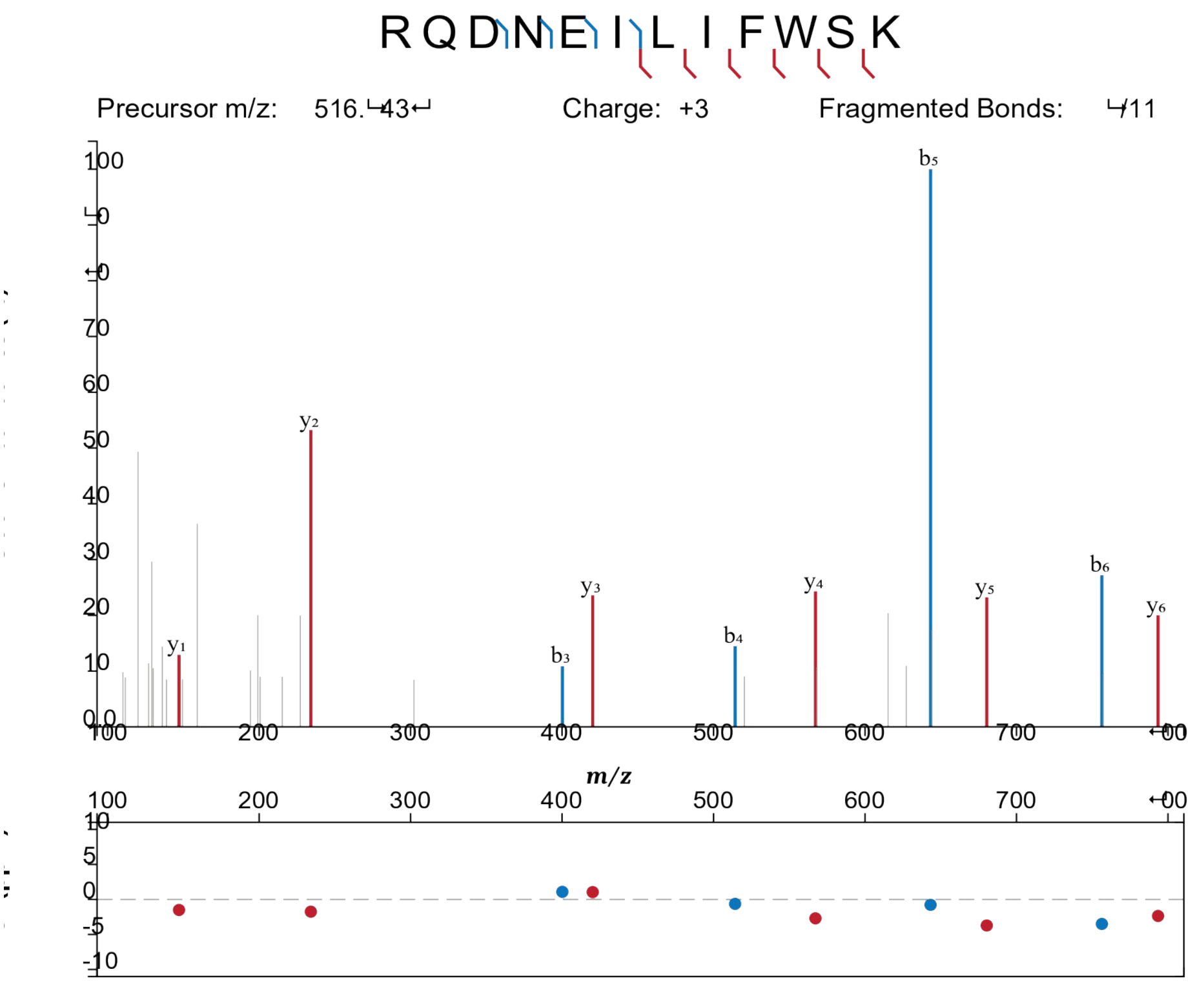

Figure S20- Annotated MS/MS fragmentation spectrum for an identified peptide from C-Reactive Protein in dried blood spots.

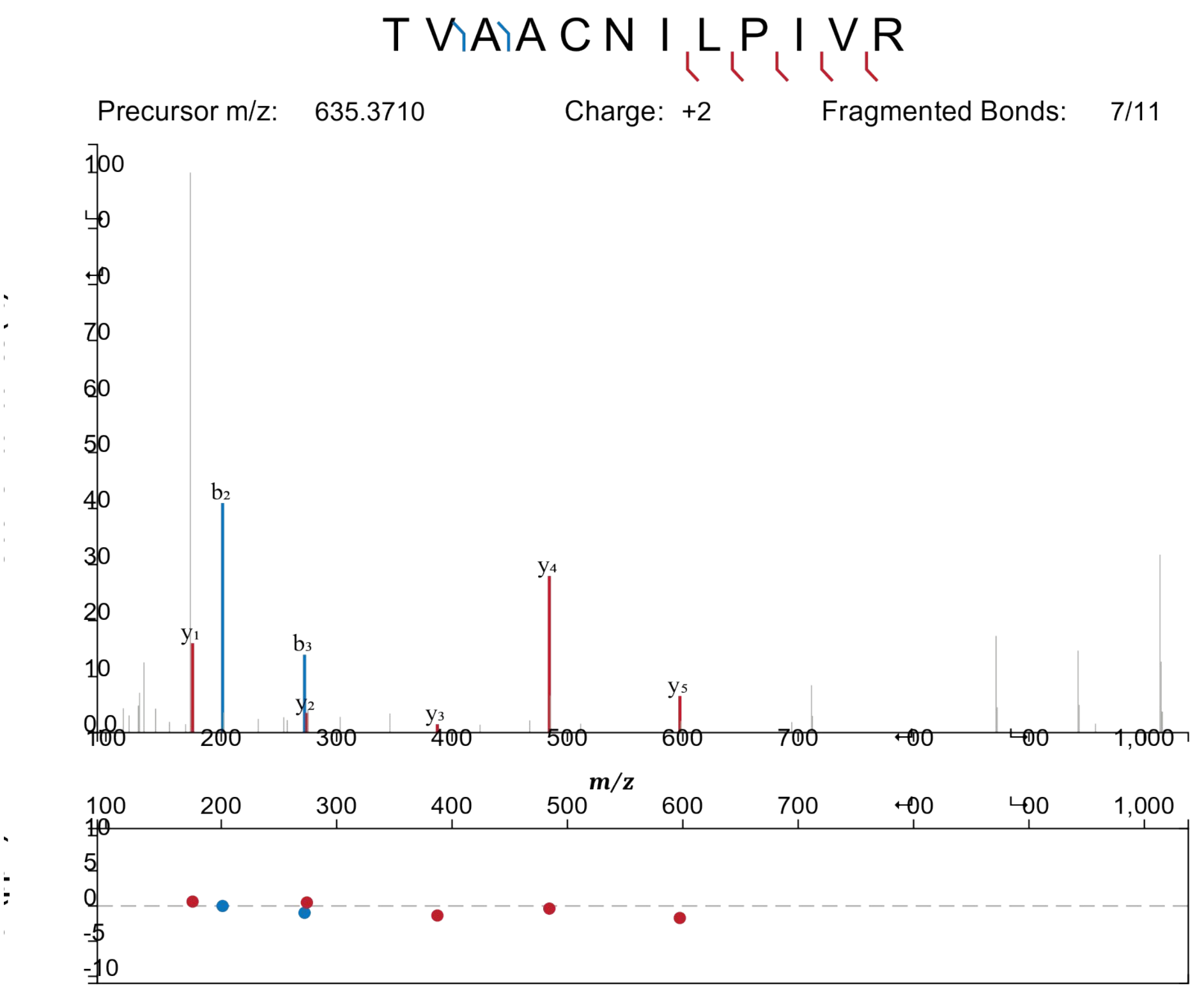

Figure S21- Annotated MS/MS fragmentation spectrum for an identified peptide from Protein AMBP in dried blood spots.

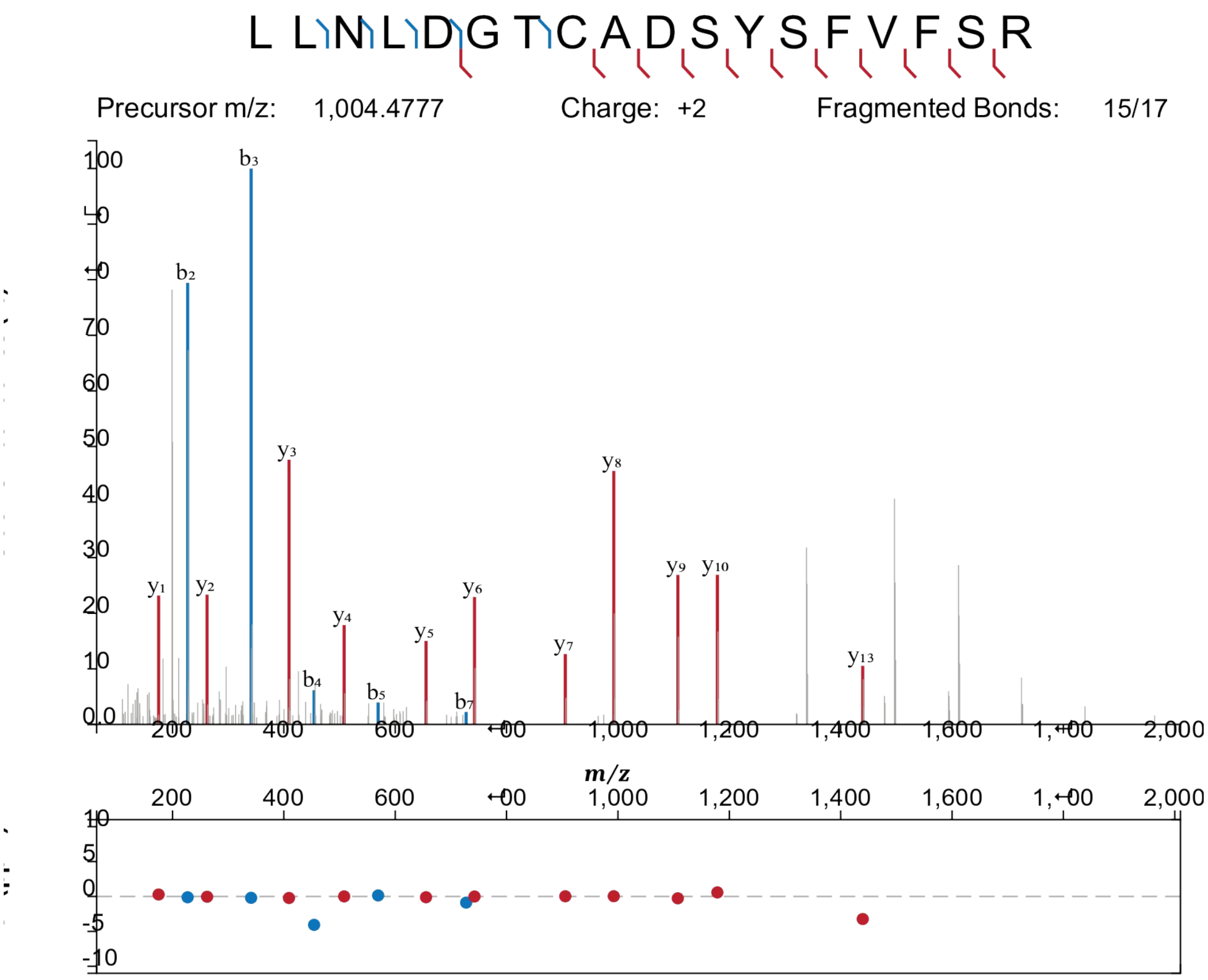

Figure S22- Annotated MS/MS fragmentation spectrum for an identified peptide from Retinol Binding Protein 4 in dried blood spots.

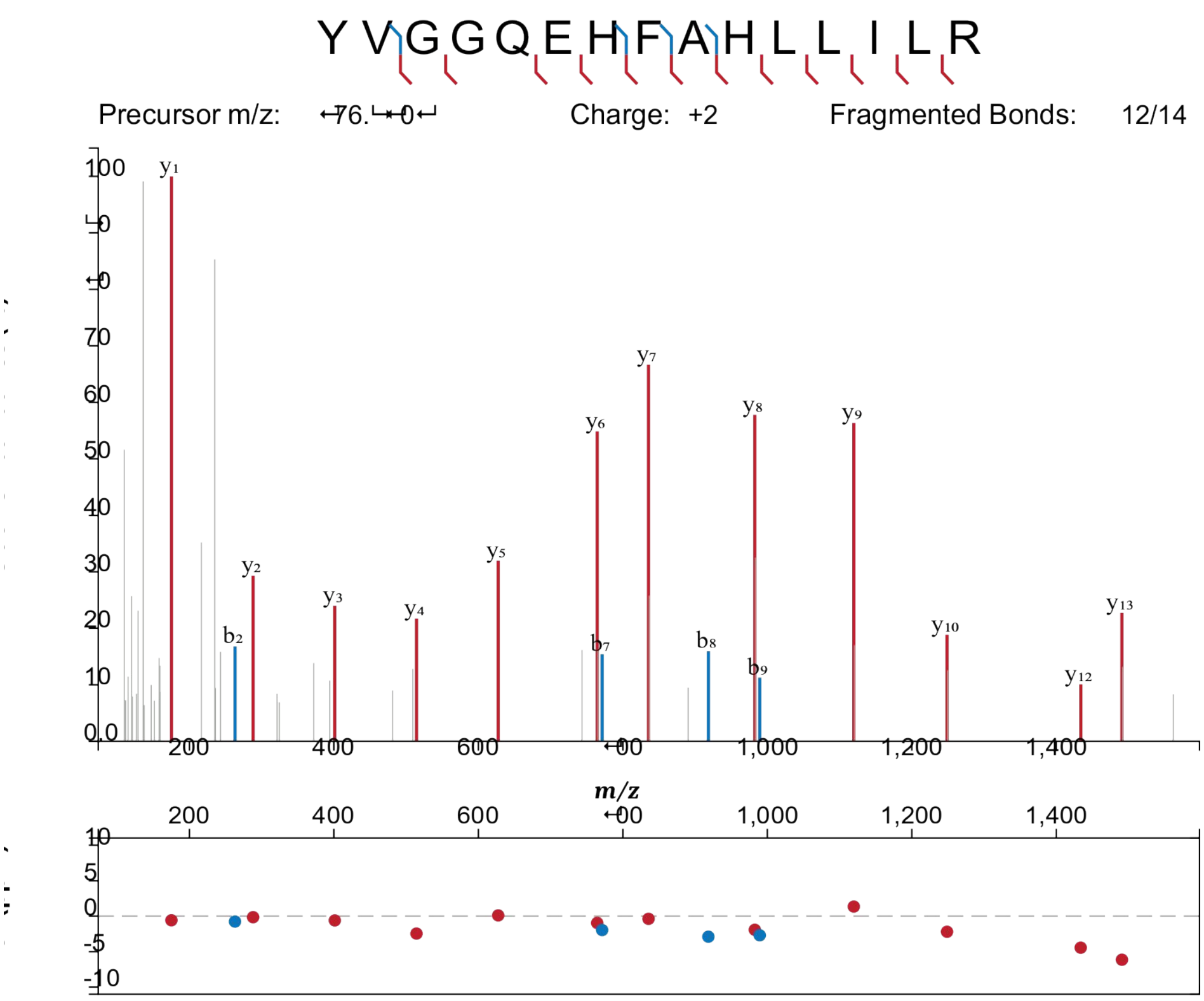

Figure S23- Annotated MS/MS fragmentation spectrum for an identified peptide from alpha-1-acid glycoprotein 1in dried blood spots.

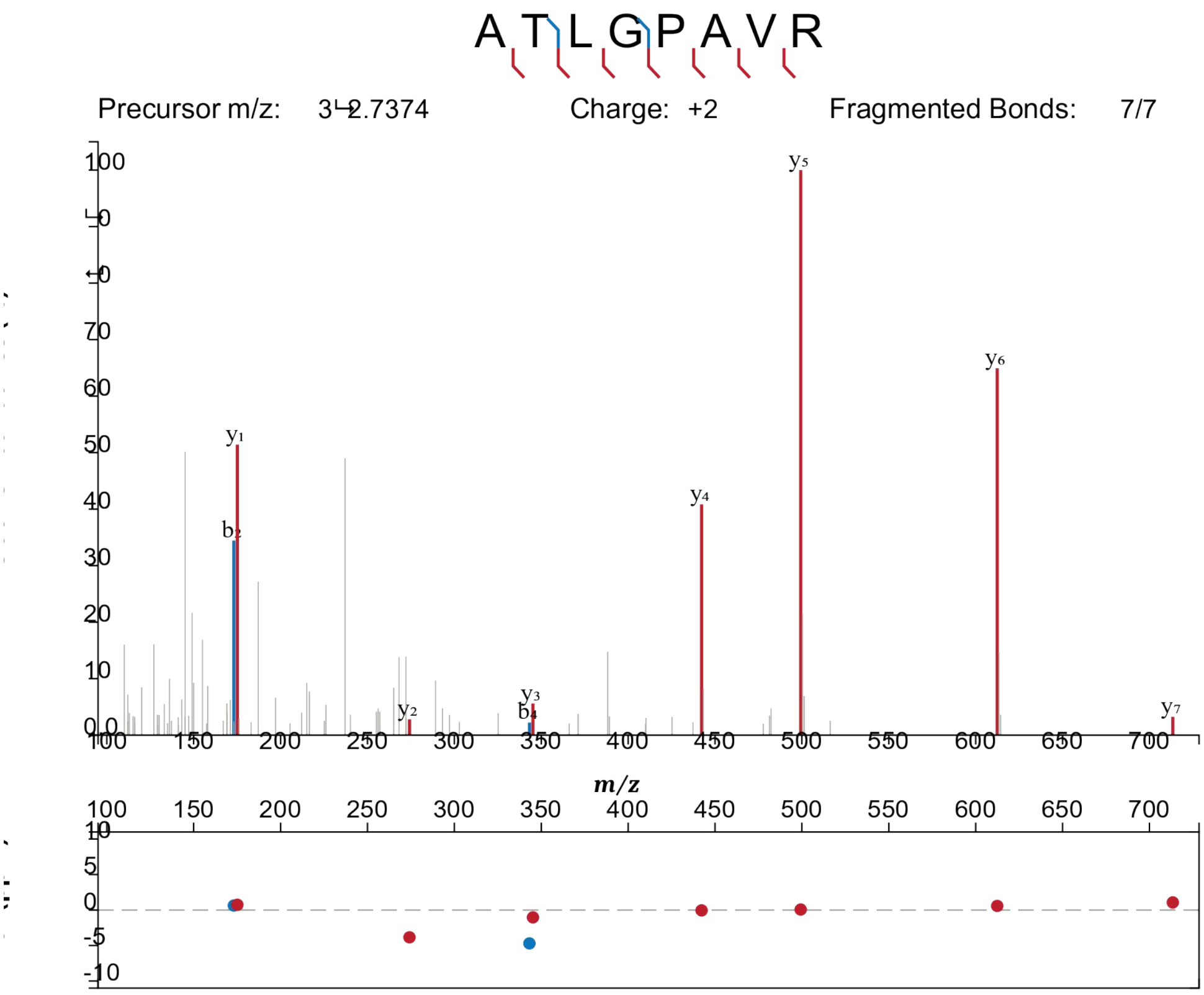

Figure S24- Annotated MS/MS fragmentation spectrum for an identified peptide from complement factor D in dried blood spots.

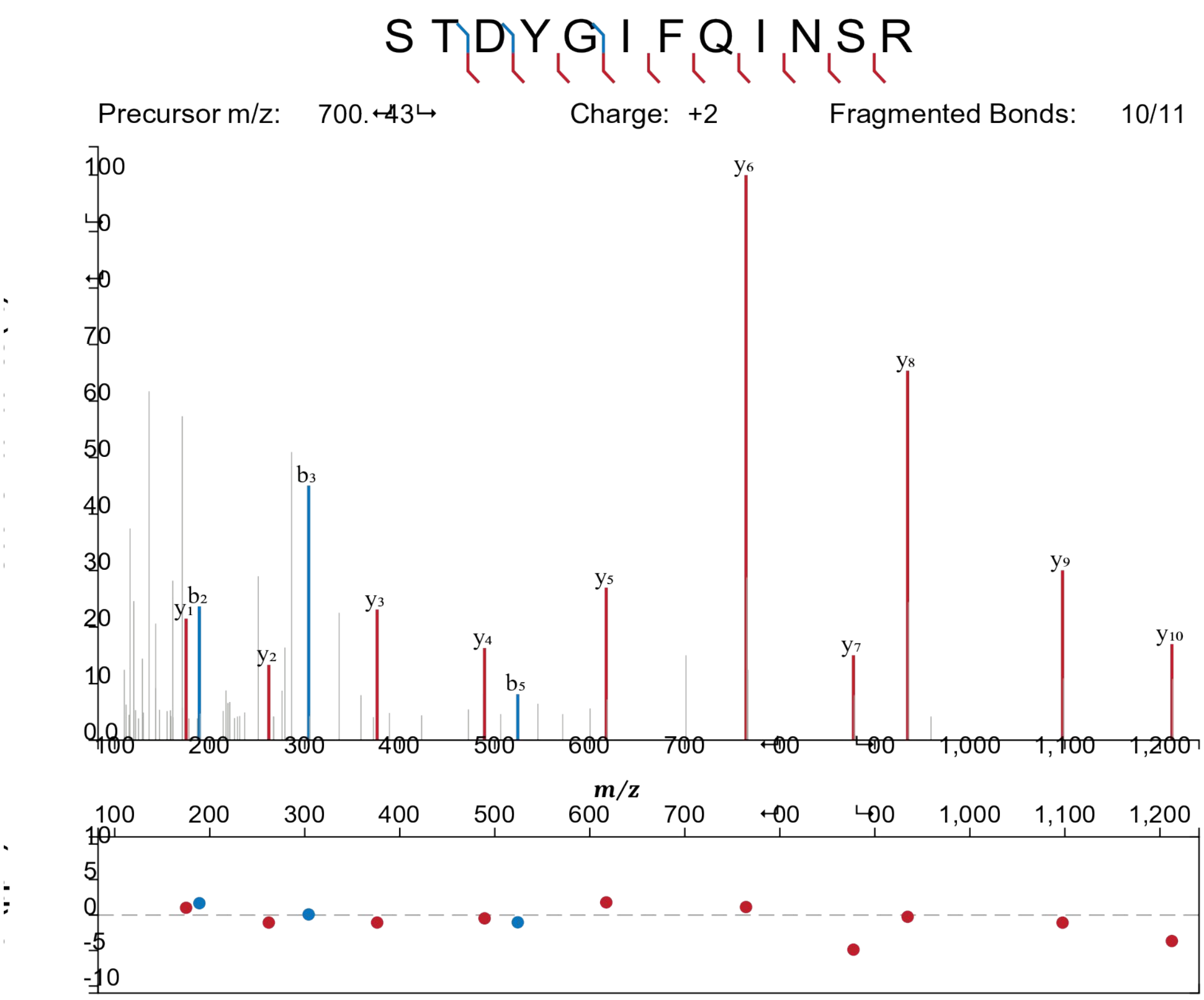

Figure S25- Annotated MS/MS fragmentation spectrum for an identified peptide from lysozyme C in dried blood spots.

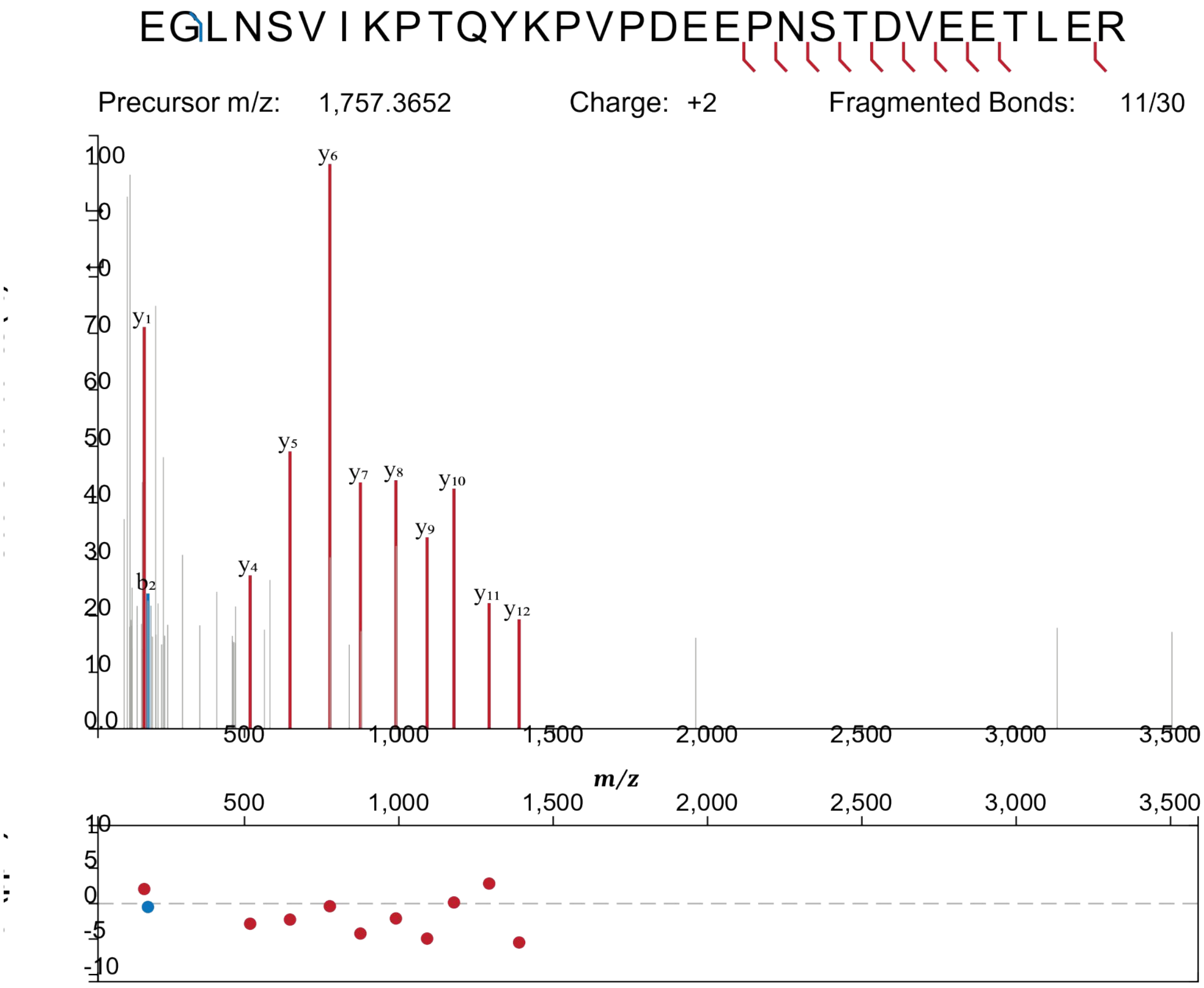

Figure S26- Annotated MS/MS fragmentation spectrum for an identified peptide from tropomodulin 1 in dried blood spots.

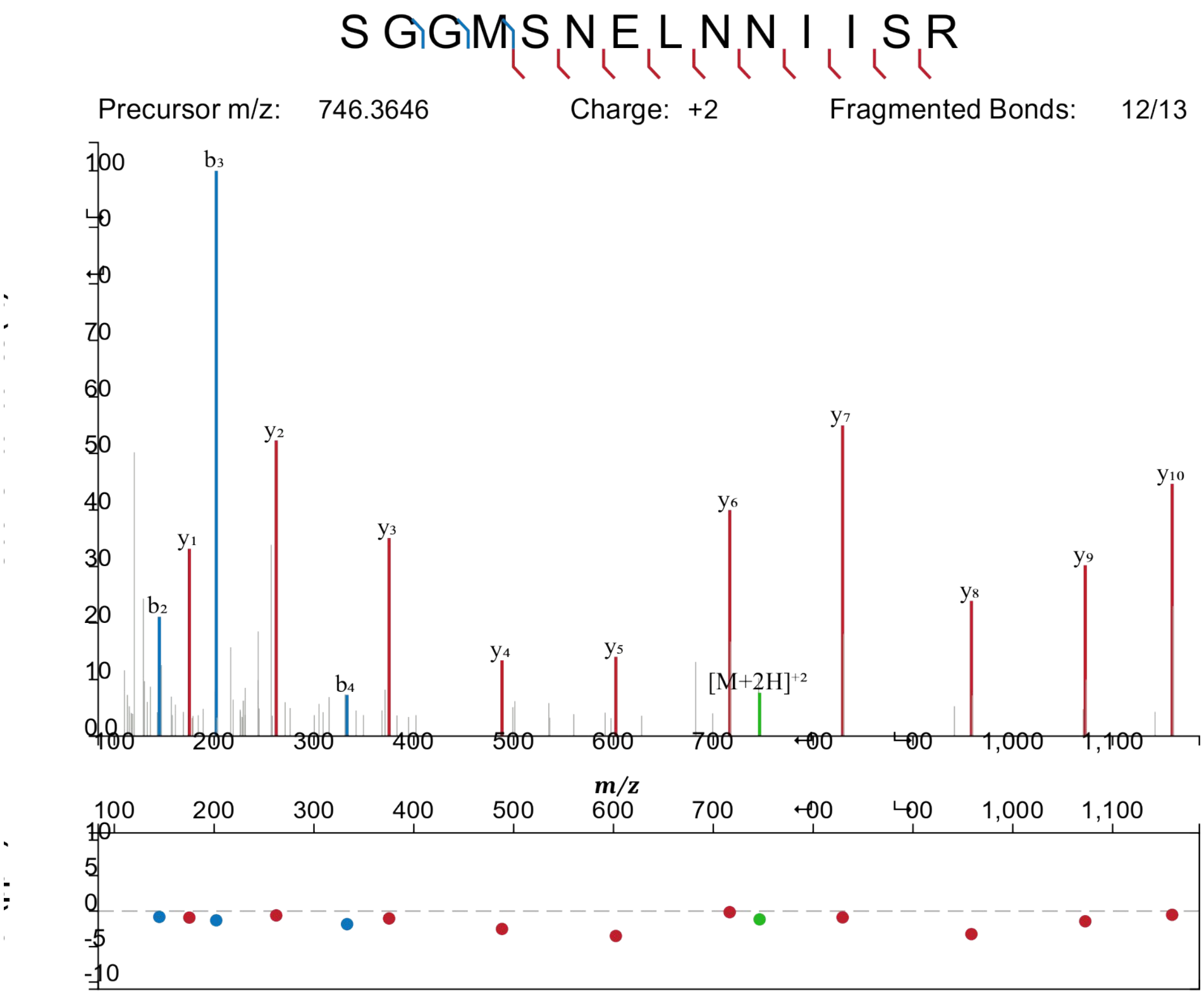

Figure S27- Annotated MS/MS fragmentation spectrum for an identified peptide from ATP citrate lyase in dried blood spots.

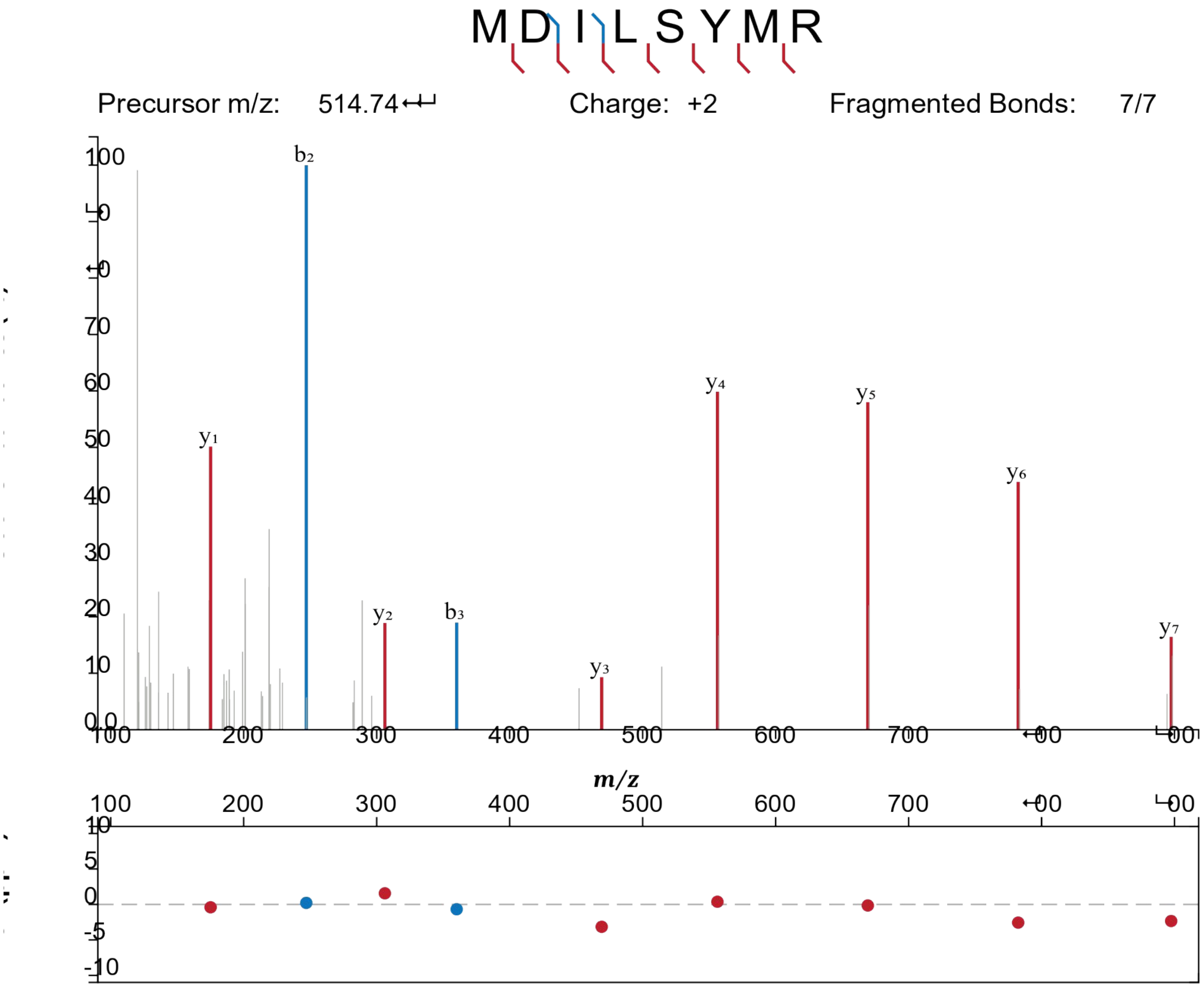

Figure S28- Annotated MS/MS fragmentation spectrum for an identified peptide from glutathione peroxidase 3 in dried blood spots.

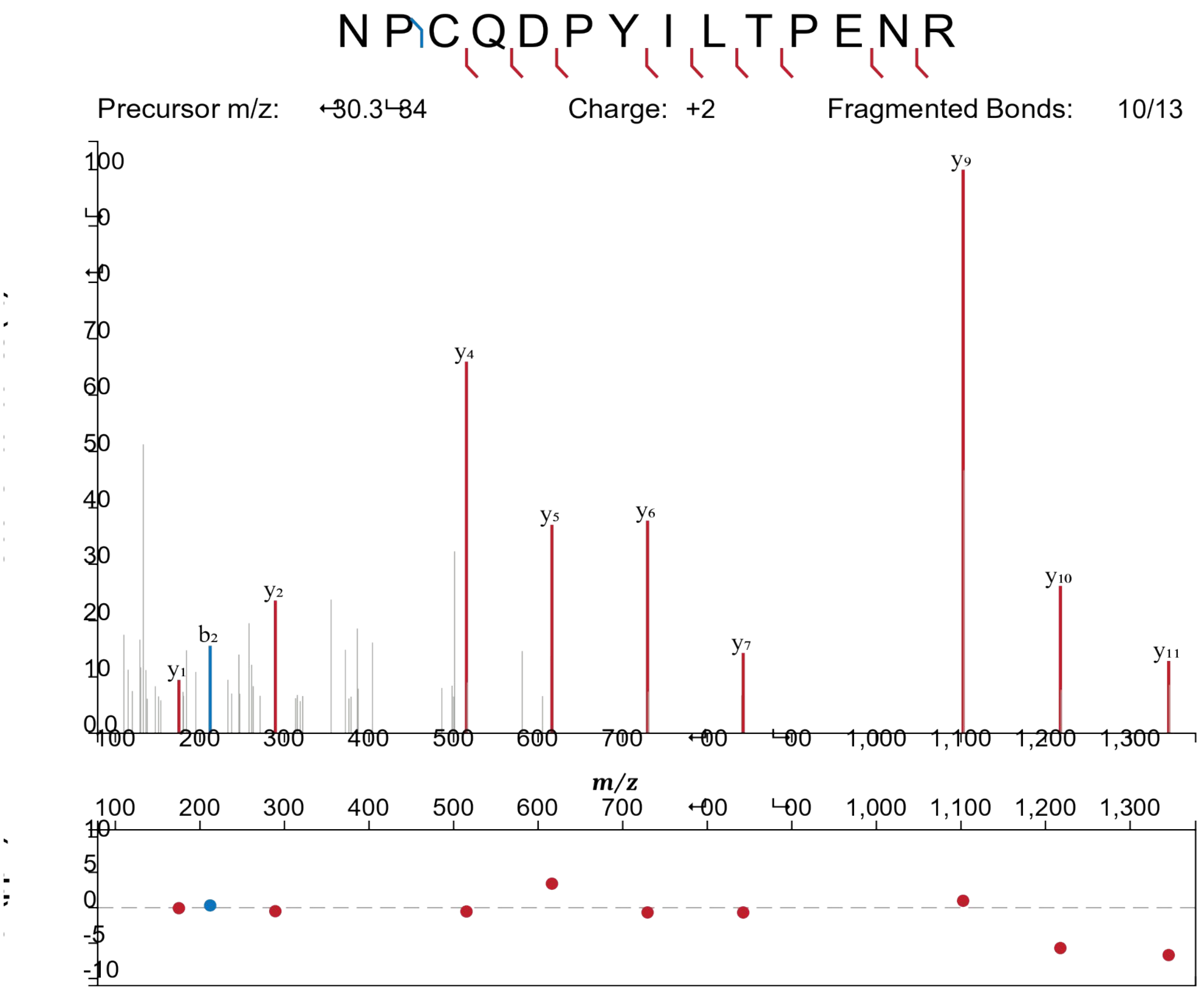

Figure S29- Annotated MS/MS fragmentation spectrum for an identified peptide from EGF-containing fibulin-like extracellular matrix protein 1 in dried blood spots.

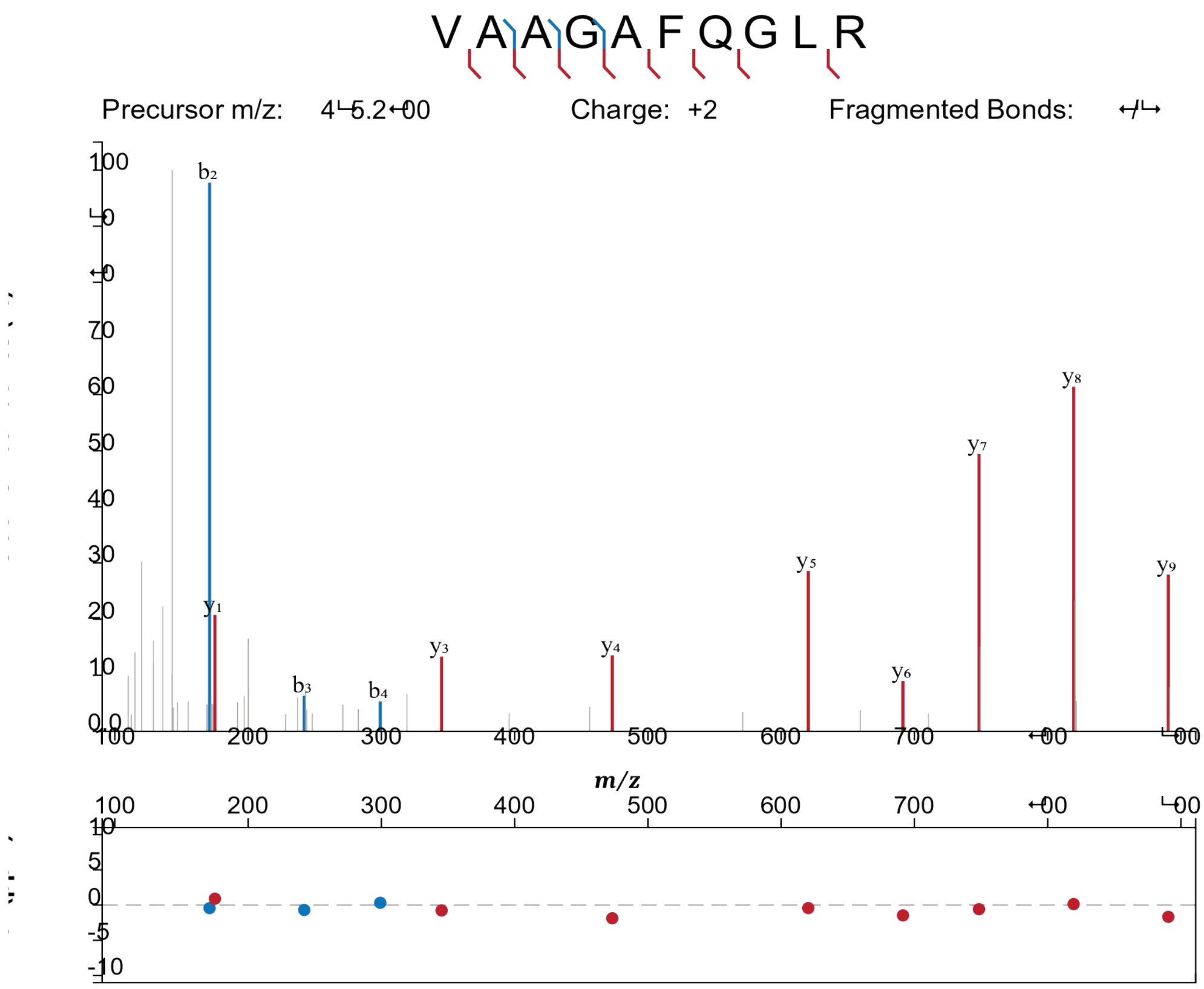

Figure S30- Annotated MS/MS fragmentation spectrum for an identified peptide from leucine-rich alpha-2-glycoprotein 1 in dried blood spots.

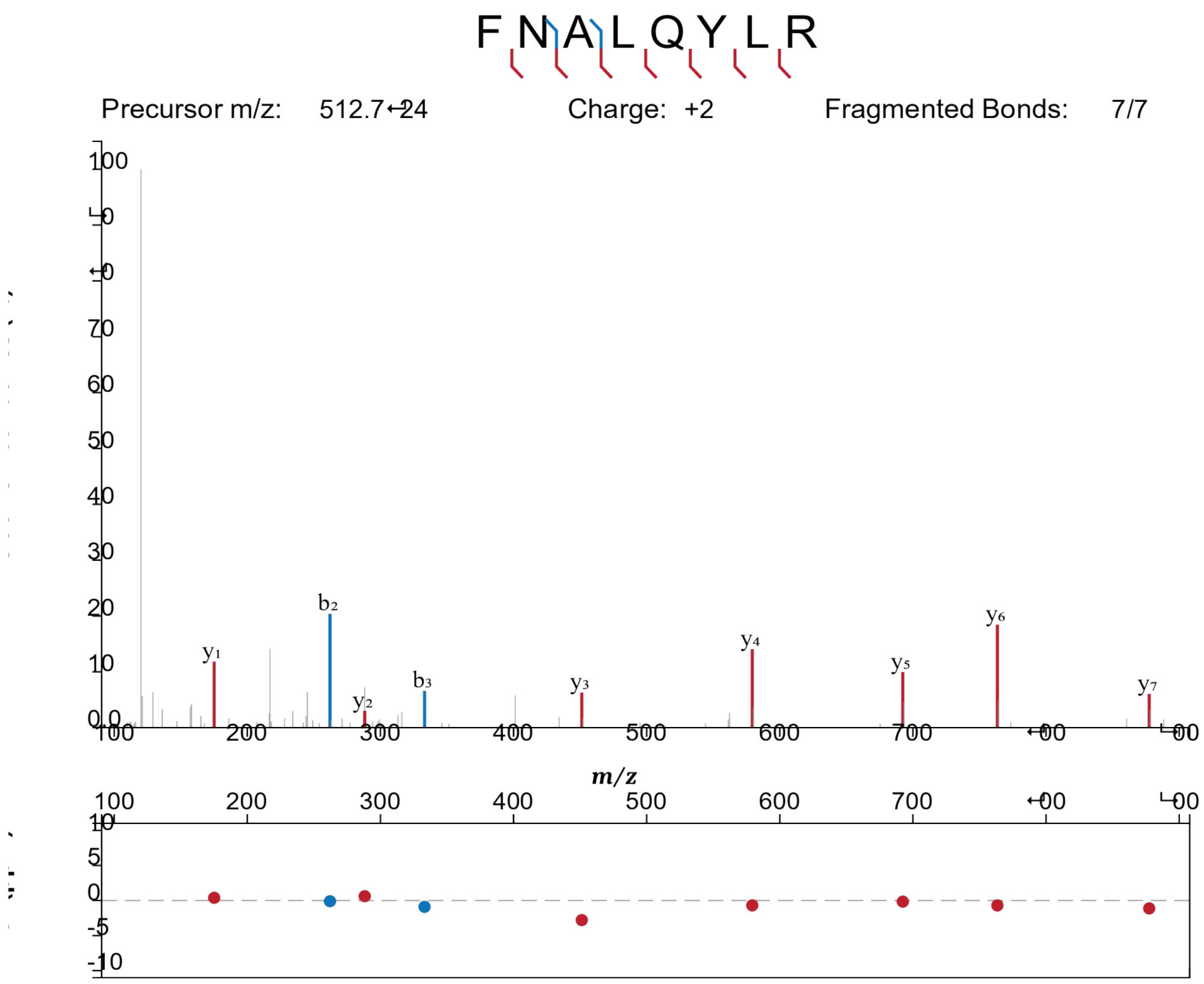

Figure S31- Annotated MS/MS fragmentation spectrum for an identified peptide from lumican in dried blood spots.

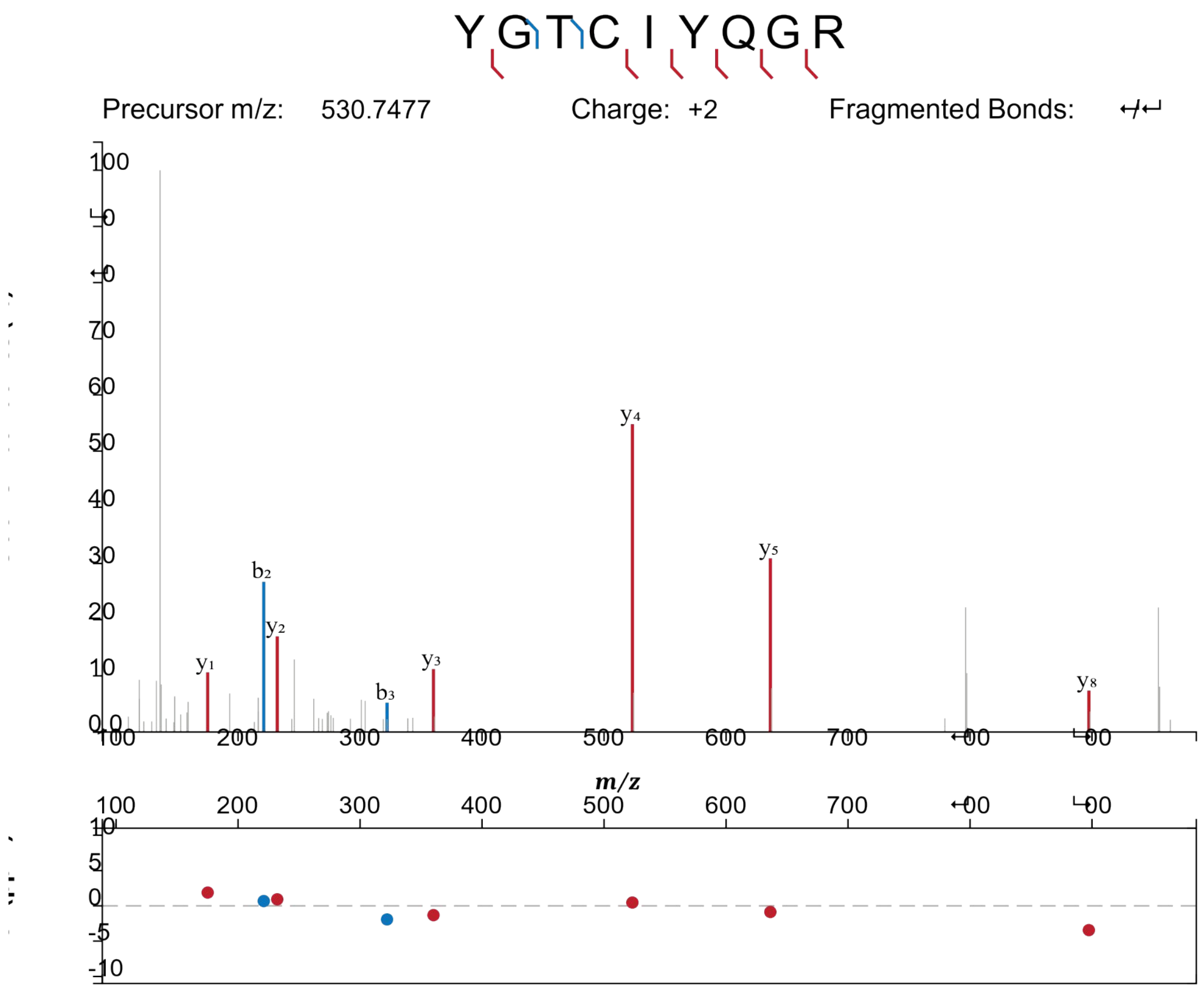

Figure S32- Annotated MS/MS fragmentation spectrum for an identified peptide from defensin alpha 1 in dried blood spots.

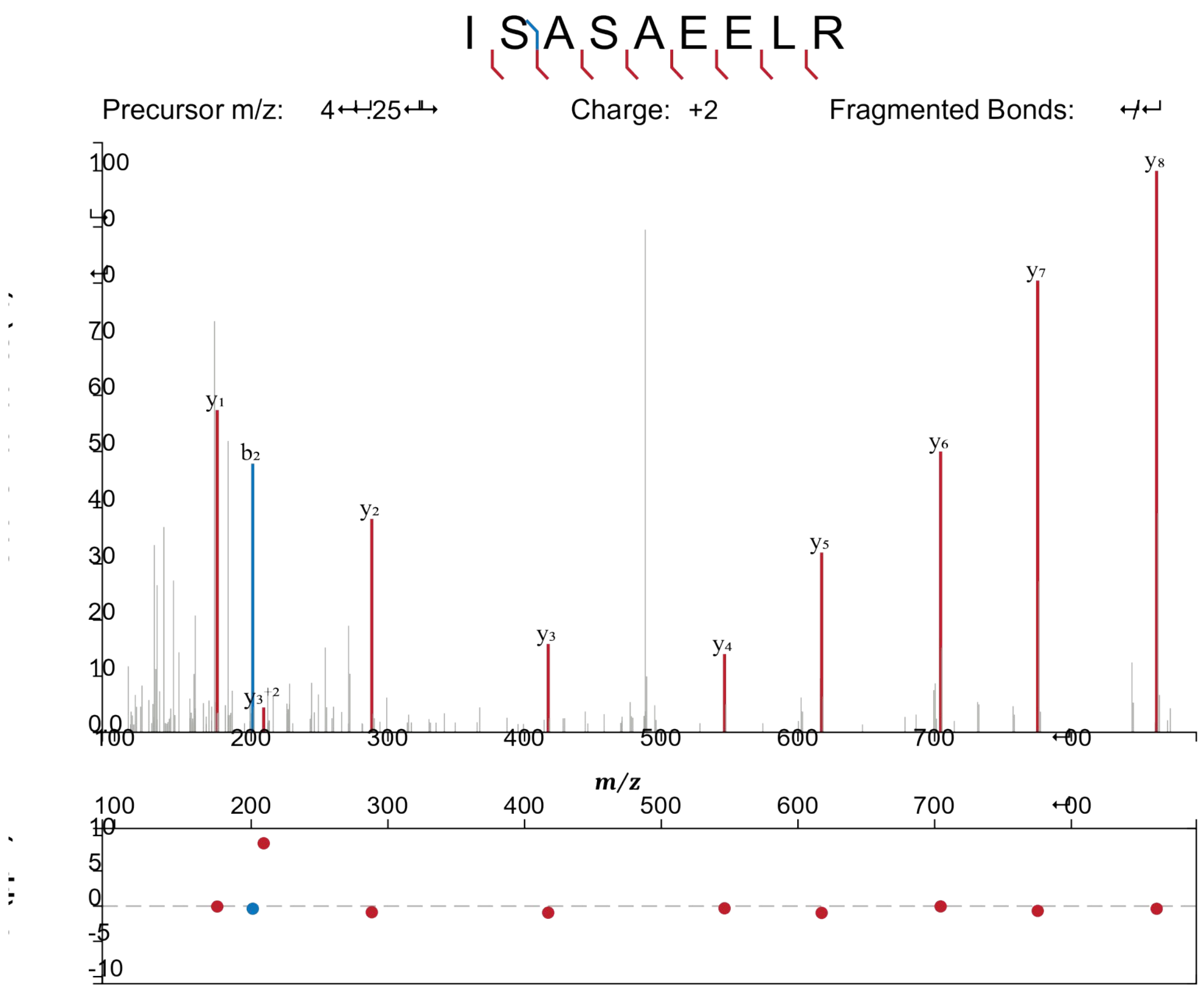

Figure S33- Annotated MS/MS fragmentation spectrum for an identified peptide from apolipoprotein A4 in dried blood spots.

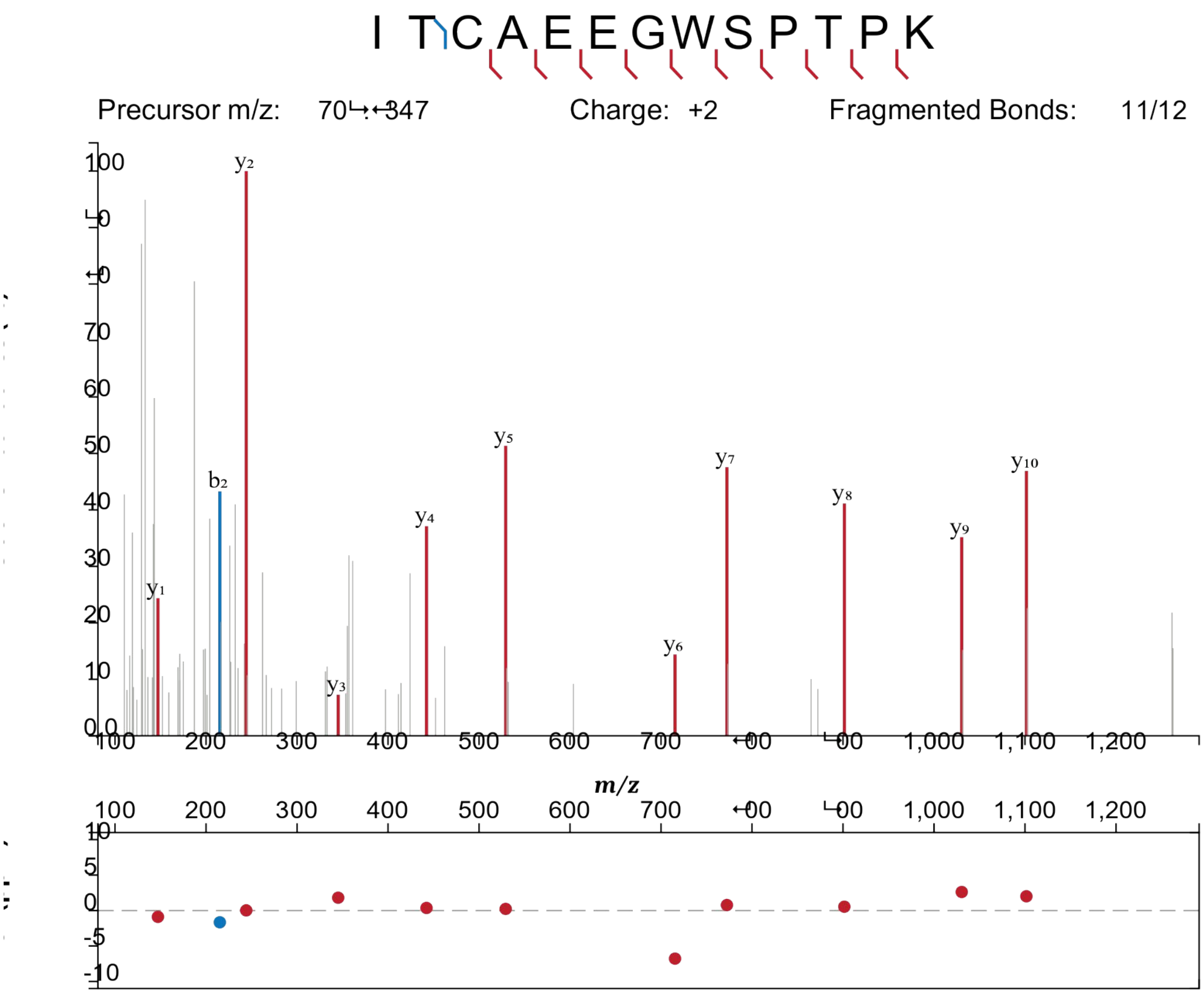

Figure S34- Annotated MS/MS fragmentation spectrum for an identified peptide from complement factor h related protein 2 in dried blood spots.

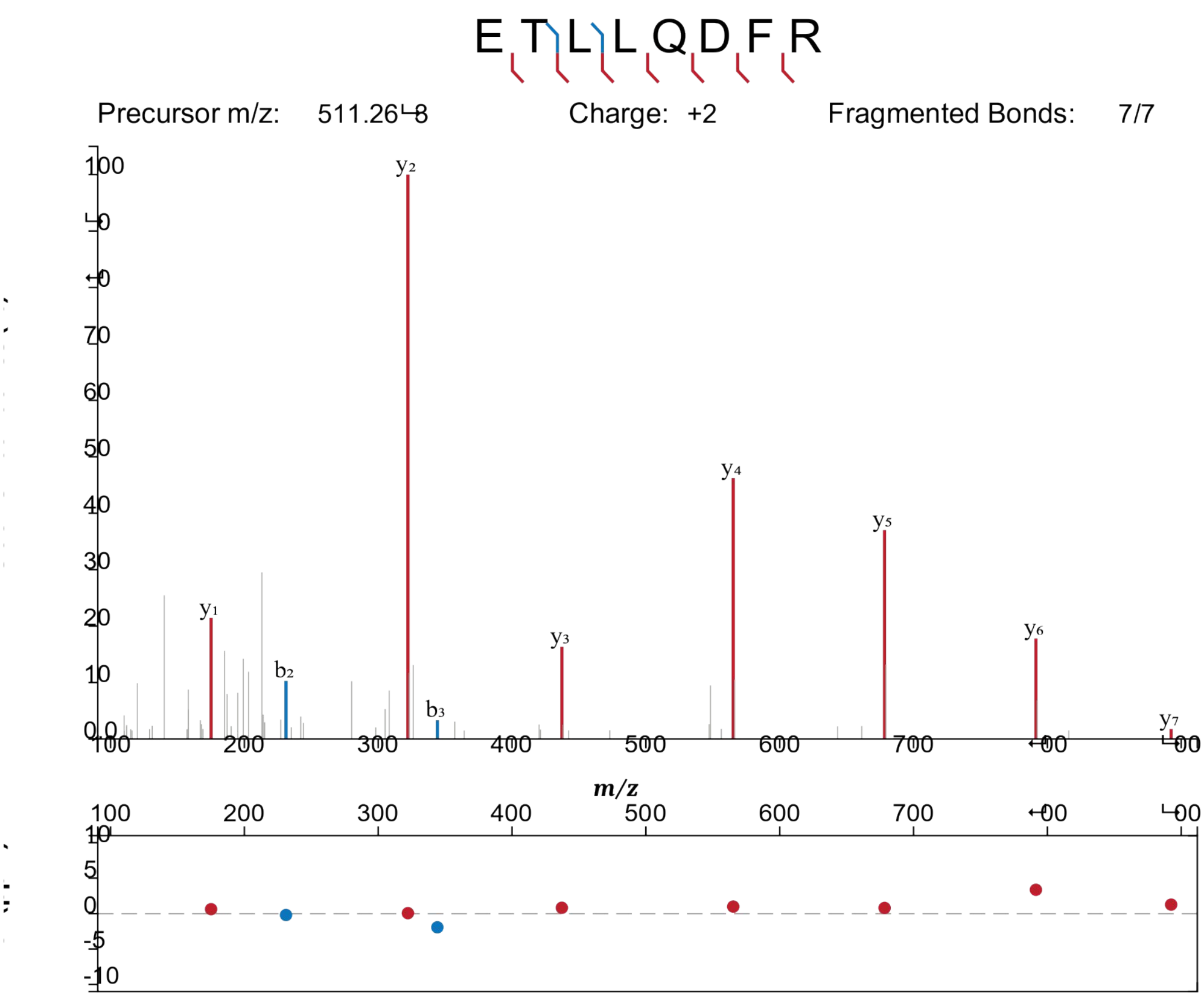

Figure S35- Annotated MS/MS fragmentation spectrum for an identified peptide from protein AMBP in plasma samples.

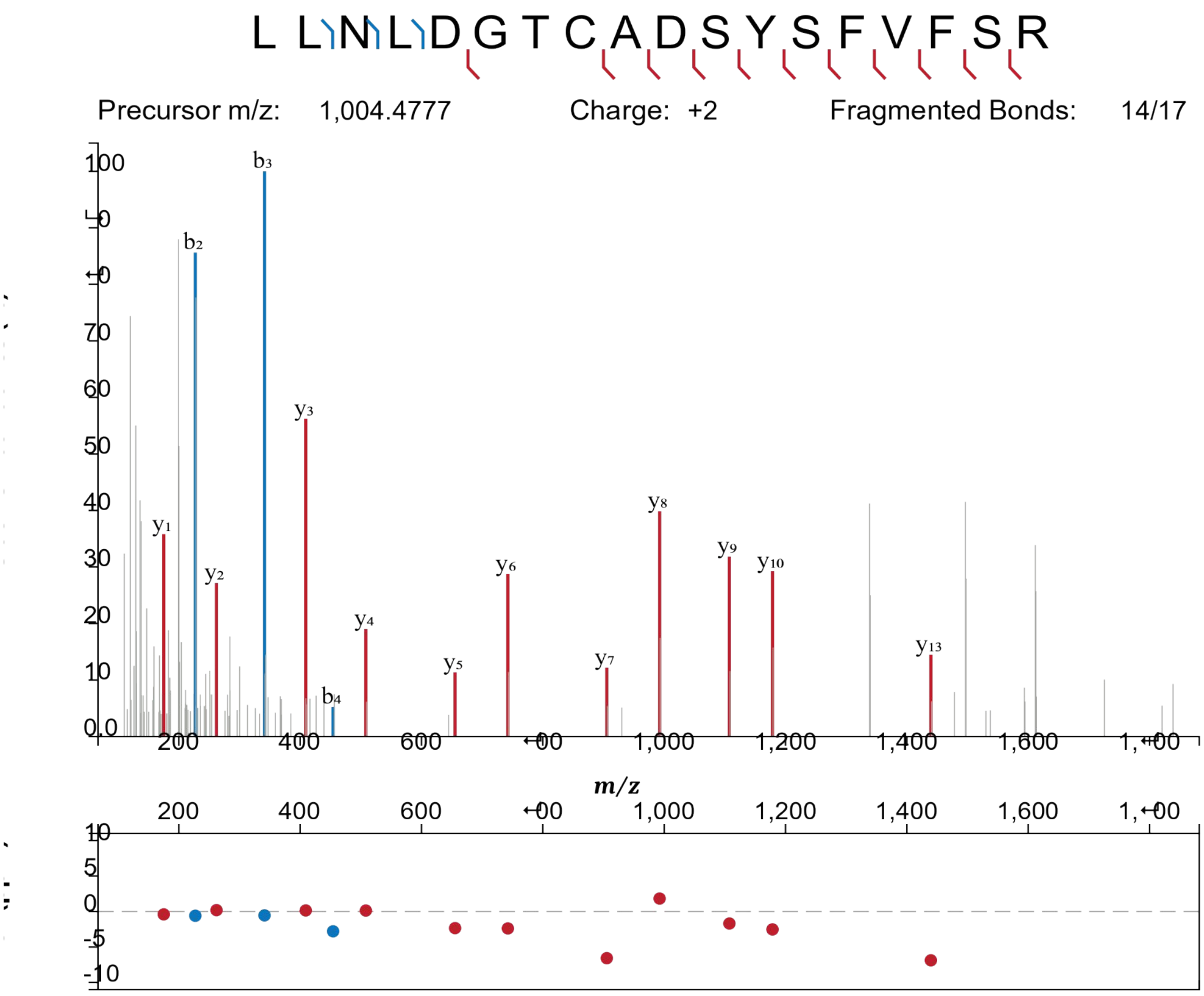

Figure S36- Annotated MS/MS fragmentation spectrum for an identified peptide from retinol binding protein 4 in plasma samples.

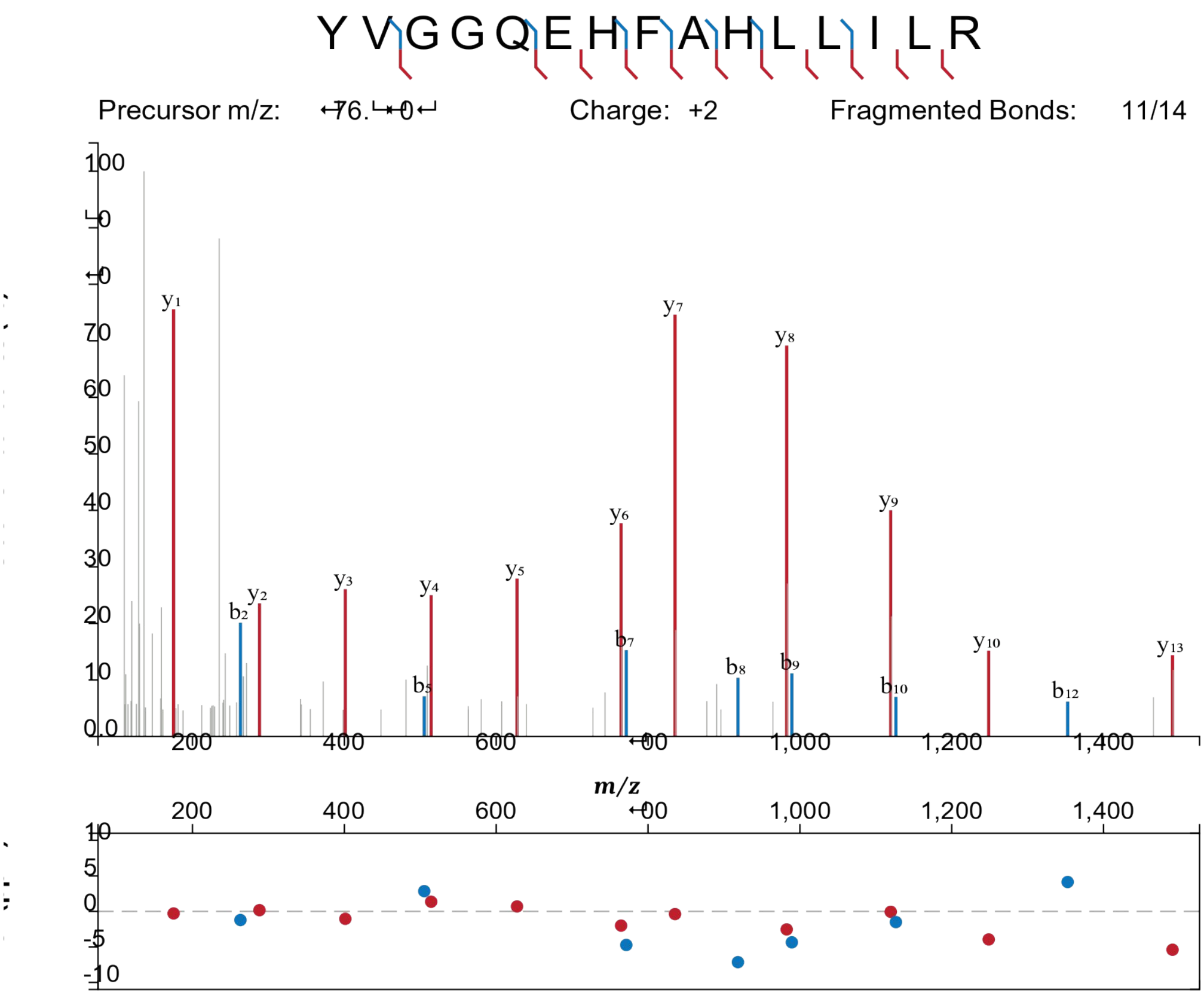

Figure S37- Annotated MS/MS fragmentation spectrum for an identified peptide from alpha 1 acid glycoprotein 1 in plasma samples.

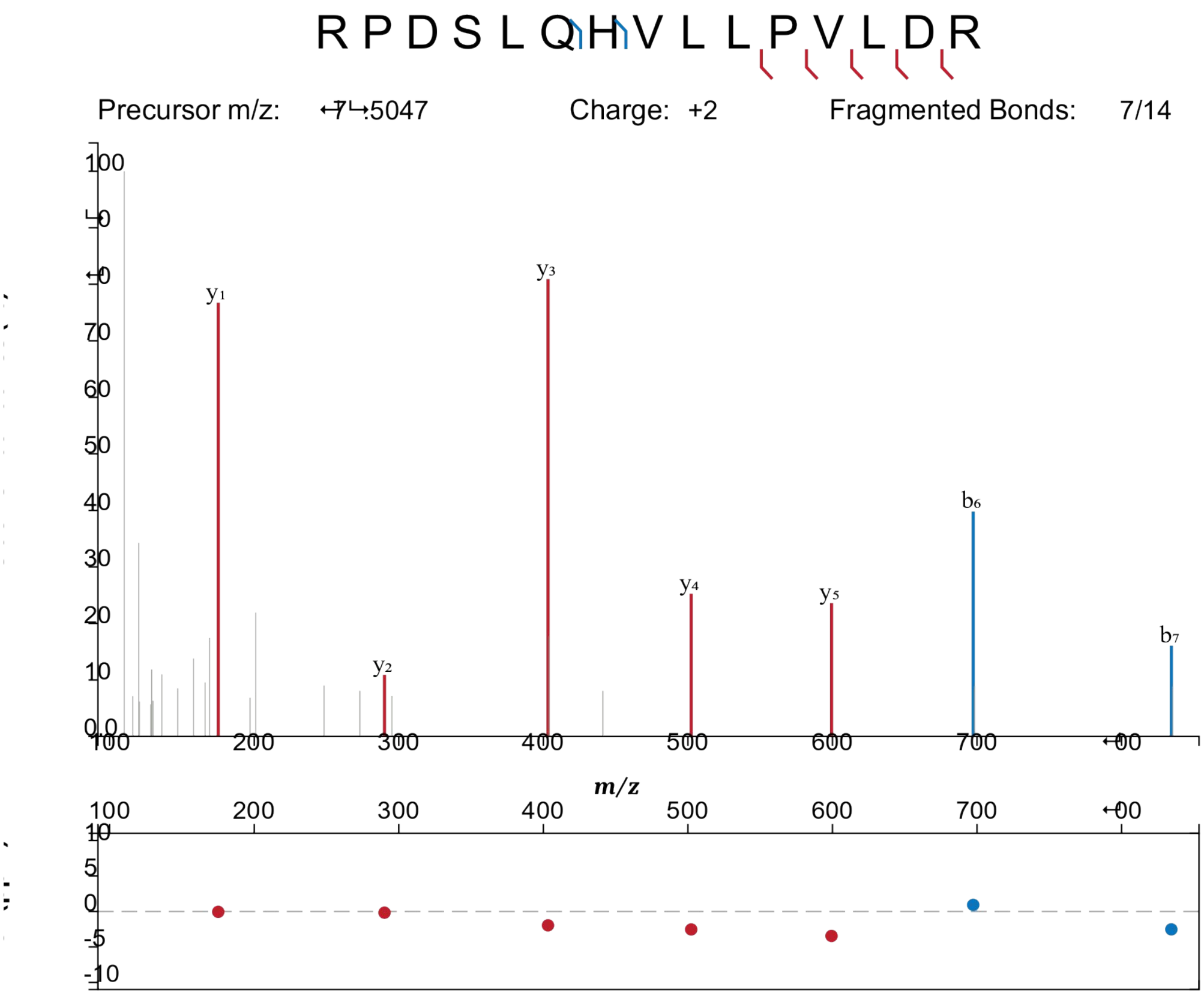

Figure S38- Annotated MS/MS fragmentation spectrum for an identified peptide from complement factor D in plasma samples.

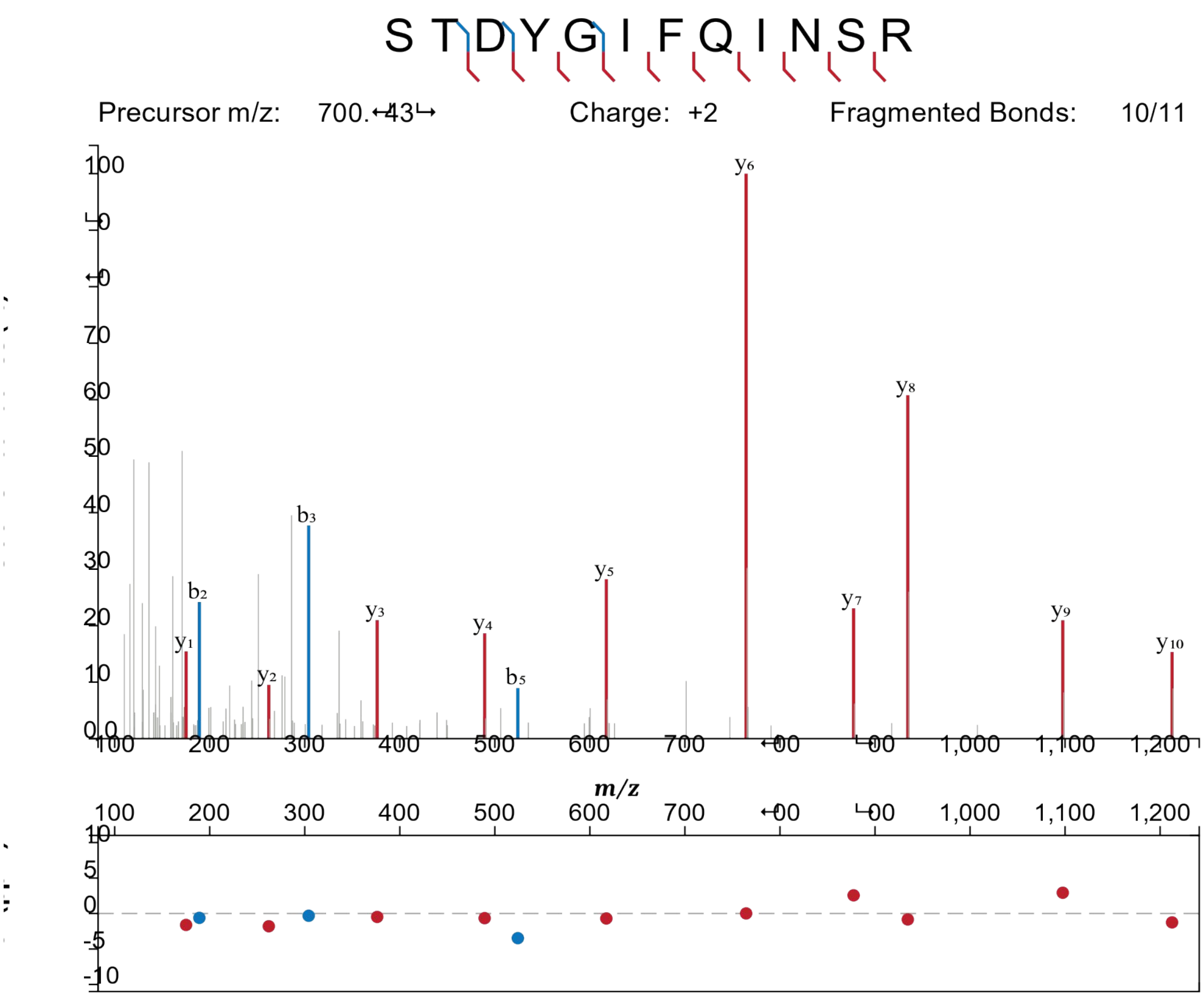

Figure S39- Annotated MS/MS fragmentation spectrum for an identified peptide from lysozyme C in plasma samples.

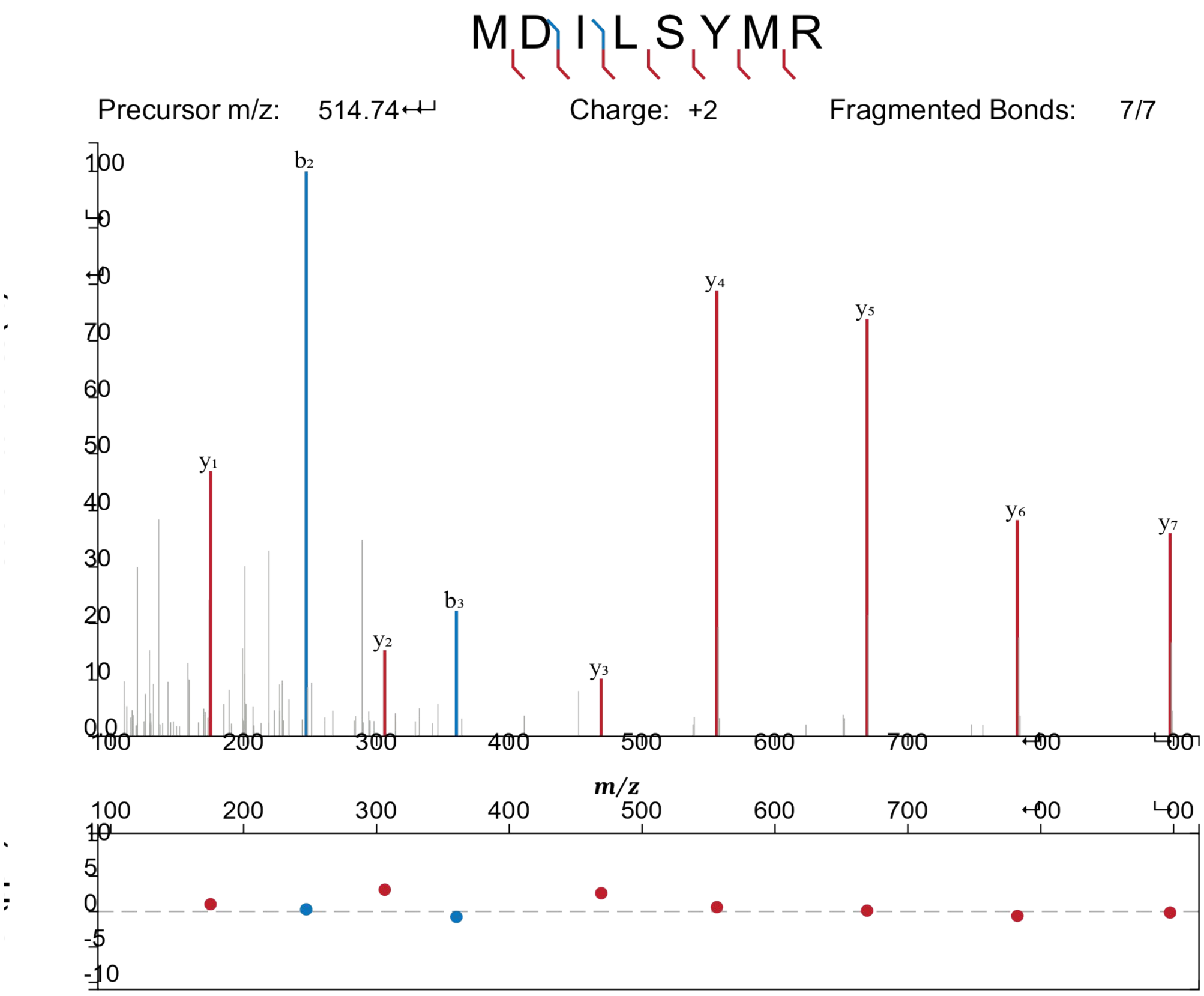

Figure S40- Annotated MS/MS fragmentation spectrum for an identified peptide from glutathione peroxidase 3 in plasma samples.

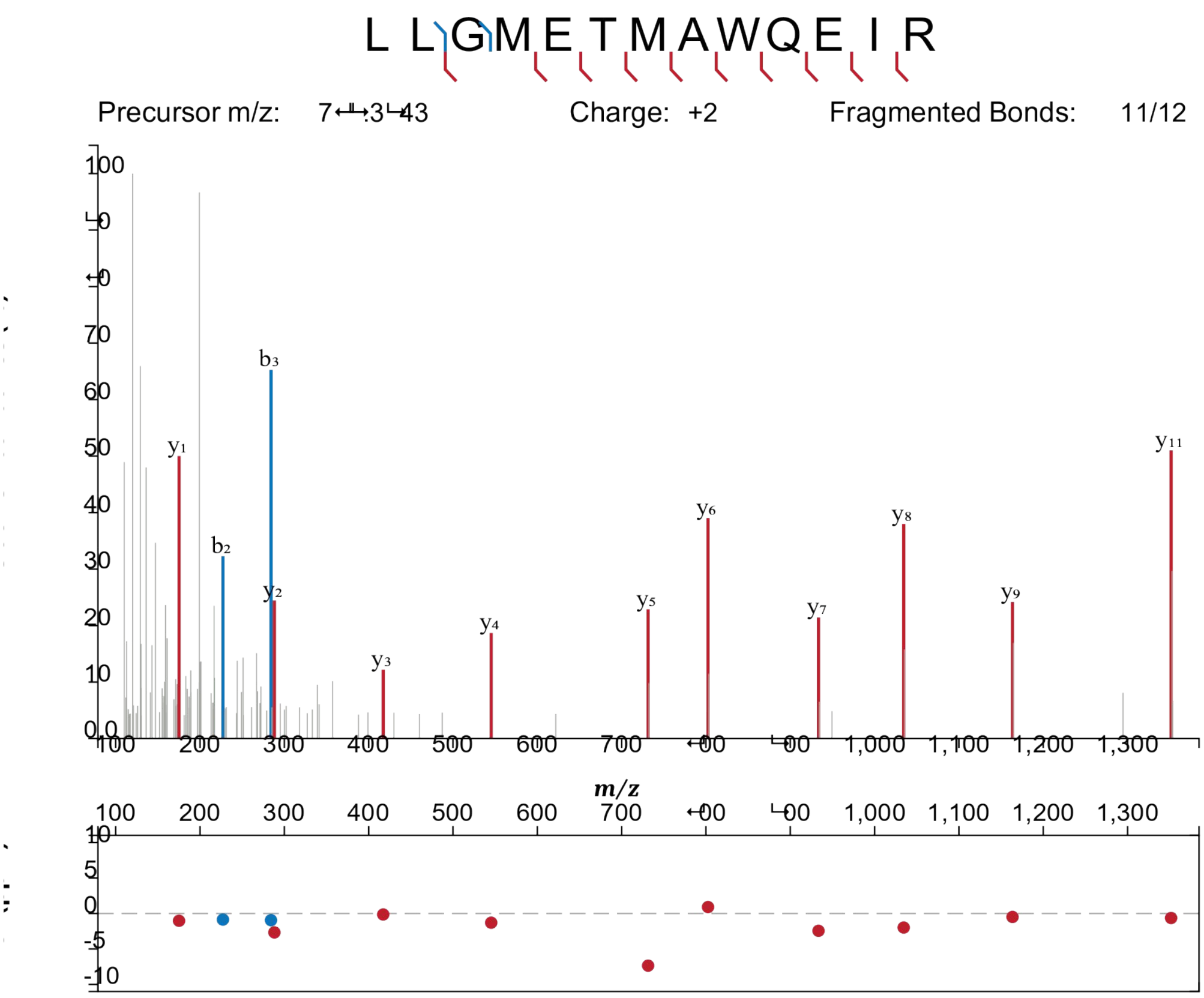

Figure S41- Annotated MS/MS fragmentation spectrum for an identified peptide from complement 2 in plasma samples.

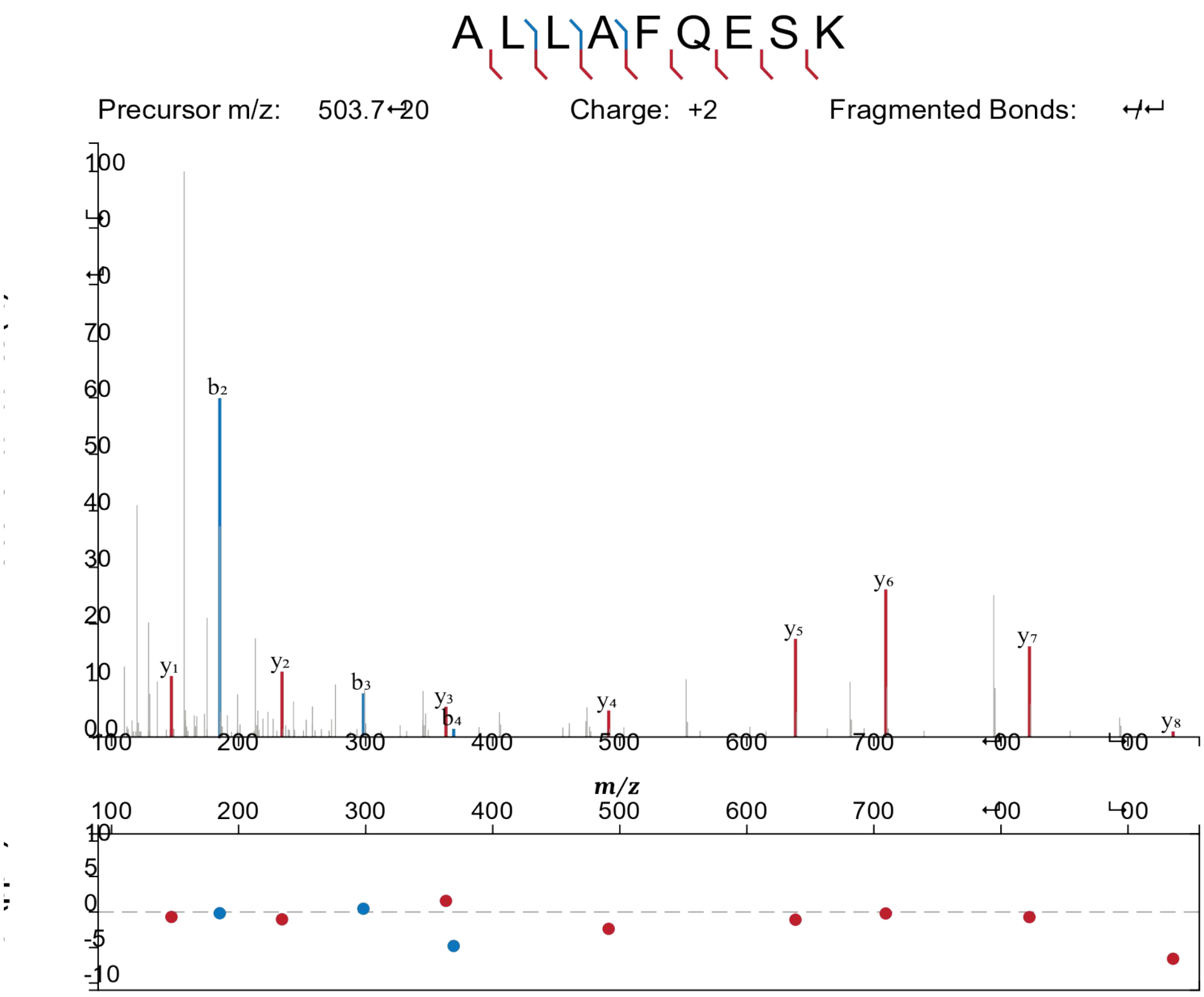

Figure S42- Annotated MS/MS fragmentation spectrum for an identified peptide from complement 4 binding protein in plasma samples.

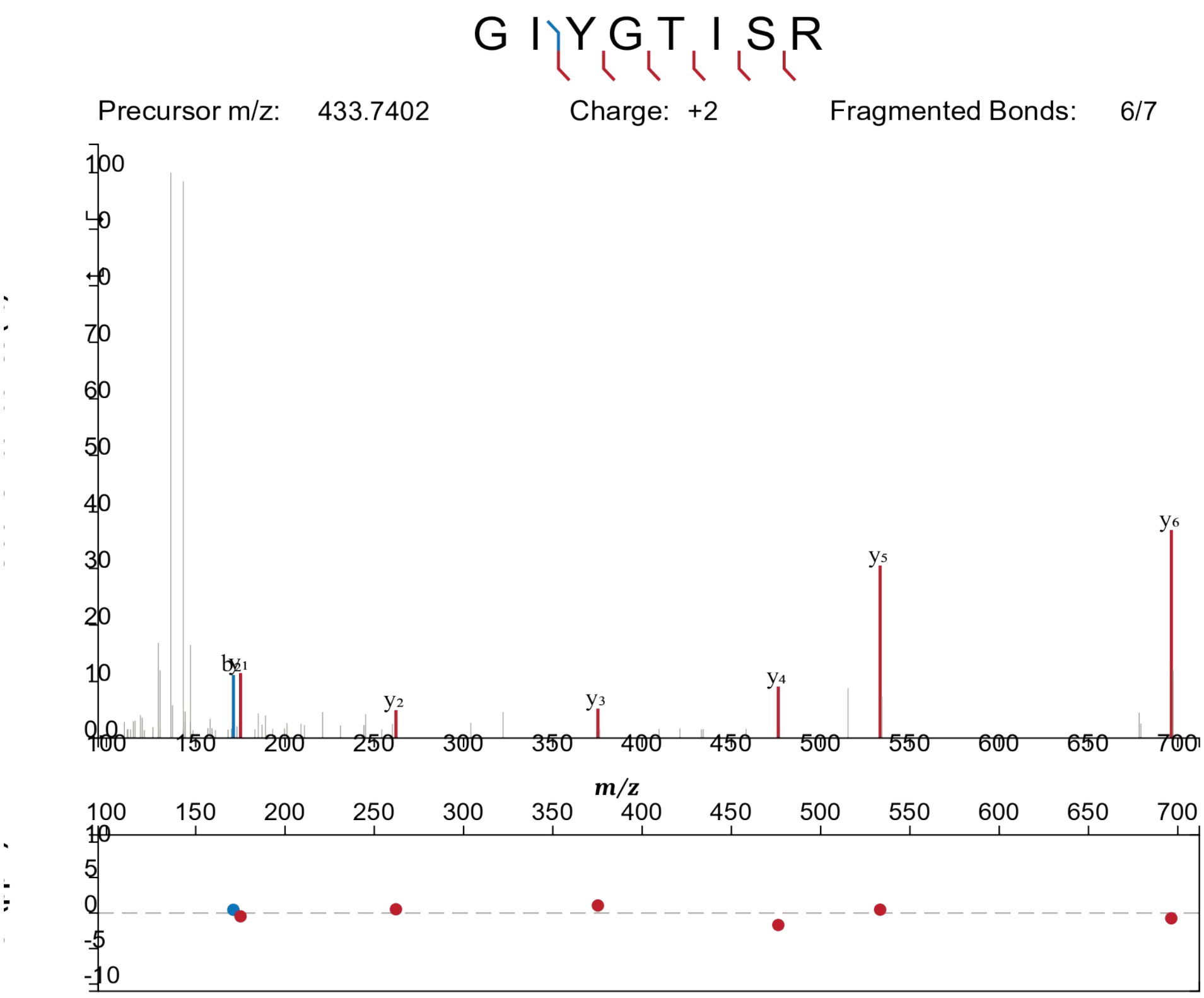

Figure S43- Annotated MS/MS fragmentation spectrum for an identified peptide from complement 5 in plasma samples.

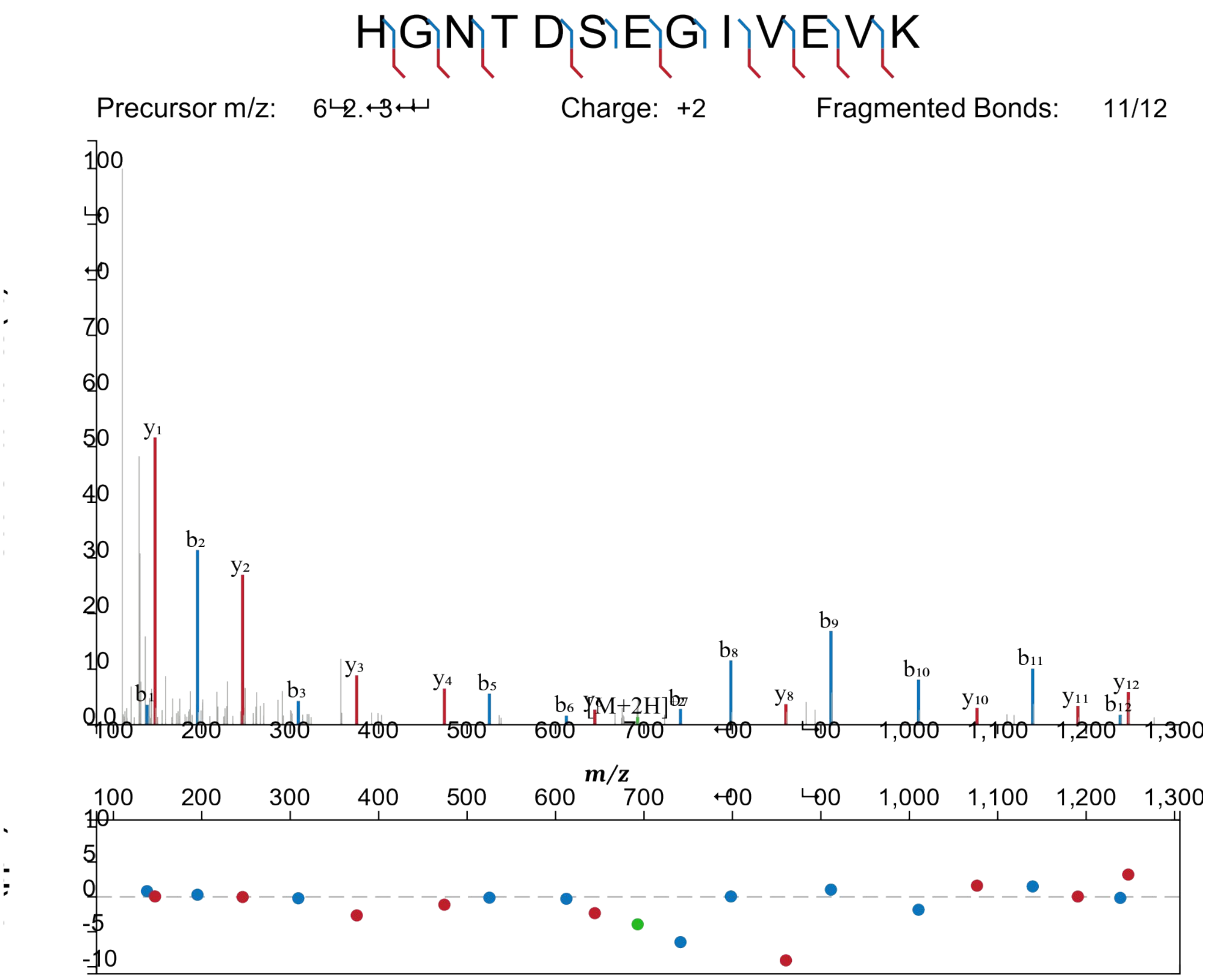

Figure S44- Annotated MS/MS fragmentation spectrum for an identified peptide from complement factor I in plasma samples.
